# Bone Morphogenetic Protein 4 Alleviates DSS-Induced Ulcerative Colitis Through Activating Intestinal Stem Cell by Target ID3

**DOI:** 10.3389/fcell.2021.700864

**Published:** 2021-10-06

**Authors:** Lei Hu, Junji Xu, Xue Wang, Liang Feng, Chunmei Zhang, Jinsong Wang, Songlin Wang

**Affiliations:** ^1^Salivary Gland Disease Center, Capital Medical University School of Stomatology, Beijing, China; ^2^Beijing Laboratory of Oral Health, Capital Medical University, Beijing, China; ^3^Immunology Research Center for Oral and Systemic Health, Beijing Friendship Hospital, Capital Medical University, Beijing, China; ^4^Department of Prosthodontics, Capital Medical University School of Stomatology, Beijing, China; ^5^Department of Periodontics, Capital Medical University School of Stomatology, Beijing, China; ^6^Department of Biochemistry and Molecular Biology, School of Basic Medical Sciences, Capital Medical University, Beijing, China

**Keywords:** ulcerative colitis, intestinal stem cell, BMP4, ID3, epithelium proliferation

## Abstract

Damage to intestinal epithelial cell proliferation or intestinal stem cell (ISC) maintenance may trigger inflammatory bowel disease (IBD), and protecting the ISCs is critical for IBD treatment. Here, we found that in the dextran sulfate sodium (DSS)-induced ulcerative colitis mice model, colon epithelium and Lgr5^+^ intestinal stem cells (ISCs) renew quickly during the first 3 days. We also found that during this renewing period, SMAD4 and bone morphogenetic protein 4 (BMP4) expression were significantly upregulated. An extra BMP4 treatment could preserve the Lgr5^+^ ISCs and the colon epithelium turnover, and could significantly decrease colon mucosal damage. Moreover, we found that BMP4 regulated ID3 expression in the colon epithelium. Depletion of ID3 could significantly reduce the epithelium renewal and ratio of Lgr5^+^ ISCs at the base of crypts. In conclusion, the present study showed that BMP4 could maintain epithelium cellular proliferation and the ISCs function through ID3 in mice with DSS-induced colitis. The administration of exogenous BMP4 supplement could alleviate DSS-induced colitis by restoring epithelium cellular proliferation and ISC function, suggesting the possible therapeutic function of BMP4 for ulcerative colitis.

## Introduction

The gastrointestinal tract is the key organ of metabolism in human beings. The single layer of the epithelium, which lies on the surface of the gastrointestinal tract, plays an important role in digestion, absorption, and pathogen defense. The histology of intestinal epithelium is in the form of crypt-villus-like structures. The intestinal stem cells (ISCs), which are localized at the base of the crypts, play a critical role in the fast turnover of the intestinal epithelium and gradient differentiation (Wang and Chen, 2018). Dysfunction of the epithelium turnover and ISCs were significant in the late stage of ulcerative colitis (UC), with the character of epithelium damage and inflammatory infiltration (Binienda et al., 2020). However, intestinal epithelial and ISC change in the early stage of UC remains unknown. Therefore, identifying the function and mechanism of early intestinal epithelial cell and ISC changes in UC is of significant importance in understanding the mechanism of the pathology of UC.

Multiple signaling pathways have been found to participate in epithelial cell proliferation and ISC differentiation. Evidence has been found that bone morphogenetic proteins (BMPs) play an important role in maintaining ISCs niche (McCarthy et al., 2020). Besides, BMPs are expressed in gastrointestinal mesenchymal tissue and have been found to regulate the proliferation of epithelial cells and ISC differentiation (Ji et al., 2017). Evidence has also been found, showing that activating gastric epithelium BMP signaling can reduce inflammation and dysplastic changes in the gastric mucosa (Takabayashi et al., 2014). Our previous study showed that mesenchymal stem cell-derived BMPs can exert anti-inflammatory effects and restore salivary gland epithelial secretion (Hu et al., 2020). However, BMP signaling expression during ulcerative colitis and its function on epithelial cells and ISCs remains unclear.

The present study aimed to investigate the intestinal epithelium turnover, ISC maintenance, and protein expression during the colonic inflammatory response in DSS-induced experimental colitis. The effects of BMP4 on the symptoms of colitis and intestinal epithelium proliferation and ISC maintenance in DSS-induced colitis were also investigated. Finally, this study elucidates the underlying mechanisms of BMP4 regulation on ISCs. Our study demonstrated the function and mechanism of BMP4 in the pathology of the DSS-induced colitis mouse model and suggested the therapeutic potential of BMP4 for the treatment of ulcerative colitis and other mucosal diseases.

## Materials and Methods

### Mice

C57BL/6 (6- to 8-week age, male) mice were obtained from Jackson Laboratories. *Id3-/-* mice (on C57BL/6 mice background) came from the National Institutes of Health (NIH) and were bred and maintained under specific pathogen-free conditions in the animal facility of the Capital Medical University. The animal experiments were approved by the Committee of the Capital Medical University (AEEI-2015-080).

### Induction of Colitis and Bone Morphogenetic Protein 4 Recombinant Protein and Anti-bone Morphogenetic Protein Treatment

To induce colitis, the mice were administered with 3% (w/v) dextran sulfate sodium (DSS; MP Biomedicals) in their drinking water for seven consecutive days. The mice were divided into three groups, and the mice in each group were administered with saline (100 μl), murine BMP4 recombinant protein (5 mg/kg, PerproTech, 315-27), or mouse BMP4 antibody (5 g/kg, R&D, MAB50201) by intraperitoneal injection on the third day of DSS treatment. The mice were observed daily for weight, morbidity, stool consistency, and blood in the feces and at the anus. On days 0, 3, 5, and 7, the mice were euthanized and rapidly dissected. The entire colon was quickly removed, and colonic length was measured.

### Liquid Chromatography Tandem Mass Spectrometry for Plasma Proteomics

The preparation of a protein sample was performed as the method previously described (León et al., 2013). Data acquisition was performed with a Triple-TOF 6600 mass spectrometer (Sciex, United States) fitted with a Nanospray III source (Sciex, United States) (Onder et al., 2018). The ion spray voltage was 2,300 V, declustering potential was 80 V, curtain gas was 35 psi, nebulizer gas was 5 psi, and interface heater temperature was 150°C. Peptides were introduced into the mass spectrometer using Nona 415 liquid chromatography (Sciex, United States). Chromatography solvents were water/acetonitrile/formic acid (A 98/2/0.1%, B 2/98/0.1%). Two-microliter samples were injected onto a C18 desalted column (3 μm, 120 Å, 350 μm ^∗^ 0.5 mm), and separated onto a C18 analysis column (3 μm, 120 Å, 75 μm ^∗^ 150 mm) with a gradient from 5 to 16% buffer B in 25 min, from 16 to 26% buffer B in the next 20 min, from 26 to 40% buffer B in the next 3 min, from 40 to 80% buffer B in the next 5 min, and 80 to 5% buffer B in the next 7 min, over 60 min at a flow rate of 0.6 μl/min. Peptides present in the data were identified by matching with the UniProt *Macaca mulatta tcheliensis* databases, and corresponding proteins were identified. The Paragon algorithm in Protein Pilot v 5.0 (SCIEX) was used to search the databases. The raw files were analyzed using MaxQuant software (Computational Systems Biochemistry). Proteins with missing values <50% were filled using k nearest neighbors, followed by normalization using the edgeR R package (version 3.28.1). Proteins with *p*-values <0.05 and fold-change ratios >1.5 were considered significant. Annotation and functional enrichment were performed using the Metascape database^1^ and the Ingenuity Pathway Analysis (IPA, QIAGEN).

### Reverse Transcriptase-Polymerase Chain Reaction and Real-Time Reverse Transcriptase-Polymerase Chain

The colon samples were homogenized with Trizol reagent, and total RNA was extracted from DPSCs with the RNAprep pure Cell/Bacteria Kit (TIANGEN, Beijing, China). Then cDNA was synthesized from 2 μg of prepared RNA with FastQuant RT Kit, complying with the protocol of the manufacturer (TIANGEN, Beijing, China). Real-time PCR reactions were performed with the SuperReal PreMix Plus SYBR Green PCR kit (TIANGEN, Beijing, China) and a CFX96 Touch Real-Time PCR Detection System (Bio-Rad, Hercules, CA, United States). The primers of specific genes are shown in Table 1.

**TABLE 1:** The primers of specific genes.

**Gene**	**Primer (5′ to 3′)**	
BMP2	Forward	TTCCATCACGAAGAAGCCGT
	Reverse	GAAACTCGTCACTGGGGACA
BMP4	Forward	GCCTTGTTTTCTGTCAAGACACC
	Reverse	TCTTCCCGGTCTCAGGTATCA
BMP7	Forward	CAGCCAGAATCGCTCCAAGA
	Reverse	TGCAATGATCCAGTCCTGCC
Hadh	Forward	TCGTGAACCGACTCTTGGTG
	Reverse	ATTTCATGCCACCCGTCCAA
Ifi204	Forward	AGCTGATTCTGGATTGGGCA
	Reverse	CAGTGATGTTTCTCCTGTTACTTCT
Srpk1	Forward	ACACGGCAGTATCGGTCTTT
	Reverse	AGGGCGATGTGGTCTTCATCT
Smad4	Forward	TAATCGCGCATCAACGGAGA
	Reverse	TGTGAACTGGCCTTGTGGAA
GAPDH	Forward	TCAGGAGAGTGTTTCCTCGTC
	Reverse	CCGTTGAATTTGCCGTGAGT

### Histology

Segments of the colon taken for histopathological analysis were fixed overnight in 4% paraformaldehyde, embedded in paraffin, and 4-μm sections were made. H&E-stained colon sections were used and scored for inflammation and epithelial damage. Disease activity index (DAI) was scored according to the average of three parameters: stool consistency, fecal blood, and percentage weight loss (Chen et al., 2019). The degree of inflammation and epithelial damage was scored with an average of two parameters including immune cell infiltration and intestinal architecture (Erben et al., 2014). The mucosal damage caused by mucositis was assessed by six different parameters: reduction of the intestinal crypts and villi; disruptions and abscess formation in the crypts; thickening of the outer muscle layer; integrity of the epithelium and muscular layer; inflammatory cell infiltration; and vacuolization and edema. The scores for each parameter range from 0 to 3 (0 = normal; 1 = mild; 2 = moderate; 3 = severe).

### Immunohistochemistry and Immunofluorescence

Paraffin-embedded tissue sections (5-μm thick) were deparaffinized using a series of xylene baths and rehydrated using an alcohol and water gradient. After rehydration, the slides were subjected to antigen retrieval in boiling sodium citrate buffer solution (pH 6.0) for 10 min. The slides were allowed to cool at room temperature for 1 h before being blocked for 20 min in blocking buffer (5% bovine serum albumin and 0.01% Triton X-100 in PBS; Sigma-Aldrich, St. Louis, MO, United States). The slides were then incubated with anti-BMP4 antibody (Abcam, ab39973, Cambridge, United Kingdom, 1:1,000 dilution), anti-Smad4 antibody (Abcam, ab40759, Cambridge, United Kingdom, 1:200), anti-Ki67 (Abcam, ab15580, Cambridge, United Kingdom, 1:1,000 dilution), anti-Lgr5 (Invitrogen, MA5-25644, 1:100), and anti-ID3 (Abcam, ab41834, Cambridge, United Kingdom, 1:200), overnight at 37°C. After incubation, the slides were washed in PBS for 5 min. Mouse anti-rabbit IgG-HRP (sc-2357, Santa Cruz, TX, United States, 1:500) were added, and a DAB kit was used for detection. For immunofluorescence staining, the slides were incubated for 1 h, at room temperature, with Alexa Fluor 594 Goat Anti-Rabbit IgG (Life Technologies, United States, 1:500) to detect the primary antibodies. The nuclei were also counterstained with DAPI (Invitrogen, Carlsbad, CA, United States). Immunohistochemical images were acquired using a Leica DM 4000 microscope (Leica, Germany), and analysis was performed using ImageJ. Immunohistochemical images were acquired using a Leica DM 4000 microscope (Leica, Germany), while immunofluorescent images were recorded using a Leica TCS SP5 confocal microscope (Leica, Germany). For quantifying fluorescence images, the mean cellular pixel values of all detected cells were normalized per channel by subtracting the lowest detected mean cellular pixel value. Cells with a normalized mean cellular pixel value above a set threshold per channel were counted as positive. To quantitate the number of Lgr5, Ki67, or ID3-positive cells per crypt, the well-oriented crypts were captured, and the total number of cells was quantitated by scoring the number of nuclei that stained positive for DAPI. The cells exhibiting bright fluorescence were counted as the positive cell.

### Statistical Analysis

Data are expressed as mean ± SD. The data were analyzed by one-way analysis of variance and Student *t*-test. Multiple comparisons between groups were performed using Bonferroni post-tests. A value of *p* < 0.05 was considered statistically significant. Statistical analyses were performed using GraphPad Prism software 8.0 (GraphPad Software, La Jolla, CA, United States).

## Results

### The Symptoms of Dextran Sulfate Sodium-Induced Colitis Were Significantly Increased at 3 Days

**FIGURE 1:**
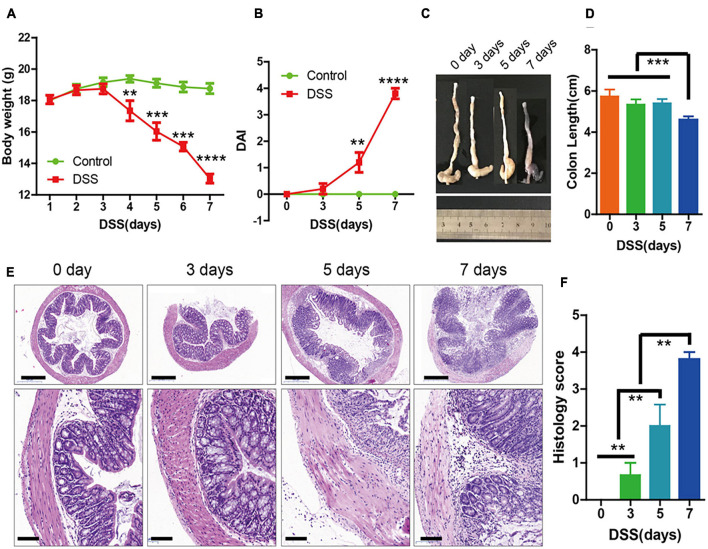
The symptoms of dextran sulfate sodium (DSS)-induced colitis were significantly increased at 3 days. **(A)** Body weight in the control group and DSS-induced group (*n* = 10 per group) for 0 to 7 days. **(B)** Disease activity index (DAI) of each group (*n* = 10) for 0, 3, 5, and 7 days. **(C,D)** Colon lengths (*n* = 10). **(E)** Sections of colon tissues stained with hematoxylin and eosin (H&E) and **(F)** histology scores from the two groups. Data represent mean ± SD with three independent experiments. One-way ANOVA and Bonferroni tests for multiple testing were applied in all cases. **p < 0.01, ***p < 0.001, ****p < 0.0001. Scale bar = 500 and 100 μm.

Oral administration of 3% DSS for 7 days was used to induce acute colitis in mice. The body weight of the mice decreased dramatically after day 5 (Figure 1A), and there was also an increase in disease activity index (Figure 1B), which was evaluated based on body weight loss and bloody diarrhea/loose feces. The length of the colons were significantly shorter at day 7 (Figures 1C,D). In addition, hematoxylin and eosin (H&E) staining showed that mucosal ulceration of the colon and transmural inflammation was significantly increased at day 5 (Figures 1E,F). These results indicated that the symptoms of DSS-induced colitis were significantly increased 5 days after DSS administration.

### High Intestinal Epithelium Proliferation and Lgr5^+^ Intestinal Stem Cells Are Maintained in the Early 3 Days of Dextran Sulfate Sodium-Induced Colitis

**FIGURE 2:**
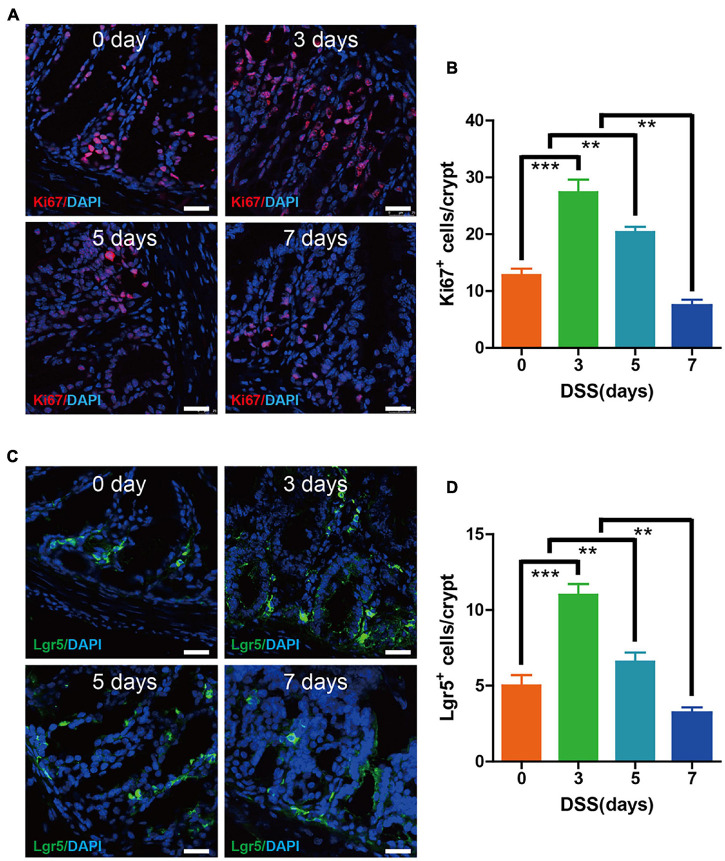
Epithelium cell proliferation and Lgr5^+^ stem cell maintenance were preserved during the early 3 days of DSS colitis. **(A)** Immunofluorescence staining for Ki67 in colon tissue from mice treated with DSS at 0, 3, 5, and 7 days (red: Ki67, blue: DAPI). **(B)** Quantification of Ki67-positive cells per crypt (*n* = 5). **(C)** Immunofluorescence staining for Lgr5 in colon tissue from mice treated with DSS at 0, 3, 5, and 7 days (green: Lgr5, blue: DAPI). **(D)** Quantification of Lgr5-positive cells per crypt (*n* = 5). Data represent mean ± SD with three independent experiments. One-way ANOVA and Bonferroni test for multiple testing was applied in all cases. **p < 0.01, ***p < 0.001. Scale bar = 25 μm.

Since the intestinal epithelium proliferation and Lgr5^+^ stem cells were essential for maintaining the epithelium turnover upon DSS colitis, we investigated at days 0, 3, 5, and 7, respectively. Immunofluorescence labeling for Ki67 indicated a significantly higher amount of proliferating cells on colon epithelium at day 3 when compared with day 0. The proliferation rate of epithelium was still high on day 5, despite some alleviation when compared with the peak rate on day 3. However, the proliferation rate of epithelium took a sharp decrease at day 7 when compared with other groups (Figures 2A,B). Immunofluorescence labeling for Lgr5 showed that the Lgr5^+^ stem cells in epithelium cells were upregulated at days 3 and 5, with the highest rate occurring on day 3, while steadily declining starting on day 7 (Figures 2C,D). These results showed that during the first 3 days of DSS-induced colitis, we witnessed the enhanced epithelium proliferation and Lgr5^+^ stem cells, which may have been due to tissu self-repair.

### SMAD4 and Bone Morphogenetic Protein 4 in the Colon Were Upregulated in the First 3 Days of Dextran Sulfate Sodium-Induced Colitis

**FIGURE 3:**
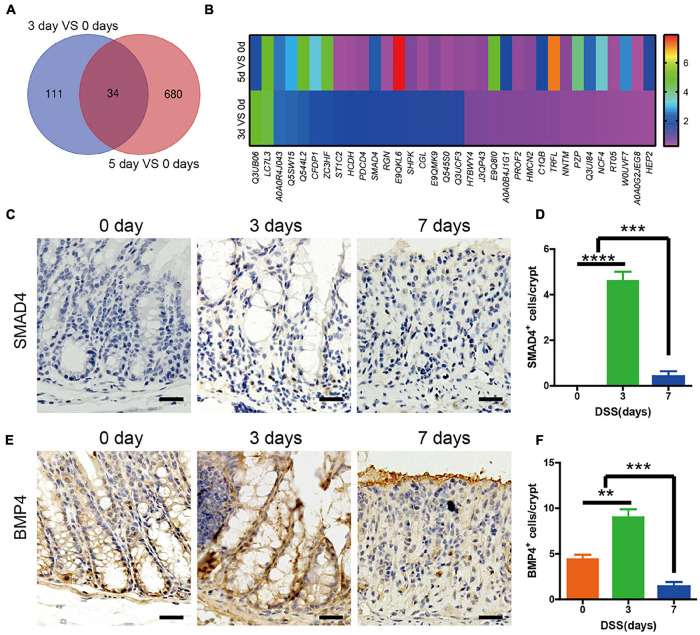
SMAD4 and bone morphogenetic protein 4 (BMP4) expression in the colon of DSS-induced colitis mice. **(A)** Venn diagram showing the overlap colon protein between DSS-induced colitis on day 3 compared with day 0 and day 5 compared with day 0 (*n* = 3). **(B)** Heatmap showing the expression levels of overlap colon protein between DSS-induced colitis on day 3 compared with day 0 and day 5 compared with day 0. **(C)** Immunohistochemistry staining of SMAD4 expression in DSS mice colon at days 0, 3, and 7. **(D)** Quantification of SMAD4-positive cells per crypt (*n* = 5). **(E)** Immunohistochemistry staining of BMP4 expression in DSS mice colon at days 0, 3, and 7. **(F)** Quantification of BMP4-positive cells per crypt (*n* = 5). Data represent mean ± SD with three independent experiments. One-way ANOVA and Bonferroni test for multiple testing was applied in all cases. **p < 0.01, ***p < 0.001, ****p < 0.0001. Scale bar = 100 μm.

In order to detect the significant amount of signaling related to the upregulated intestinal epithelium, proliferation and Lgr5^+^ ISCs were maintained in the first 3 days. The mice colons were acquired on days 0, 3, and 5 of DSS treatment, and quantitative assessment of protein was performed. Our data showed that when compared with day 0, there were 145 differentially expressed proteins identified in the colon of mice sacrificed on day 3, with 79 upregulated and 66 downregulated. In the colon of mice sacrificed on day 5, there were 714 differentially expressed proteins identified, with 345 upregulated and 369 downregulated. Then we found that 34 proteins were expressed on both day 3 and day 5, when compared with day 0 (Figure 3A). Among the 34 expressed proteins, there were 10 proteins, including SMAD4, that were upregulated both on day 3 and day 5, when compared with day 0 (Figure 3B). The data were verified by rtPCR (Supplementary Figure 1A). KEGG analysis showed the differentially expressed proteins related to pathways like complement and coagulation cascades, infection, or metabolism. Among them, SMAD4-related Hippo signaling would be directly regulating the intestinal epithelium cells (Supplementary Figure 1B). In order to detect which signaling induced the upregulated SAMD4, we investigated the BMP2, 4, 7 (the main BMPs in intestinal tissue) expression in colon by rtPCR. The data showed that BMP4 was the most significantly upregulated (Supplementary Figure 1C). Immunohistochemistry staining of SMAD4 and BMP4 was performed in DSS mice colon at days 0, 3, and 7. The results showed that there were almost no expression of SMAD4 at day 0, until it was increased at day 3, and eventually decreased at day 7 (Figures 3C,D). At day 0, there was BMP4 expression at the base of the crypt; the entire crypt was populated on day 5 and diminished on day 7 (Figures 3E,F).

### Bone Morphogenetic Protein 4 Recombinant Protein Ameliorated Dextran Sulfate Sodium-Induced Colitis

**FIGURE 4:**
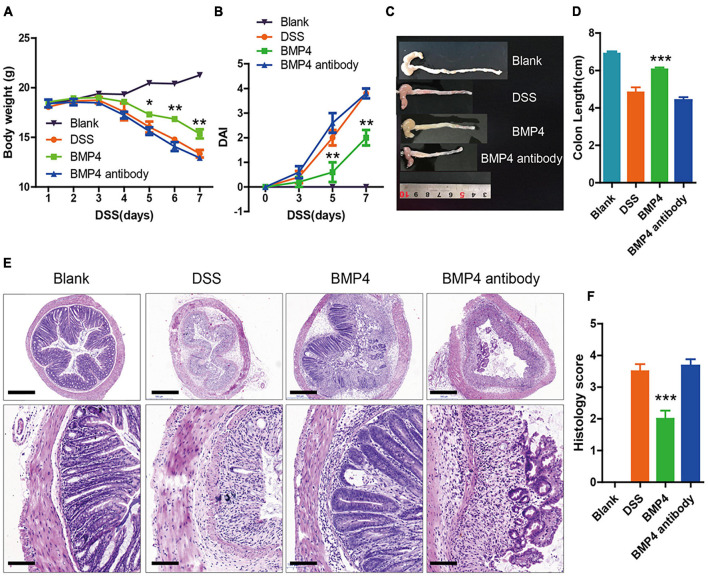
BMP4 alleviates DSS-induced colitis. **(A,B)** Body weight and DAI score of mice treated with DSS. At day 3, the control group was administered with saline, the BMP4 group was administered with BMP4, and the BMP4 antibody group was administrated with anti-BMP4 antibody. **(C,D)** Colon length from different groups. **(E)** Sections of colon tissues stained with H&E and **(F)** histology scores from different groups. *n* = 10 (control), *n* = 10 (BMP4), *n* = 10 (anti-BMP4). Data represent mean ± SD with three independent experiments. One-way ANOVA and Bonferroni tests for multiple testing were applied in all cases. *p < 0.05, **p < 0.01, ***p < 0.001. Scale bar = 500 and 100 μm.

In order to explore the function of exogenous BMP4 supplementation on the disease symptoms of DSS-induced colitis *in vivo*, we used the mice of DSS-induced colitis to investigate the influence of BMP4 recombinant protein or anti-BMP4 antibody treatment on epithelial cell proliferation and ISC maintenance ability. Our data showed that the DSS-induced mice treated by BMP4 on day 3 could increase body weight (Figure 4A), reduce disease activity index scores (DAI) (Figure 4B), resist changes in colon length (Figures 4C,D), and reduce histology score (Figures 4E,F), when compared with DSS-induced mice treated by saline. However, DSS-treated mice treated with anti-BMP4 antibody on day 3 showed no effects on DSS-induced colitis compared with DSS-treated mice treated by saline, and both groups had similar DAI scores, colon length, and histology score. These data demonstrated that BMP4 supplementation could play a protective role in DSS-induced colitis.

### Bone Morphogenetic Protein 4 Recombinant Protein Increase Colon Epithelium Proliferation and Lgr5^+^ Intestinal Stem Cells Are Maintained in Dextran Sulfate Sodium-Induced Colitis

**FIGURE 5:**
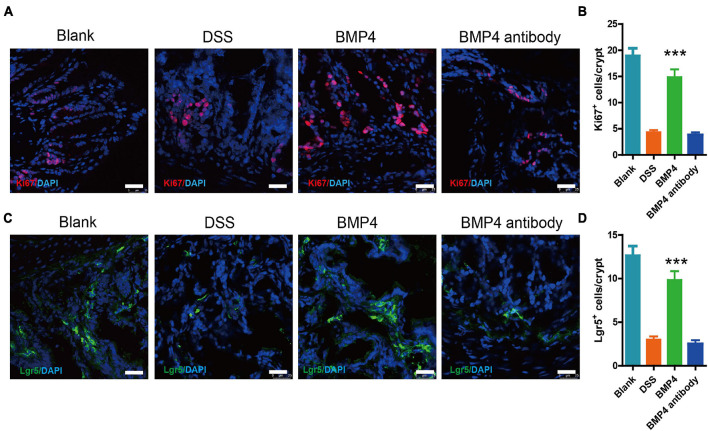
BMP4 maintains epithelium cell proliferation and Lgr5^+^ ISCs following DSS-induced injury. **(A)** Immunofluorescence staining for Ki67 in colon tissue from DSS-induced mice treated with saline, BMP4, or anti-BMP4 antibody (red: Ki67, blue: DAPI). **(B)** Quantification of Ki67-positive cells per crypt (*n* = 5). **(C)** Immunofluorescence staining for Lgr5 in colon tissue from DSS-induced mice treated with saline, BMP4, or anti-BMP4 antibody (green: Lgr5, blue: DAPI). **(D)** Quantification of Lgr5-positive cells per crypt (*n* = 5). Data represent mean ± SD with three independent experiments. One-way ANOVA and Bonferroni tests for multiple testing were applied in all cases. ***p < 0.001. Scale bar = 25 μm.

We further checked the colon epithelium proliferation and Lgr5^+^ ISC maintenance ability in DSS-induced colitis treated with BMP4 recombinant protein and antibody at 7 days. Immunofluorescence labeling for Ki67 showed that DSS-induced colitis treated with BMP4 has a significantly enhanced proliferating epithelium cell layer when compared with DSS-induced colitis that is treated with antibody (Figures 5A,B). Immunofluorescence labeling for Lgr5 showed that the Lgr5^+^ cells were upregulated in the DSS-induced colitis group treated with BMP4 (Figures 5C,D). As fibrosis is another important pathological character of colitis, we detect the colon by Masson’s trichrome staining. Our data showed that the collagen deposition was high in the colon of colitis, but with no significant difference between BMP4 recombinant protein and BMP4 antibody treatment (Supplementary Figure 2A). These results showed that BMP4 plays a key function in epithelium proliferation and Lgr5^+^ stem cell maintenance ability.

### Bone Morphogenetic Protein 4 Ameliorated Dextran Sulfate Sodium-Induced Colitis by Target ID3

**FIGURE 6:**
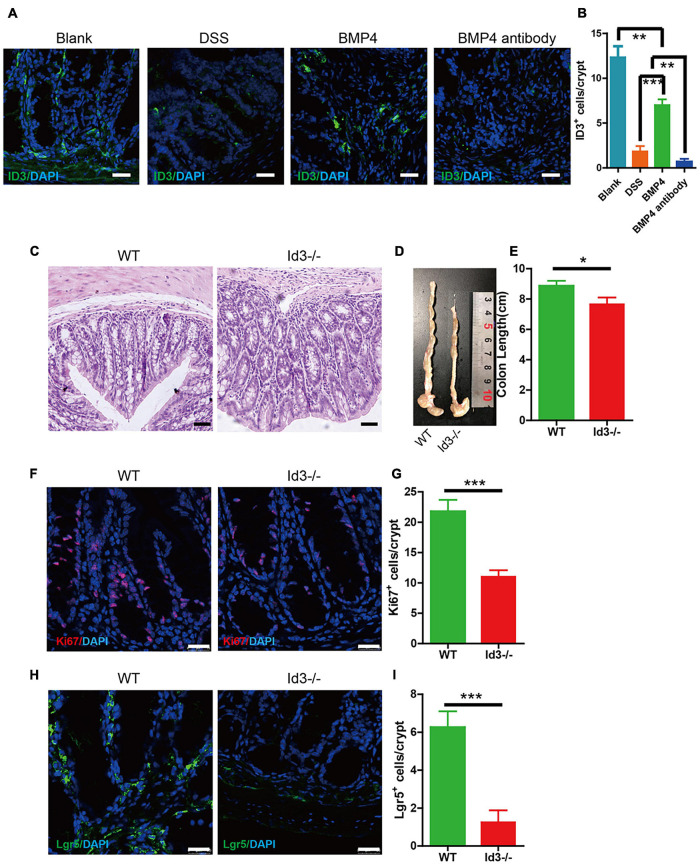
BMP4 ameliorated DSS-induced colitis by target ID3. **(A)** Immunofluorescence staining for ID3 expression in blank mice, DSS-induced mice treated with saline, BMP4, or anti-BMP4 antibody (green: ID3, blue: DAPI). **(B)** Quantification of ID3-positive cells per crypt from the colon. **(C)** Sections of colon tissues stained with H&E from *Id3^–/–^* aged mice and wild-type aged mice. **(D,E)** Colon length from *Id3^–/–^* aged mice and wild-type aged mice. **(F)** Immunofluorescence staining of Ki67 in colon tissue from *Id3^–/–^* aged mice and wild-type aged mice. **(G)** Quantification of Ki67-positive cells per crypt from *Id3^–/–^* aged mice and wild-type aged mice. **(H)** Immunofluorescence labeling shows the Lgr5-positive cells in tissue from *Id3^–/–^* aged mice and wild-type aged mice. **(I)** Quantification of Lgr5-positive cells per crypt from *Id3^–/–^* aged mice and wild-type aged mice. Data represent mean ± SD; one-way ANOVA and Bonferroni tests for multiple testing. Student *t*-test was used for data analyses from *Id3^–/–^* aged mice and wild-type aged mice. *p < 0.05, **p < 0.01, ***p < 0.001. Scale bar = 25 μm.

ID3 is the downstream target gene of BMP4. We investigated the expression of ID3 in DSS-induced colitis mice using BMP4 recombinant protein and anti-BMP4 treatment. The results showed that BMP4 could upregulate ID3 expression in the bottom of the crypt compared with DSS mice, which would be abolished by anti-BMP4 (Figures 6A,B). To further explore the function of ID3 on epithelial cell proliferation and ISC maintenance ability, we generated Id3 knockout (*Id3^–/–^*) mice on C57BL/6 mice background and bred until 1 year of age. The H&E staining showed that the average crypt length of colon tissue from *Id3^–/–^* mice was significantly lower than that of wild-type mice (Figure 6C), and also showed reduced colon length (Figures 6D,E). Immunofluorescence labeling of Ki67 showed that the Ki67-positive cells at the bottom of the crypt almost disappeared from the tissue of *Id3^–/–^* aged mice when compared with the levels found in wild-type aged mice (Figures 6F,G). Lgr5-positive cells also almost disappeared in the tissue from Id3−/− aged mice when compared with the levels found in wild-type aged mice (Figures 6H,I). Masson’s trichrome staining showed no significantly difference of collagen deposition between *Id3^–/–^* aged mice and wild-type aged mice (Supplementary Figure 2B). This data demonstrated that ID3 was the key gene for intestinal epithelium proliferation and Lgr5^+^ ISC maintenance ability.

## Discussion

In the present study, our results showed that upregulated SMAD4 and BMP4 expression was related to a more rapid colon epithelium renewal and Lgr5^+^ ISC maintenance ability during the first 3 days of DSS induced experimental colitis in mice. BMP4 recombinant protein treatment could preserve Lgr5^+^ ISCs, colon epithelium turnover, and could even alleviate the symptoms of colitis in experimental mice by targeting ID3. Our results demonstrated that BMP4 has a therapeutic potential for treating inflammatory conditions of the colon by targeting ID3.

BMP4 belongs to the transforming growth factor-β (TGF-β) superfamily. After binding to type I and type II receptors, BMP4 would activate R-SMADs (SMAD1, 5, and 8), then form complexes with Co-SMAD (SMAD4). This SMAD complex translocates to the nucleus to directly regulate the transcription of downstream target genes, including ID3. The location of BMP signaling in the intestines is position dependent. For example, evidence has been found that BMP2 or BMP4 are mainly expressed in intravillus and intercrypt mesenchymal cells, while the BMP antagonists (Noggin and Gremlin 1) are mainly expressed in mesenchymal cells beneath the crypt. This location-specific expression pattern generates the gradient of BMP activity along the crypt–villus axis, with the lowest one at the bottom of the crypt (Wang and Chen, 2018). Many previous studies showed that BMP4 is mainly expressed in the intravillus and intercrypt mesenchymal cells, and our result showed that BMP4 could also be expressed in the epithelium of the bottom of crypt. This is supported by a previous study, as it has been shown that epithelial BMP has significant regulatory effects on gastric physiology (Maloum et al., 2011). Whether the BMP4 is secreted by the epithelial cell itself, or is *trans-*located from mesenchymal cells, remains unknown and needs to be investigated further.

It has been generally acknowledged that BMP signaling inhibits the hyperproliferation of the epithelium in normal intestinal function. Recent studies have shown that intestine regeneration would be impaired by epithelial overexpression of BMP4 (Koppens et al., 2021). Negative control of BMP signaling on intestinal stromal cells would enhance the proliferation and differentiation of ISCs (Yu et al., 2020). It also showed that BMP signaling directly suppresses the expression of ISC signature genes by targeting SMAD4. Specific deletion of Bmpr1a in Lgr5^+^ ISCs leads to stem cell hyperproliferation under homeostatic and injury-repair conditions (Qi et al., 2017). Emerging evidence suggests that BMP signaling generally inhibits disease progression of inflammatory bowel disease, including Crohn’s disease and UC (Zhang and Que, 2020). However, the function of BMP signaling on intestinal epithelium under the condition of injury and inflammation is different from the situation of normal intestinal epithelium. Administration of BMP4 seems to ameliorate colon damage through an anti-inflammatory response during bacterial infection (Takabayashi et al., 2014). It was found that the reduced expression of BMP4 and BMPR1A was co-related with colon injury and inflammation in DSS-induced colitis, and the colonic inflammation and damage could be enhanced through deletion of BMPR1A (Ji et al., 2017). Besides, the downexpression of SMAD4 is strongly associated with intestinal tumorigenesis in mice with inflammatory bowel disease, with the abnormal proliferation of intestinal epithelium (Alberici et al., 2006). The conflicting function of BMP signaling on the intestinal epithelium under normal conditions and inflammatory conditions revealed the complex function of BMPs. For example, evidence has shown that both epithelial and mesenchymal cells in the intestine can express BMP signals and receptors, suggesting that there is a bidirectional regulation between the mesenchyme and the epithelium (Li et al., 2007). In addition, BMP signaling can regulate the epithelium through cross-talk with other signaling pathways, such as the balance Wnt signaling-induced hyperproliferation of ISCs (Qi et al., 2017).

Thus, in order to confirm the function of BMP4 on intestinal epithelium, we dynamically detected the intestinal epithelium proliferation and Lgr5^+^ ISCs in mice with DSS-induced colitis. Our data showed that intestinal epithelium proliferation and Lgr5^+^ ISCs were increased during the first 3 days of colitis. This time point was not consistent with previous studies; this reason may be due to the different schemas of DSS treatment and mice strain (Harnack et al., 2019). Our results showed that BMP4 and SMAD4 were downregulated at the late stage of DSS-induced colitis, which is consistent with other studies we examined. Interestingly, we found that BMP4 and SMAD4 were upregulated at the early stage, which was co-related with the high epithelium proliferation and Lgr5^+^ ISCs. In addition, BMP4 treatment can alleviate the symptoms of DSS-induced colitis, although the disease symptoms were not significantly changed with BMP4-neutralized antibody treatment. This may be attributed to the fact that treatment was initiated on the third day, as this is when epithelium proliferation and Lgr5^+^ ISCs started to decline. The previous study had also showed that BMP4 can relieve *H. pylori*- or *H. felis*-induced mice intestinal inflammation, which was independent on dendritic cells or splenocytes (Takabayashi et al., 2014). The exact mechanism of this treatment remains unclear. Here, we found that BMP4 treatment could increase epithelium proliferation and Lgr5^+^ ISCs, which would shed light on the mechanism of the therapeutic function of BMP4.

The inhibitor of DNA-binding (ID) proteins is reported as the downstream gene of BMP (Hollnagel et al., 1999), and ID1 (Zhang et al., 2014), ID2 (Nigmatullina et al., 2017) and ID3 (Raab et al., 2020) were all functional marker for ISCs. Our previous study showed that ID3 negatively regulated BMP4 expression in mesenchymal stem cells and plays a function in salivary gland immunoregulation and epithelium cellular function restoration (Hu et al., 2020). Our results showed that BMP4 treatment could increase the ID3 expression. The ID3 knockout mice have system immune disorder, thus, is not suitable to evaluate the function for intestinal epithelium in DSS induced colitis model. In order to detect the function of ID3 on the intestinal epithelium, the aging model was used. The intestinal aging would result in decreased epithelium cell proliferation or Lgr5^+^ ISCs. We found that the colon in ID3 knockout aged mice has a decreased proliferation rate when compared with wild-type mice, especially at the bottom of the crypt. Researchers also found that ID3-null mice at 8 weeks of age carry a red fluorescent protein inserted in the first exon of ID3, resulting in the loss of expression of ID3 (Raab et al., 2020). They also found that they exhibit an increased number of cells expressing the ISC-specific marker (Raab et al., 2020), which was not consistent with our results. This may be due to the use of a different mouse model, as well as the age of the mice. The reason why the aged ID3 knockout mice would reduce the ISCs needs to be further investigated.

## Conclusion

This study showed that the upregulated expression of SMAD4 and BMP4 was related to rapid colon epithelium renewal and increased Lgr5^+^ ISC maintenance ability. BMP4 recombinant protein treatment could preserve Lgr5^+^ ISCs and colon epithelium turnover, and significantly decrease colon mucosal damage, which could be blocked by BMP4 antibody. Additionally, our results showed that BMP4 regulated cellular renewal and Lgr5^+^ ISCs by targeting ID3. Collectively, the results of this current study demonstrated that BMP4 has therapeutic potential for treating inflammatory conditions of the colon by targeting ID3.

## Data Availability Statement

The datasets presented in this study can be found in online repositories. The names of the repository/repositories and accession number(s) can be found below: ProteomeXchange Consortium via the PRIDE partner repository with the dataset identifier PXD026228.

## Ethics Statement

The animal study was reviewed and approved by the Committee of the Capital Medical University.

## Author Contributions

LH acquired and analyzed the data, and drafted and critically revised the manuscript. JX acquired and analyzed the data. XW analyzed the data. CZ and JW designed the study and drafted the manuscript. SW conceptualized and designed the study, interpreted the data, acquired funding, and critically revised the manuscript. All authors gave final approval and agreed to be accountable for all aspects of the work.

## Conflict of Interest

The authors declare that the research was conducted in the absence of any commercial or financial relationships that could be construed as a potential conflict of interest.

## Publisher’s Note

All claims expressed in this article are solely those of the authors and do not necessarily represent those of their affiliated organizations, or those of the publisher, the editors and the reviewers. Any product that may be evaluated in this article, or claim that may be made by its manufacturer, is not guaranteed or endorsed by the publisher.

## Footnotes

1
http://metascape.org

## Supplementary Material

Supplementary Figure 1 **(A)** rtPCR verifying the expression of Hadh, Ifi204, Srpk1, Smad4 in the colon of DSS-induced colitis at day 0, 3, 5. **(B)** KEGG analysis the differential protein related pathway. **(C)** rtPCR show the expression of BMP2,4,7 in the colon of DSS-induced colitis at day 0, 3, 5. Data represent mean ± SD; one-way ANOVA and Bonferroni test for multiple testing. ^∗∗^*p* < 0.01, ^∗∗∗^*p* < 0.001.

Supplementary Figure 2 **(A)** Masson’s trichrome staining showed the collagen deposition in each group, no significant difference between BMP4 recombinant protein and BMP4 antibody treatment. **(B)** Masson’s trichrome staining showed no significant difference of collagen deposition between *Id3*^–/–^ aged mice and wild type aged mice. Scale bar = 50 μm.
