# Supplementary figures and images for: Bone Morphogenetic Protein 4 Alleviates DSS-Induced Ulcerative Colitis Through Activating Intestinal Stem Cell by Target ID3
Source: Front Cell Dev Biol. 2021 Oct 6;9:700864. doi: 10.3389/fcell.2021.700864 (PMC8528200; doi:10.3389/fcell.2021.700864)

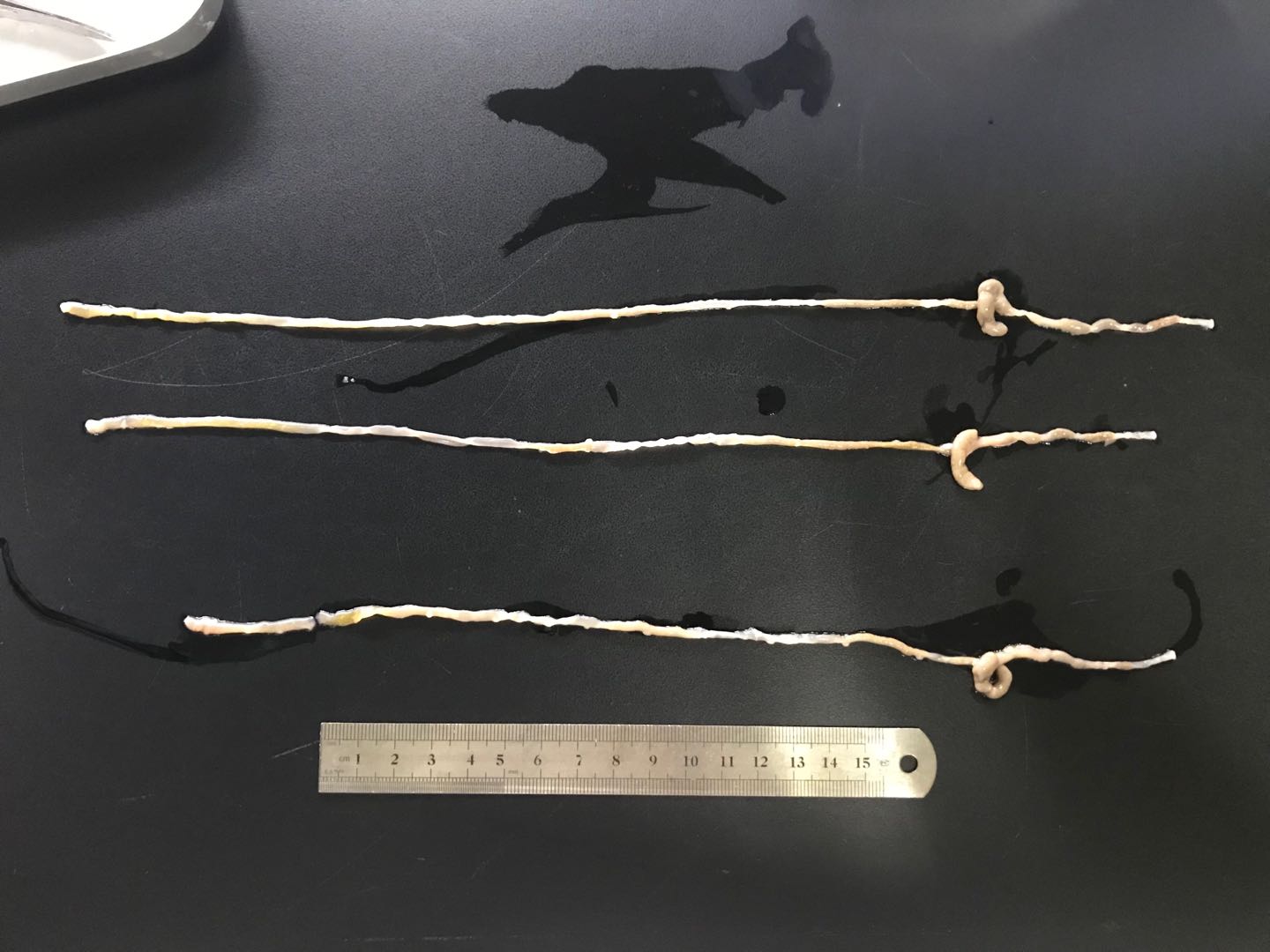

Supplement: Supplementary Figure 1 — (A) rtPCR verifying the expression of Hadh, Ifi204, Srpk1, Smad4 in the colon of DSS-induced colitis at day 0, 3, 5. (B) KEGG analysis the differential protein related pathway. (C) rtPCR show the expression of BMP2,4,7 in the colon of DSS-induced colitis at day 0, 3, 5. Data represent mean ± SD; one-way ANOVA and Bonferroni test for multiple testing. ∗∗p < 0.01, ∗∗∗p < 0.001. [file Data_Sheet_1.ZIP › fig1/0day.jpg]

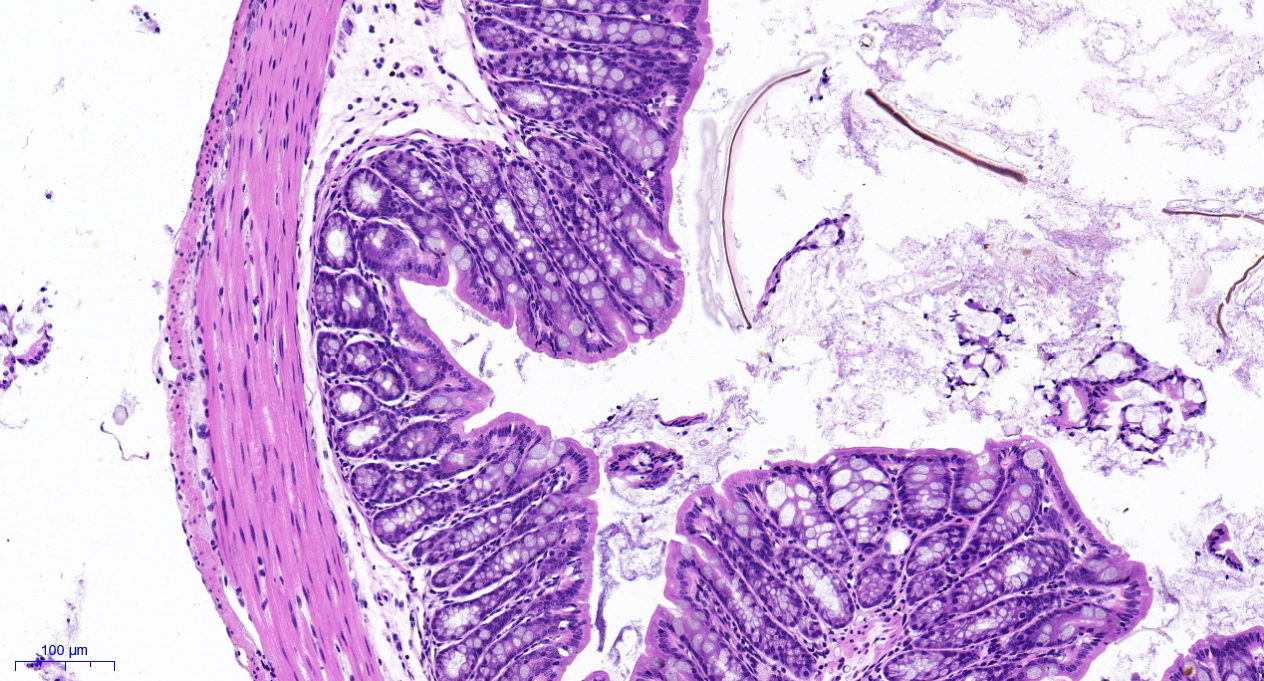

Supplement: Supplementary Figure 1 — (A) rtPCR verifying the expression of Hadh, Ifi204, Srpk1, Smad4 in the colon of DSS-induced colitis at day 0, 3, 5. (B) KEGG analysis the differential protein related pathway. (C) rtPCR show the expression of BMP2,4,7 in the colon of DSS-induced colitis at day 0, 3, 5. Data represent mean ± SD; one-way ANOVA and Bonferroni test for multiple testing. ∗∗p < 0.01, ∗∗∗p < 0.001. [file Data_Sheet_1.ZIP › fig1/0day_10.0x.jpg]

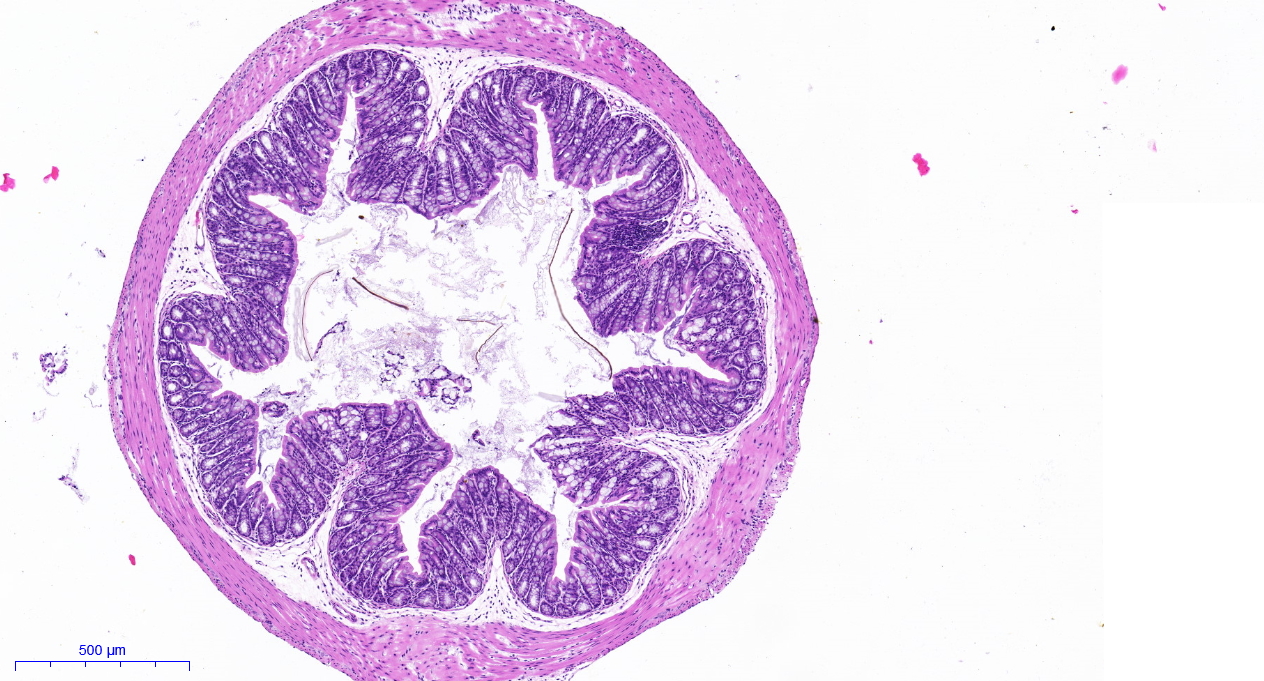

Supplement: Supplementary Figure 1 — (A) rtPCR verifying the expression of Hadh, Ifi204, Srpk1, Smad4 in the colon of DSS-induced colitis at day 0, 3, 5. (B) KEGG analysis the differential protein related pathway. (C) rtPCR show the expression of BMP2,4,7 in the colon of DSS-induced colitis at day 0, 3, 5. Data represent mean ± SD; one-way ANOVA and Bonferroni test for multiple testing. ∗∗p < 0.01, ∗∗∗p < 0.001. [file Data_Sheet_1.ZIP › fig1/0day_3.5x.jpg]

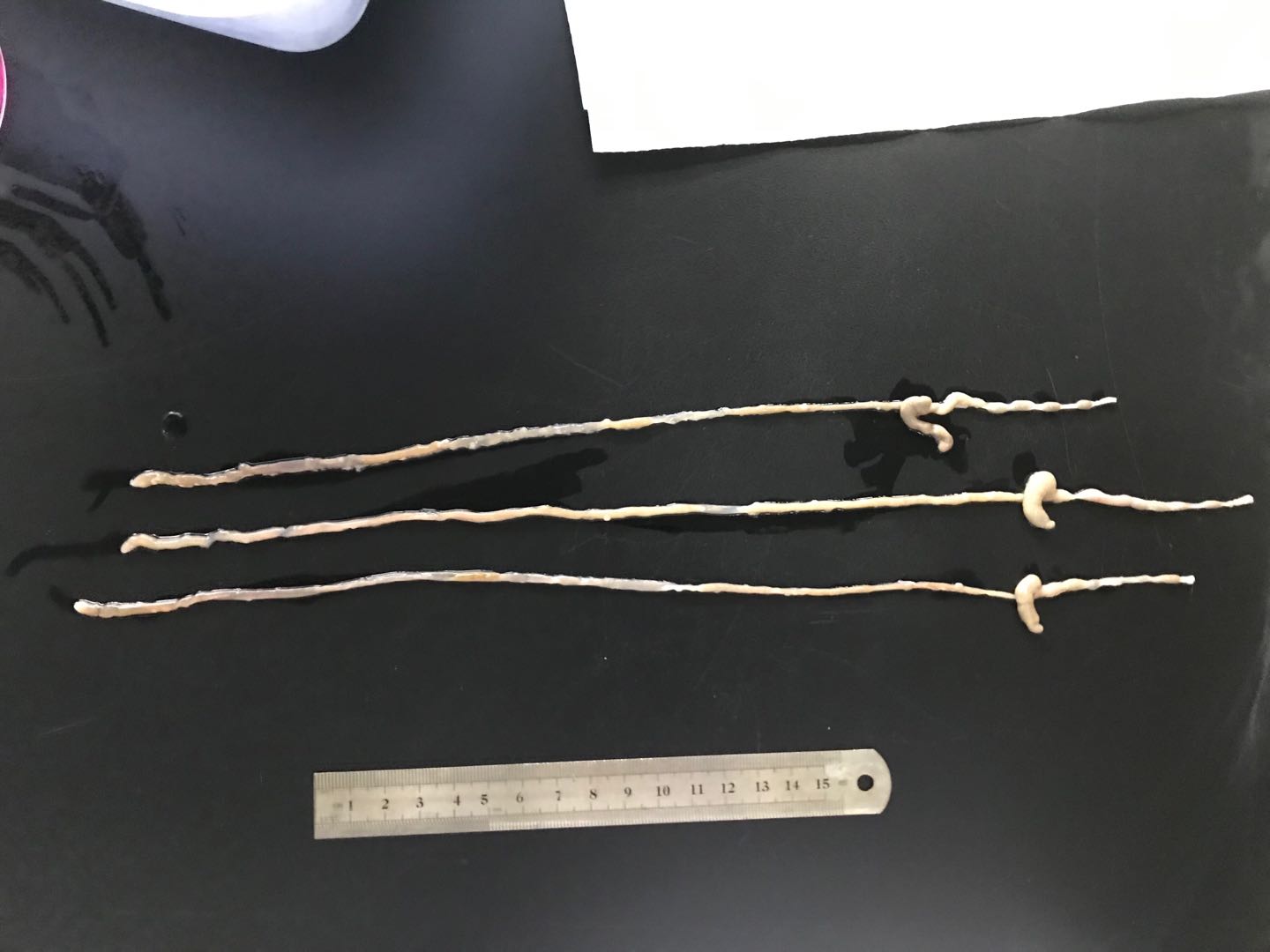

Supplement: Supplementary Figure 1 — (A) rtPCR verifying the expression of Hadh, Ifi204, Srpk1, Smad4 in the colon of DSS-induced colitis at day 0, 3, 5. (B) KEGG analysis the differential protein related pathway. (C) rtPCR show the expression of BMP2,4,7 in the colon of DSS-induced colitis at day 0, 3, 5. Data represent mean ± SD; one-way ANOVA and Bonferroni test for multiple testing. ∗∗p < 0.01, ∗∗∗p < 0.001. [file Data_Sheet_1.ZIP › fig1/3day.jpg]

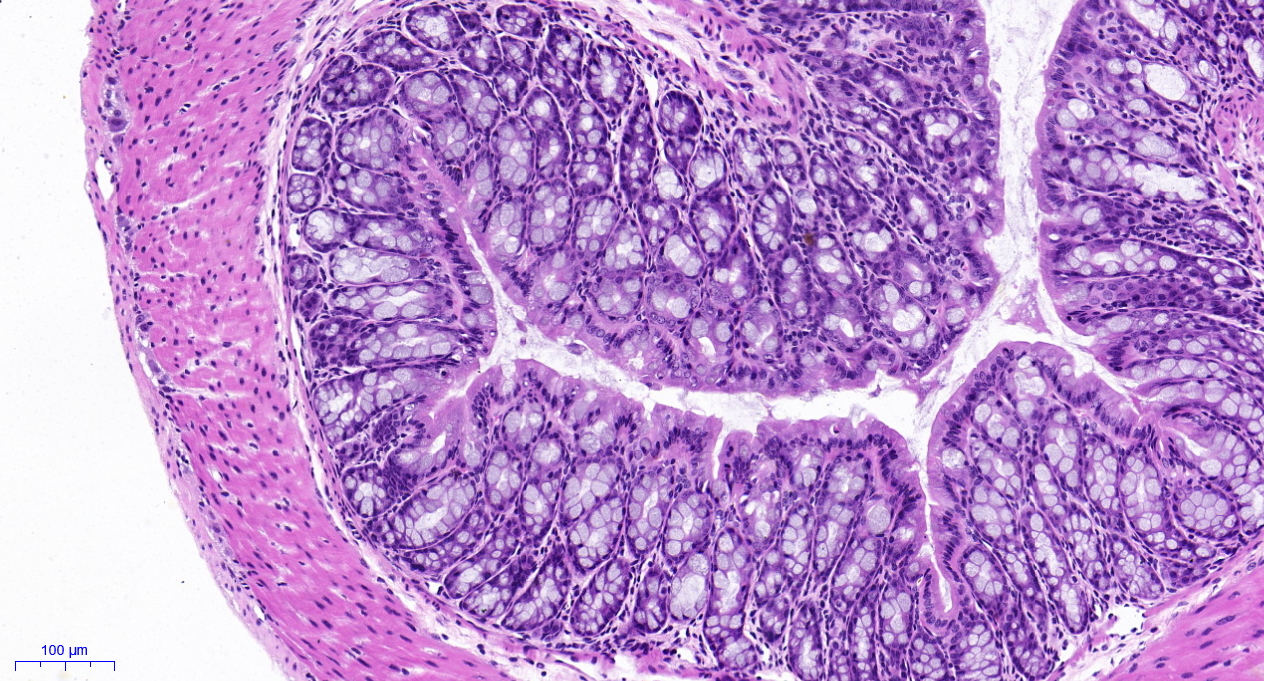

Supplement: Supplementary Figure 1 — (A) rtPCR verifying the expression of Hadh, Ifi204, Srpk1, Smad4 in the colon of DSS-induced colitis at day 0, 3, 5. (B) KEGG analysis the differential protein related pathway. (C) rtPCR show the expression of BMP2,4,7 in the colon of DSS-induced colitis at day 0, 3, 5. Data represent mean ± SD; one-way ANOVA and Bonferroni test for multiple testing. ∗∗p < 0.01, ∗∗∗p < 0.001. [file Data_Sheet_1.ZIP › fig1/3day_10.0x.jpg]

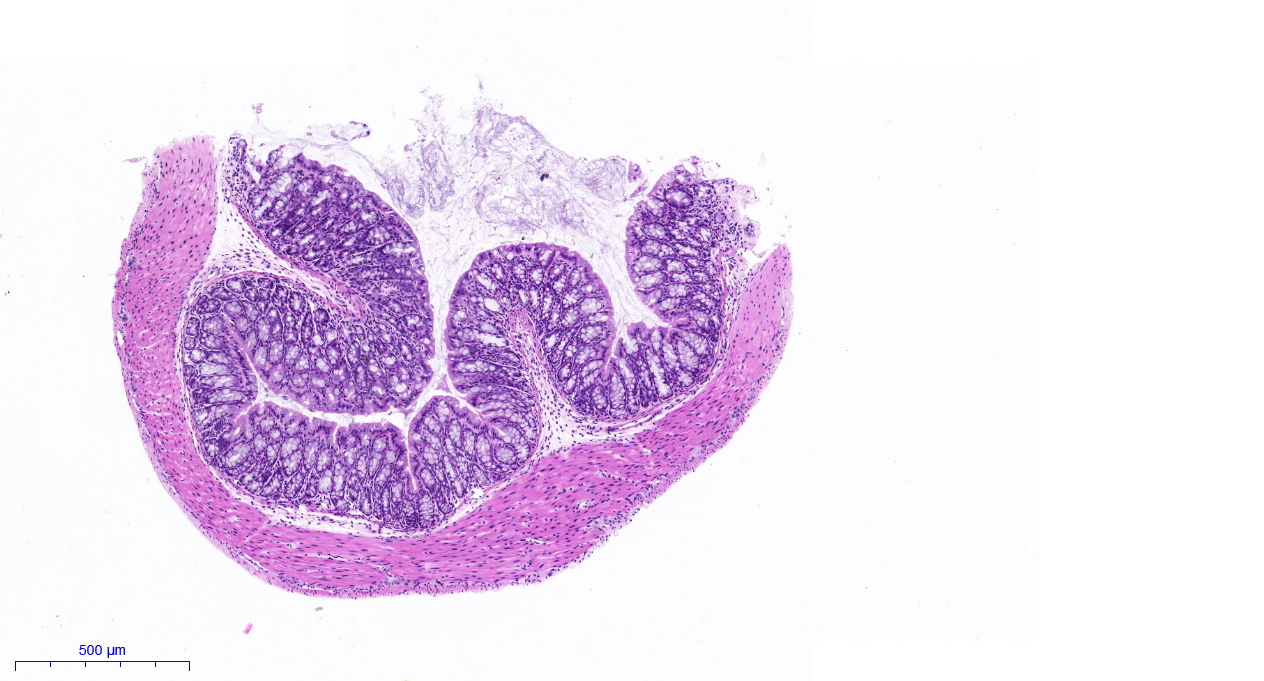

Supplement: Supplementary Figure 1 — (A) rtPCR verifying the expression of Hadh, Ifi204, Srpk1, Smad4 in the colon of DSS-induced colitis at day 0, 3, 5. (B) KEGG analysis the differential protein related pathway. (C) rtPCR show the expression of BMP2,4,7 in the colon of DSS-induced colitis at day 0, 3, 5. Data represent mean ± SD; one-way ANOVA and Bonferroni test for multiple testing. ∗∗p < 0.01, ∗∗∗p < 0.001. [file Data_Sheet_1.ZIP › fig1/3day_3.5x.jpg]

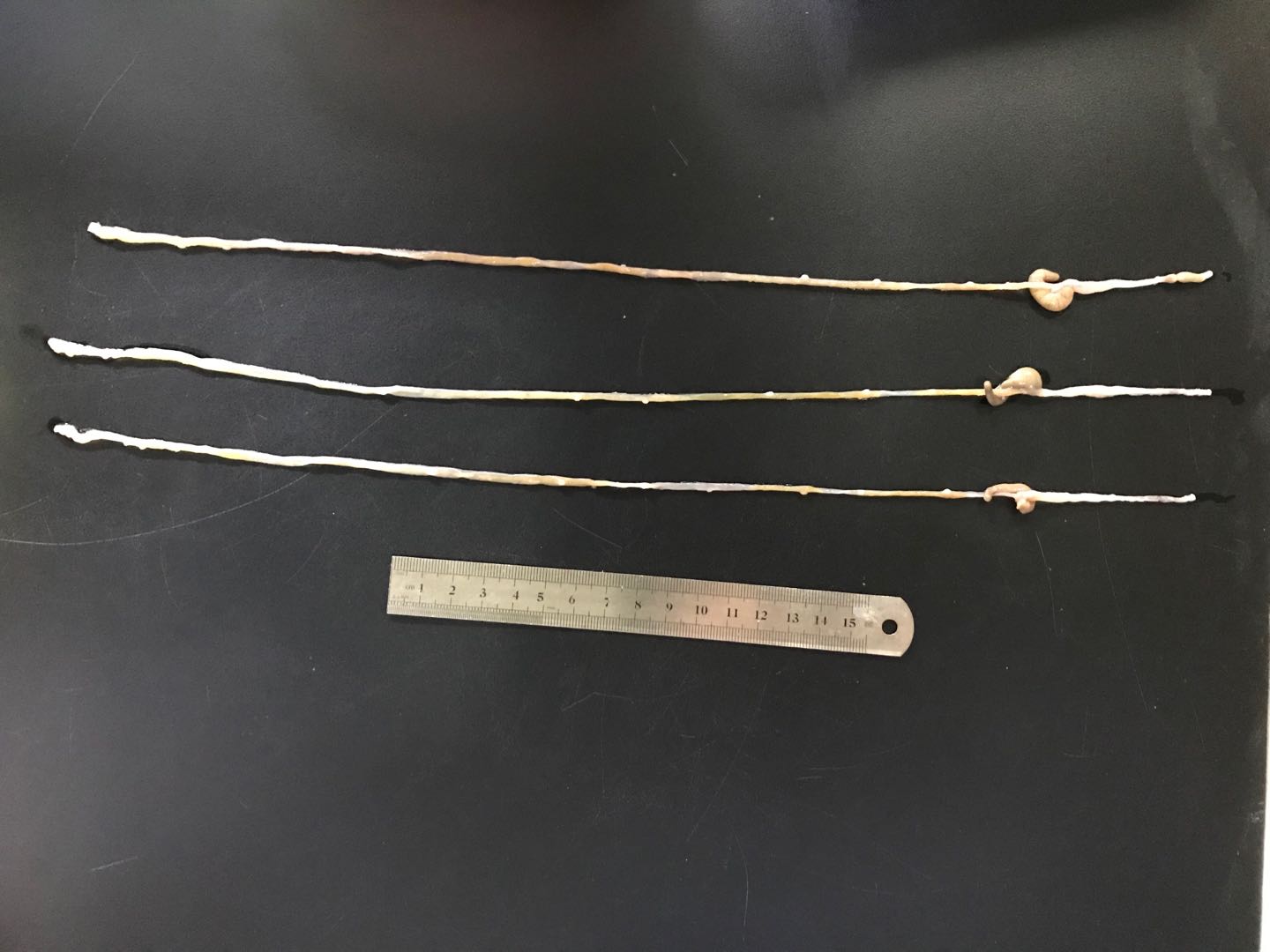

Supplement: Supplementary Figure 1 — (A) rtPCR verifying the expression of Hadh, Ifi204, Srpk1, Smad4 in the colon of DSS-induced colitis at day 0, 3, 5. (B) KEGG analysis the differential protein related pathway. (C) rtPCR show the expression of BMP2,4,7 in the colon of DSS-induced colitis at day 0, 3, 5. Data represent mean ± SD; one-way ANOVA and Bonferroni test for multiple testing. ∗∗p < 0.01, ∗∗∗p < 0.001. [file Data_Sheet_1.ZIP › fig1/5day.jpg]

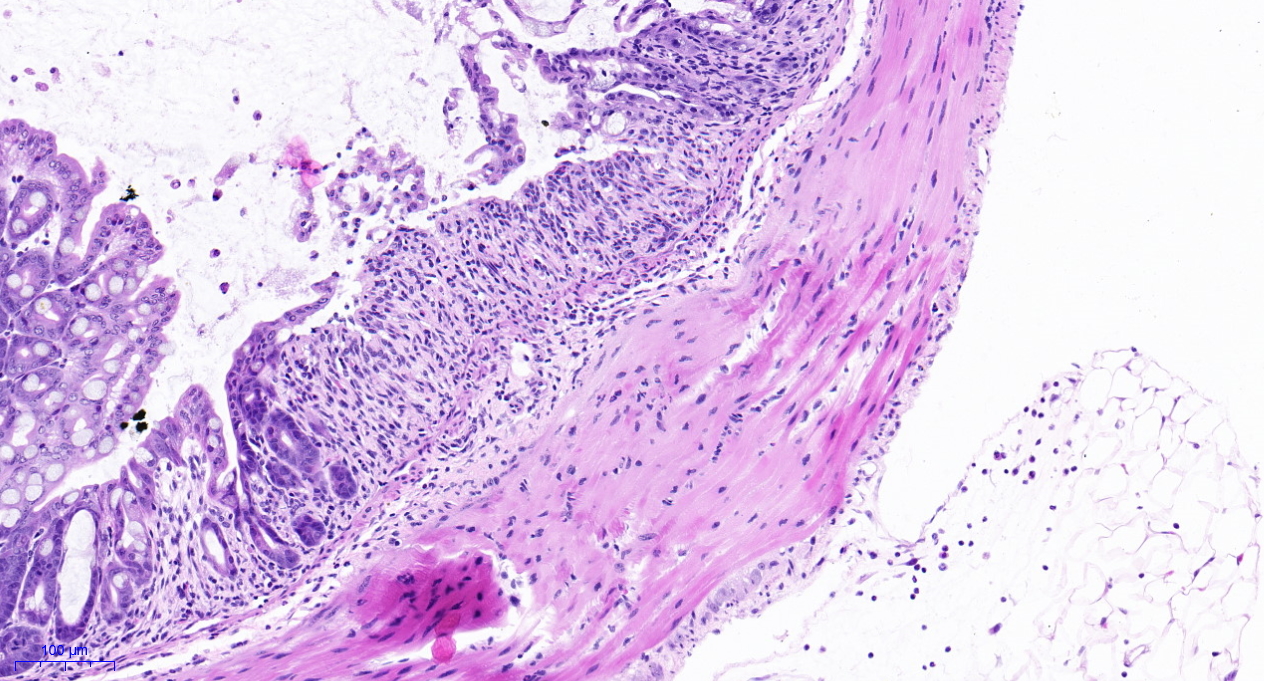

Supplement: Supplementary Figure 1 — (A) rtPCR verifying the expression of Hadh, Ifi204, Srpk1, Smad4 in the colon of DSS-induced colitis at day 0, 3, 5. (B) KEGG analysis the differential protein related pathway. (C) rtPCR show the expression of BMP2,4,7 in the colon of DSS-induced colitis at day 0, 3, 5. Data represent mean ± SD; one-way ANOVA and Bonferroni test for multiple testing. ∗∗p < 0.01, ∗∗∗p < 0.001. [file Data_Sheet_1.ZIP › fig1/5day_10.0x.jpg]

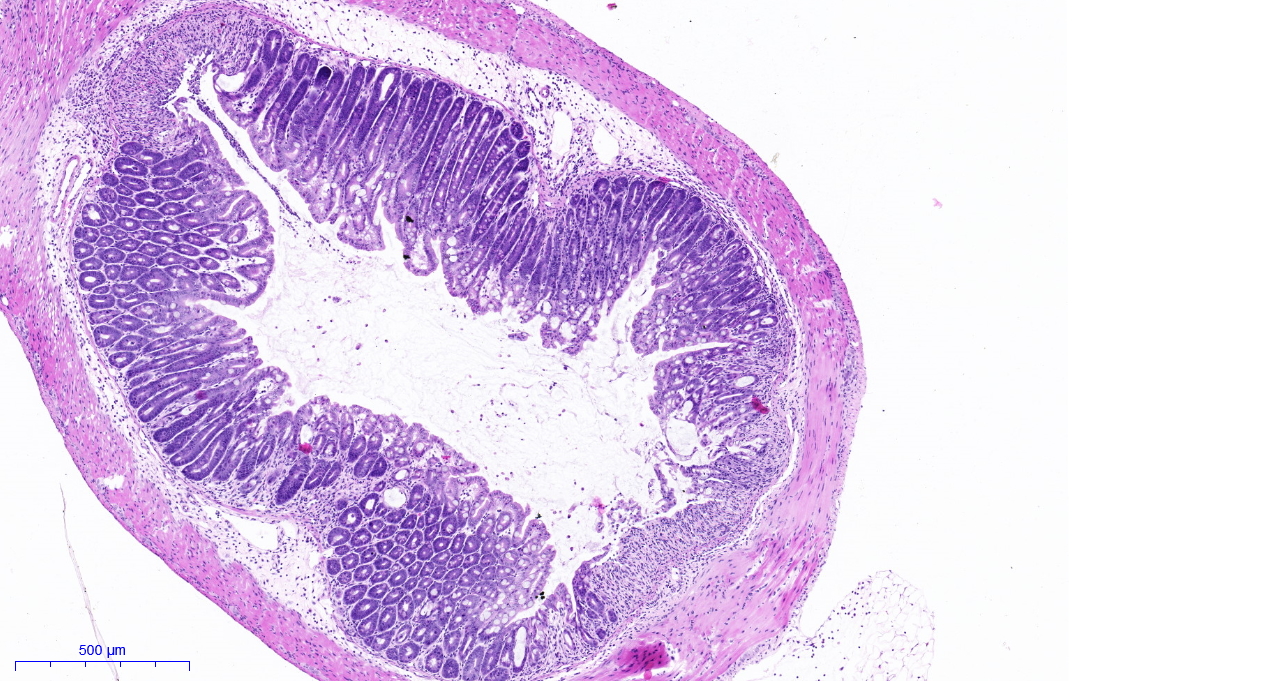

Supplement: Supplementary Figure 1 — (A) rtPCR verifying the expression of Hadh, Ifi204, Srpk1, Smad4 in the colon of DSS-induced colitis at day 0, 3, 5. (B) KEGG analysis the differential protein related pathway. (C) rtPCR show the expression of BMP2,4,7 in the colon of DSS-induced colitis at day 0, 3, 5. Data represent mean ± SD; one-way ANOVA and Bonferroni test for multiple testing. ∗∗p < 0.01, ∗∗∗p < 0.001. [file Data_Sheet_1.ZIP › fig1/5day_3.5x.jpg]

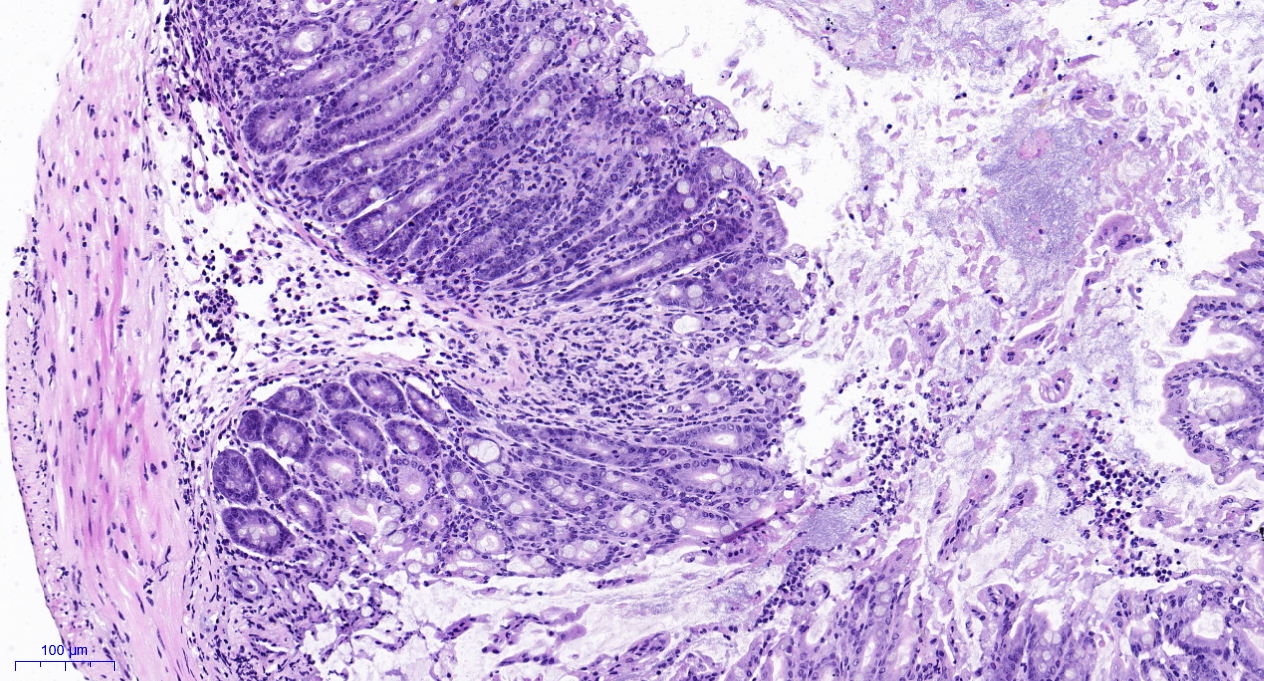

Supplement: Supplementary Figure 1 — (A) rtPCR verifying the expression of Hadh, Ifi204, Srpk1, Smad4 in the colon of DSS-induced colitis at day 0, 3, 5. (B) KEGG analysis the differential protein related pathway. (C) rtPCR show the expression of BMP2,4,7 in the colon of DSS-induced colitis at day 0, 3, 5. Data represent mean ± SD; one-way ANOVA and Bonferroni test for multiple testing. ∗∗p < 0.01, ∗∗∗p < 0.001. [file Data_Sheet_1.ZIP › fig1/7day-1_10.0x.jpg]

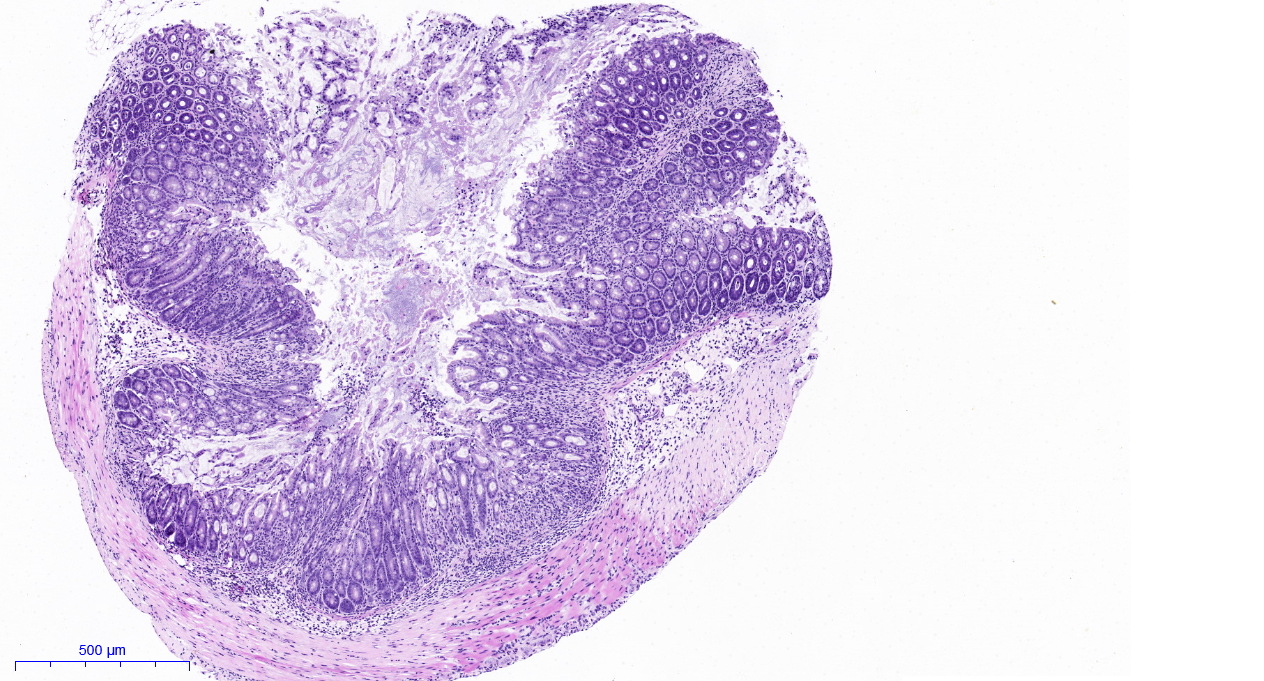

Supplement: Supplementary Figure 1 — (A) rtPCR verifying the expression of Hadh, Ifi204, Srpk1, Smad4 in the colon of DSS-induced colitis at day 0, 3, 5. (B) KEGG analysis the differential protein related pathway. (C) rtPCR show the expression of BMP2,4,7 in the colon of DSS-induced colitis at day 0, 3, 5. Data represent mean ± SD; one-way ANOVA and Bonferroni test for multiple testing. ∗∗p < 0.01, ∗∗∗p < 0.001. [file Data_Sheet_1.ZIP › fig1/7day-1_3.5x.jpg]

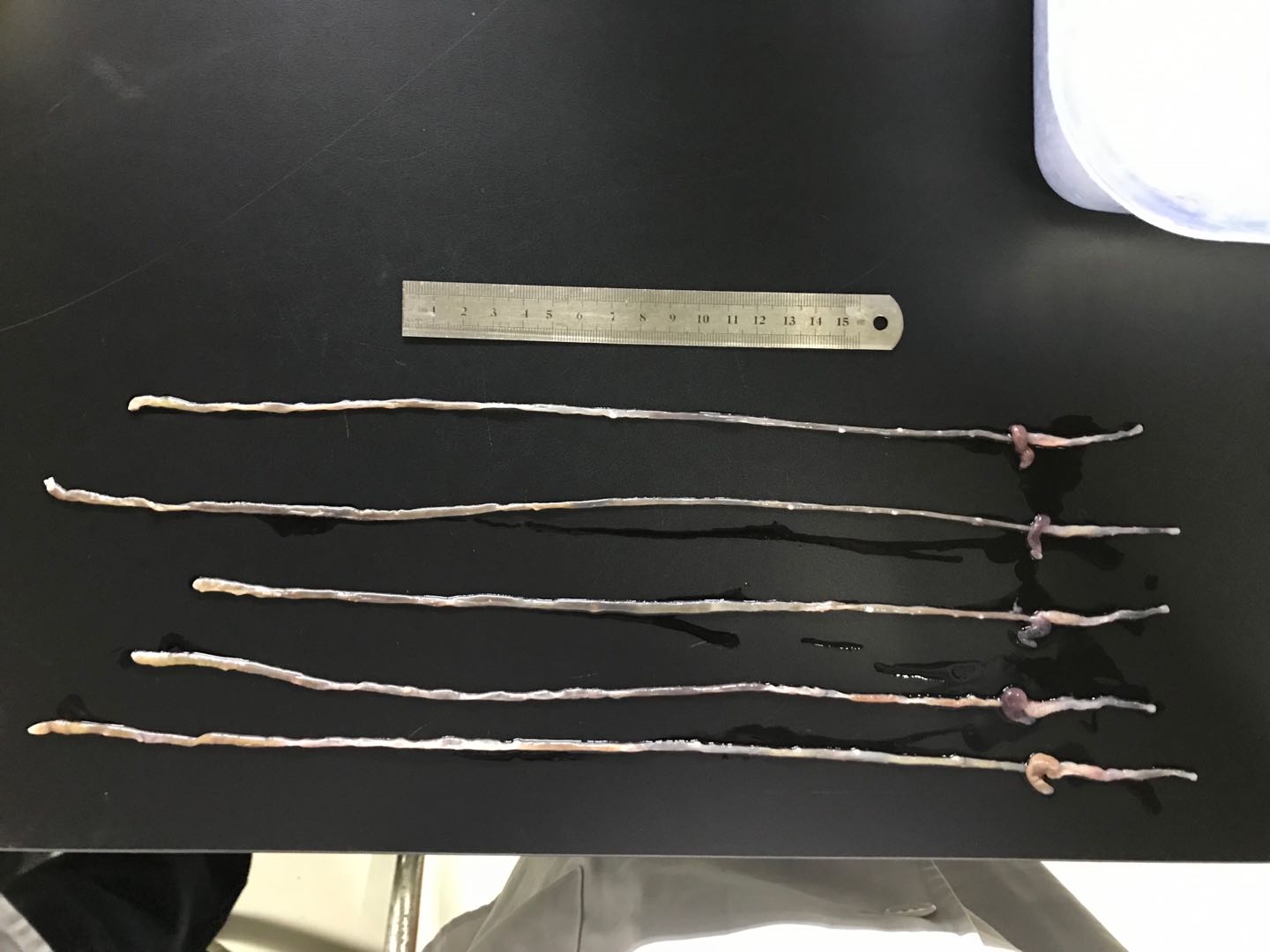

Supplement: Supplementary Figure 1 — (A) rtPCR verifying the expression of Hadh, Ifi204, Srpk1, Smad4 in the colon of DSS-induced colitis at day 0, 3, 5. (B) KEGG analysis the differential protein related pathway. (C) rtPCR show the expression of BMP2,4,7 in the colon of DSS-induced colitis at day 0, 3, 5. Data represent mean ± SD; one-way ANOVA and Bonferroni test for multiple testing. ∗∗p < 0.01, ∗∗∗p < 0.001. [file Data_Sheet_1.ZIP › fig1/7day.jpg]

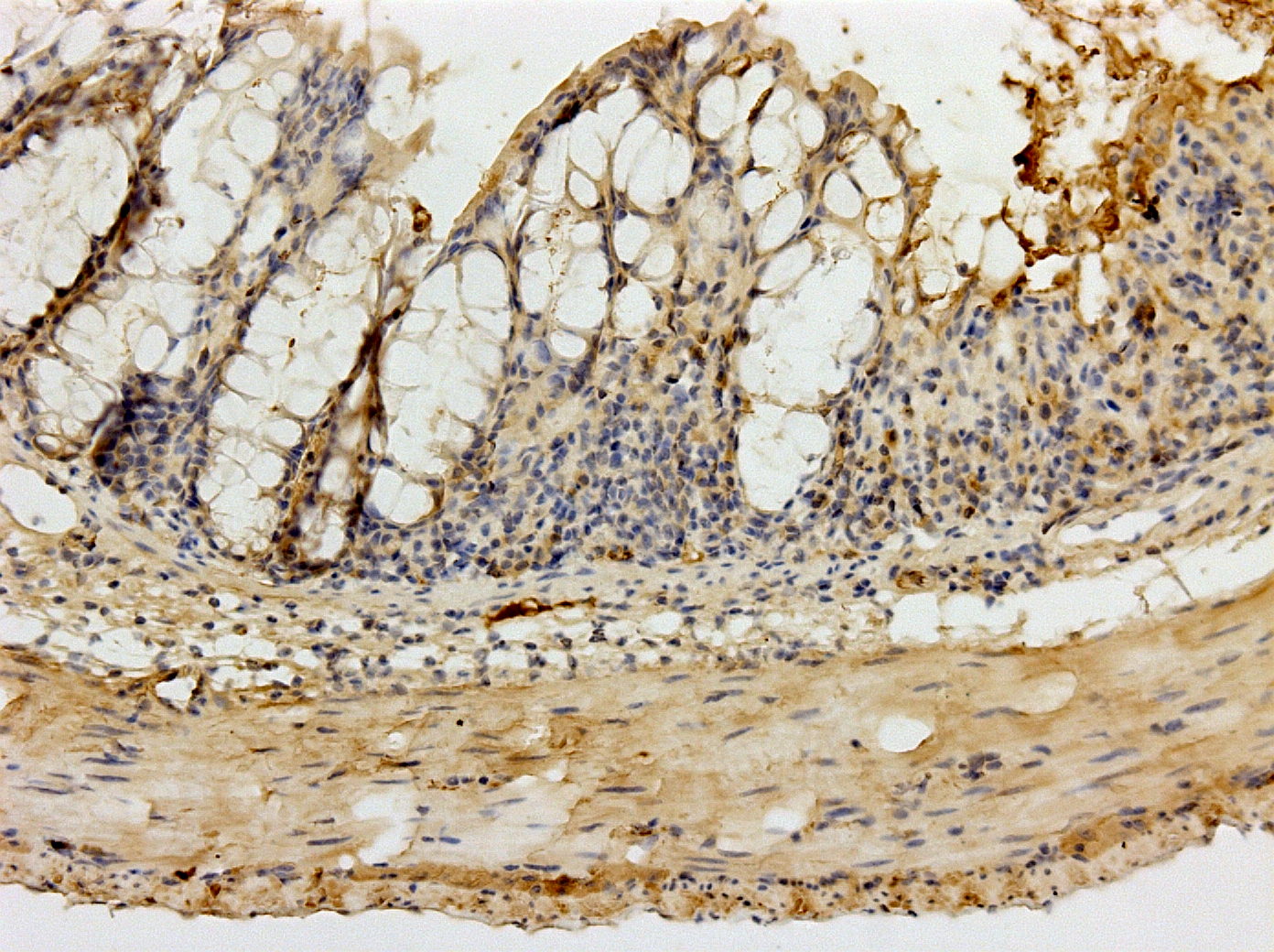

Supplement: Supplementary file 2 [file Data_Sheet_2.zip › fig3/3 days.tif]

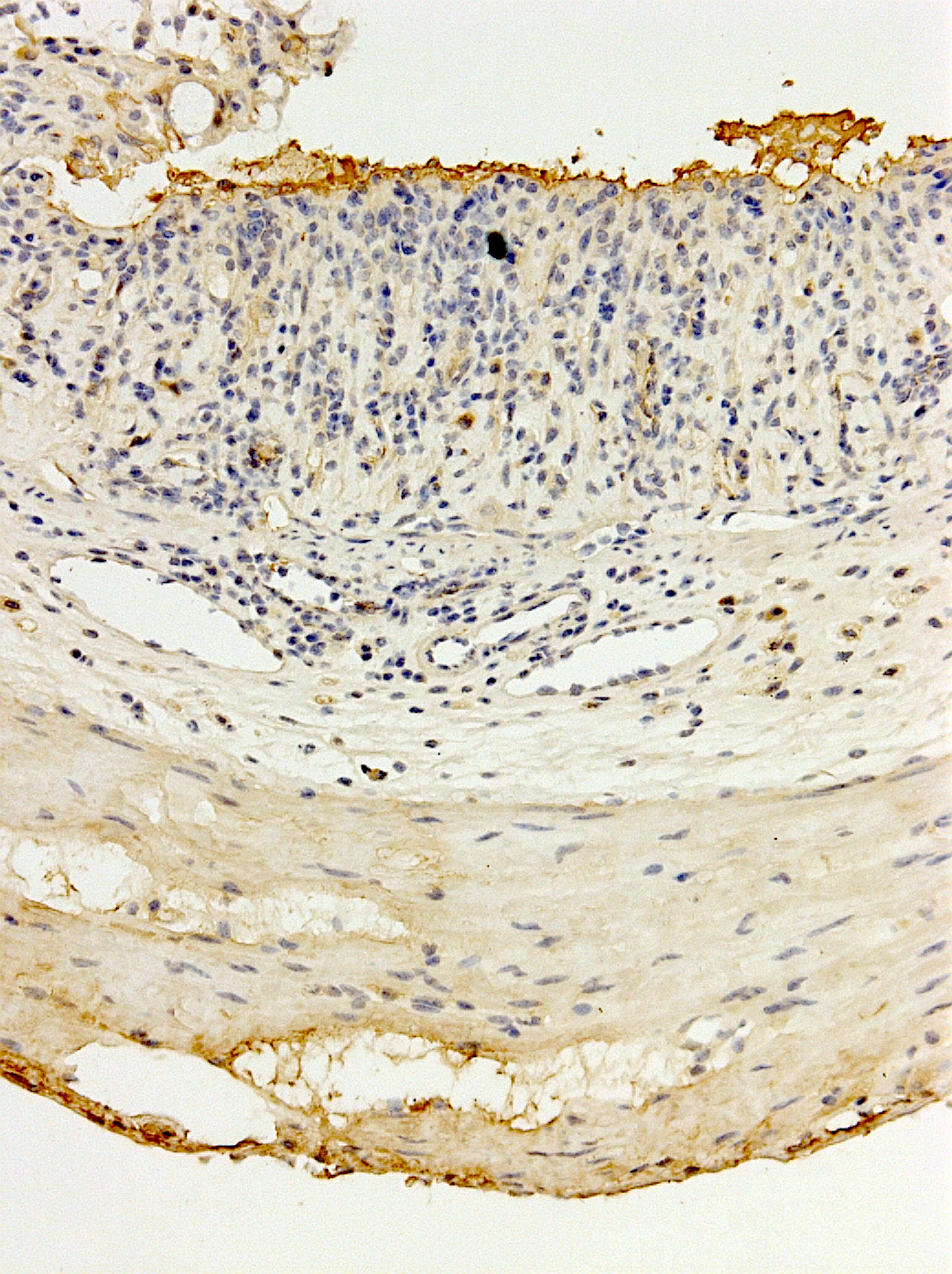

Supplement: Supplementary file 2 [file Data_Sheet_2.zip › fig3/7 days.tif]

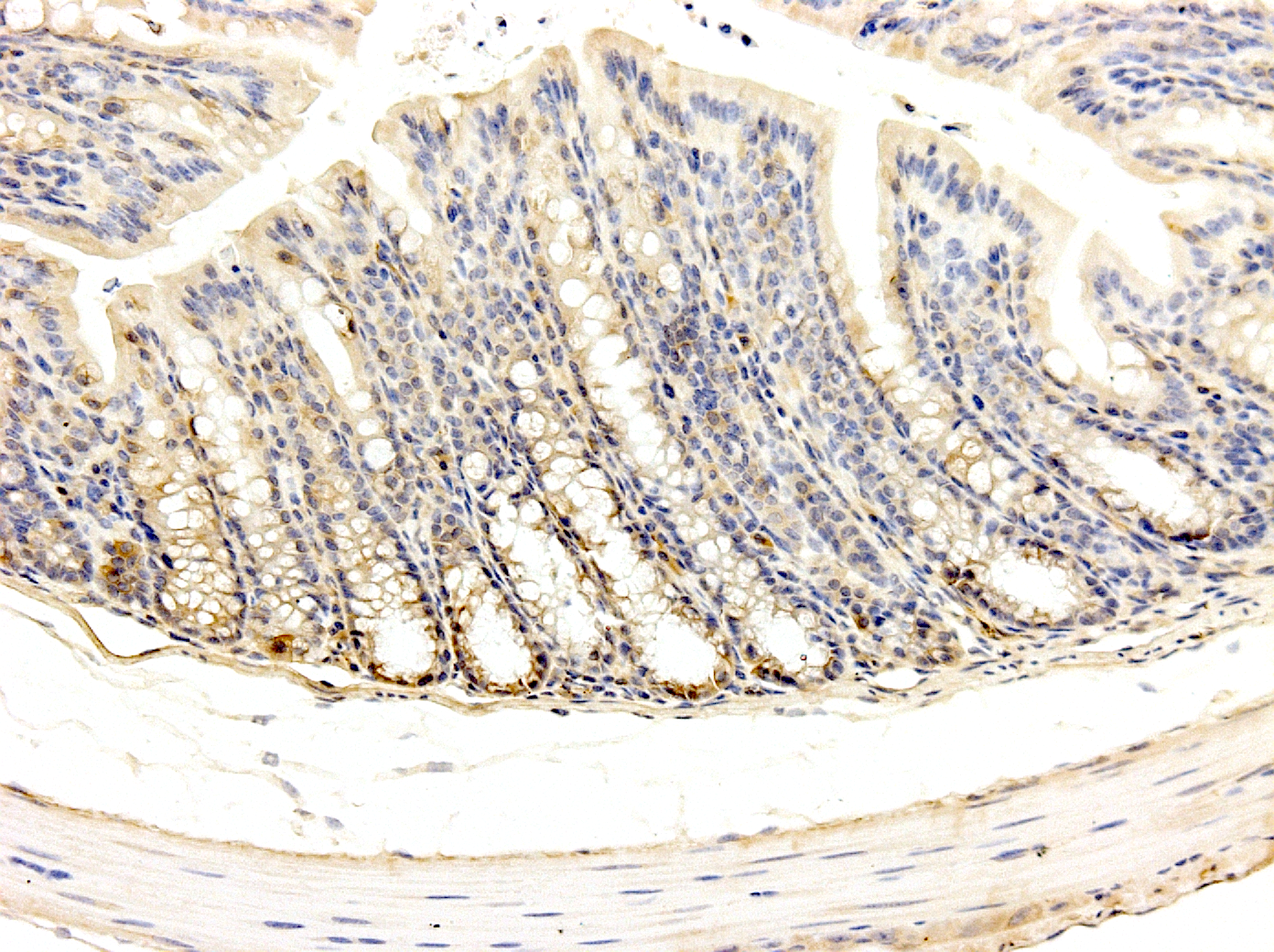

Supplement: Supplementary file 2 [file Data_Sheet_2.zip › fig3/mormal1-1.tif]

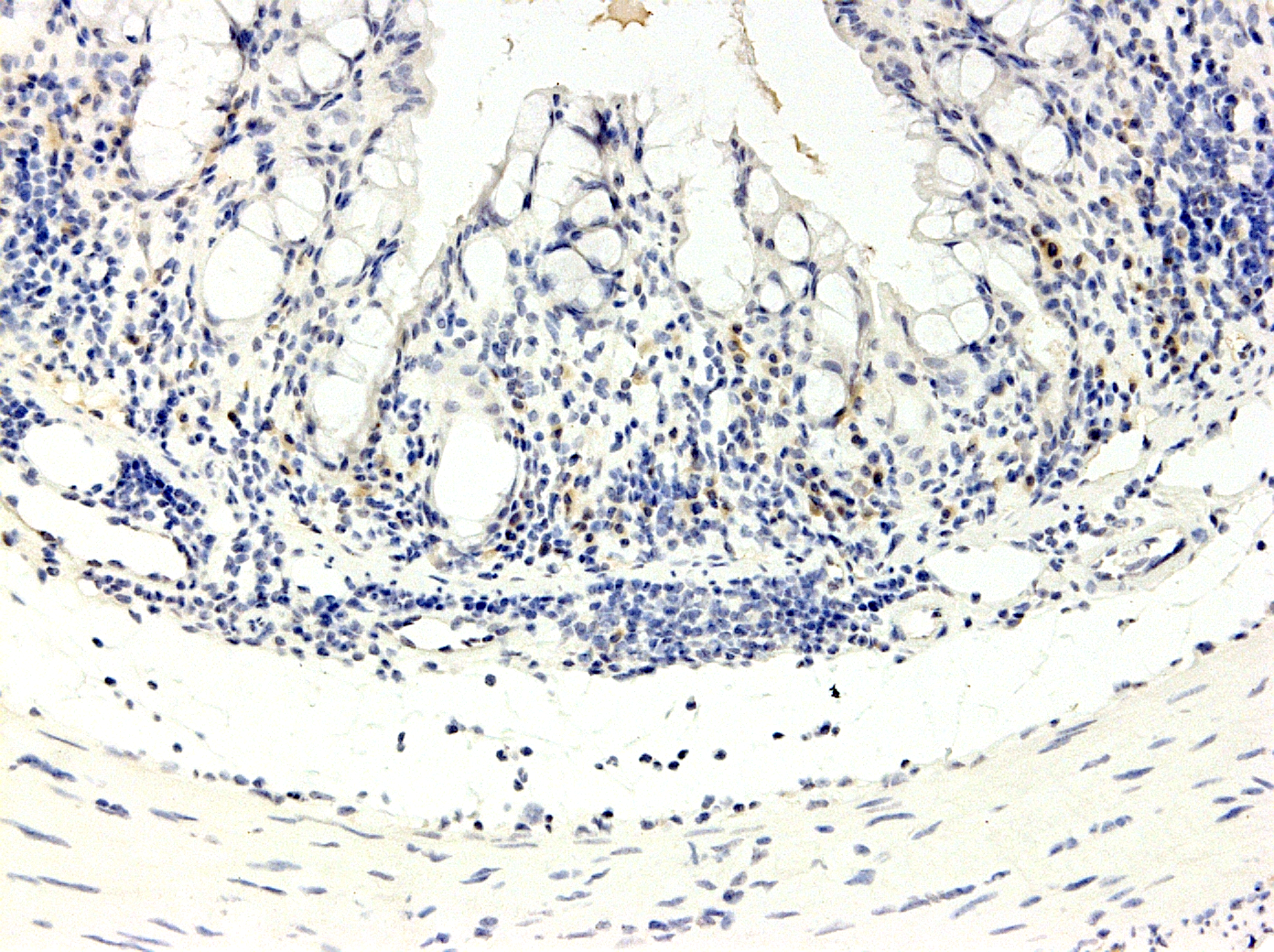

Supplement: Supplementary file 2 [file Data_Sheet_2.zip › fig3/smad4-1.tif]

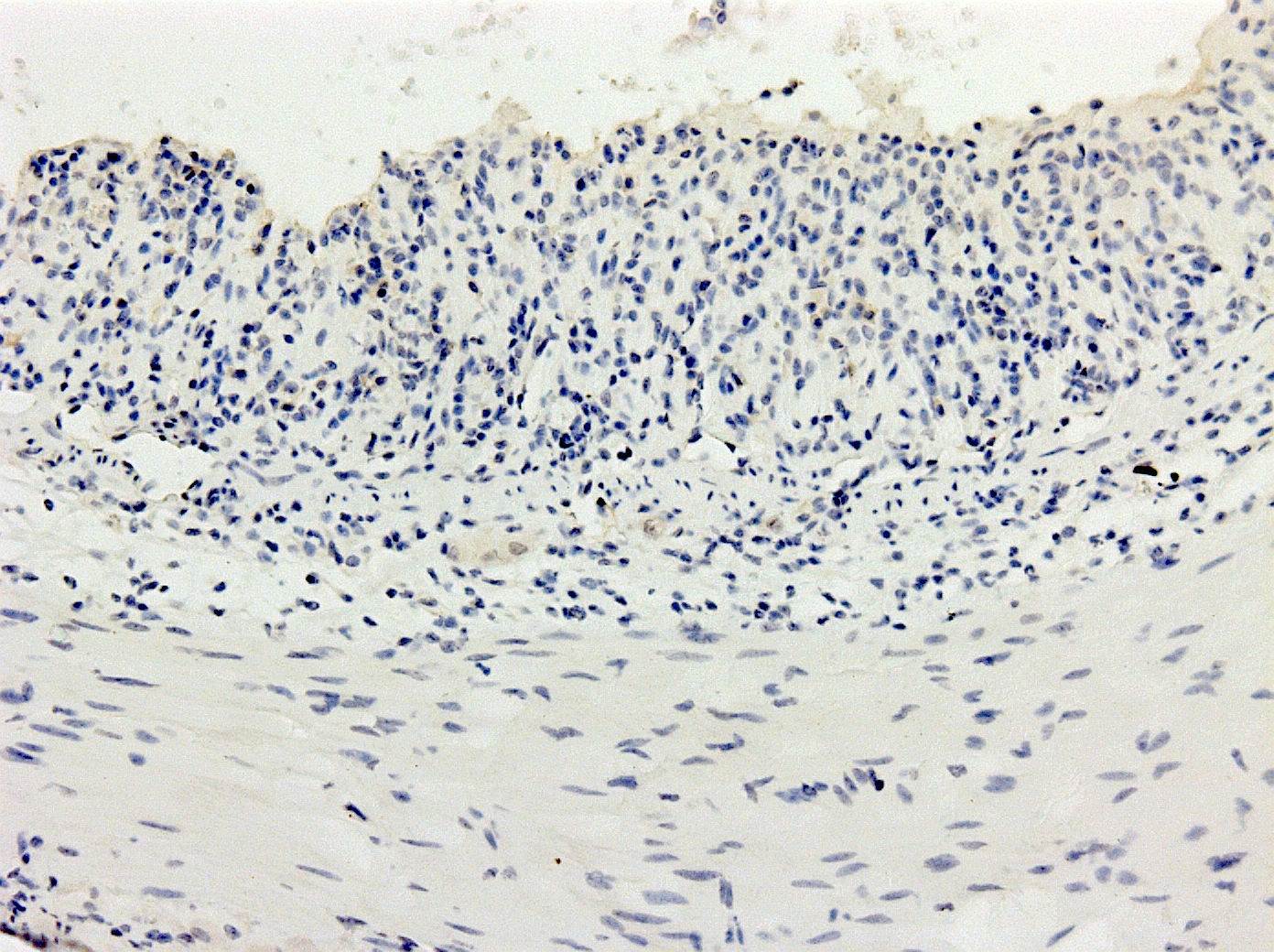

Supplement: Supplementary file 2 [file Data_Sheet_2.zip › fig3/smad4-2.tif]

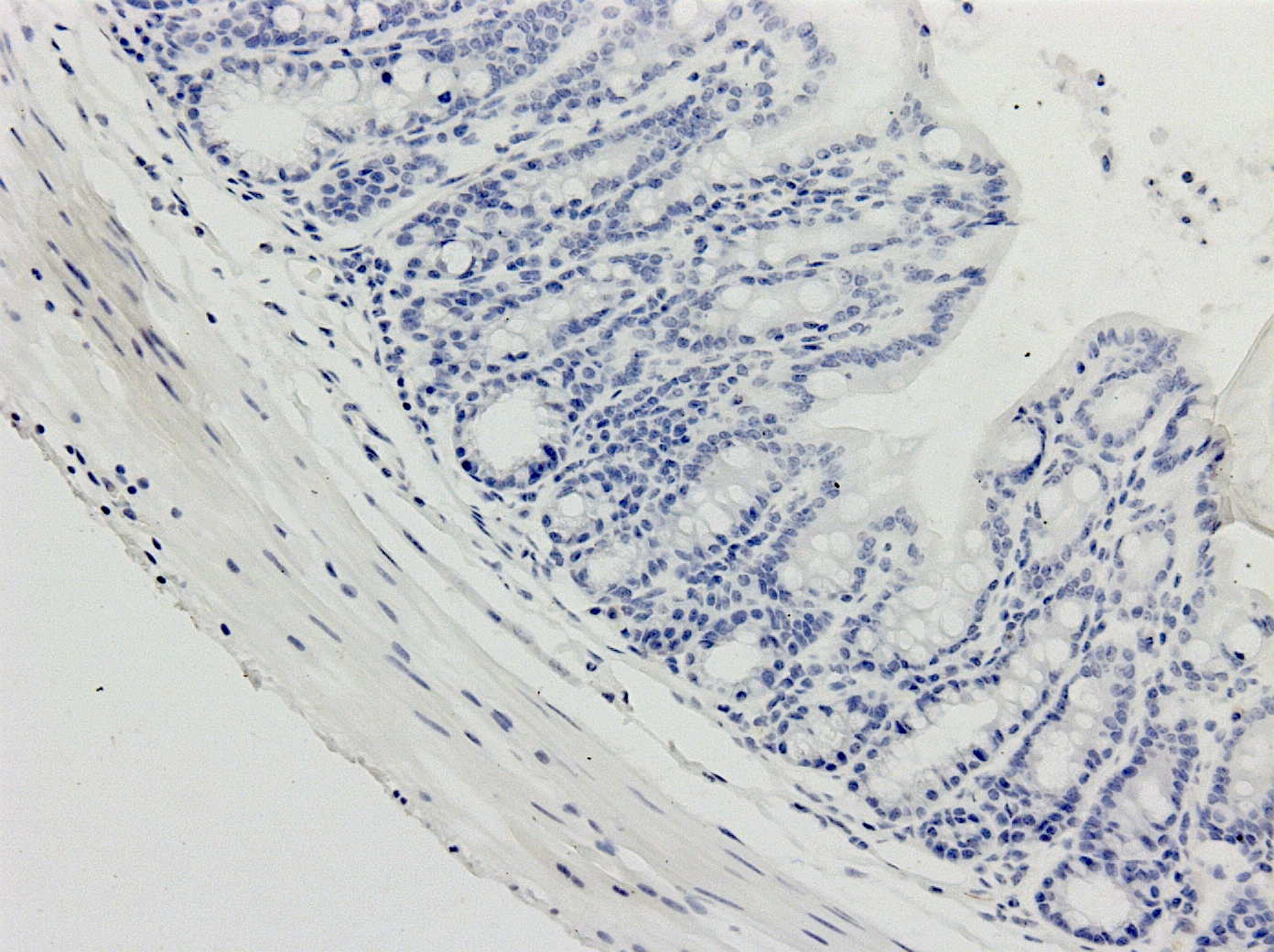

Supplement: Supplementary file 2 [file Data_Sheet_2.zip › fig3/smad4.tif]

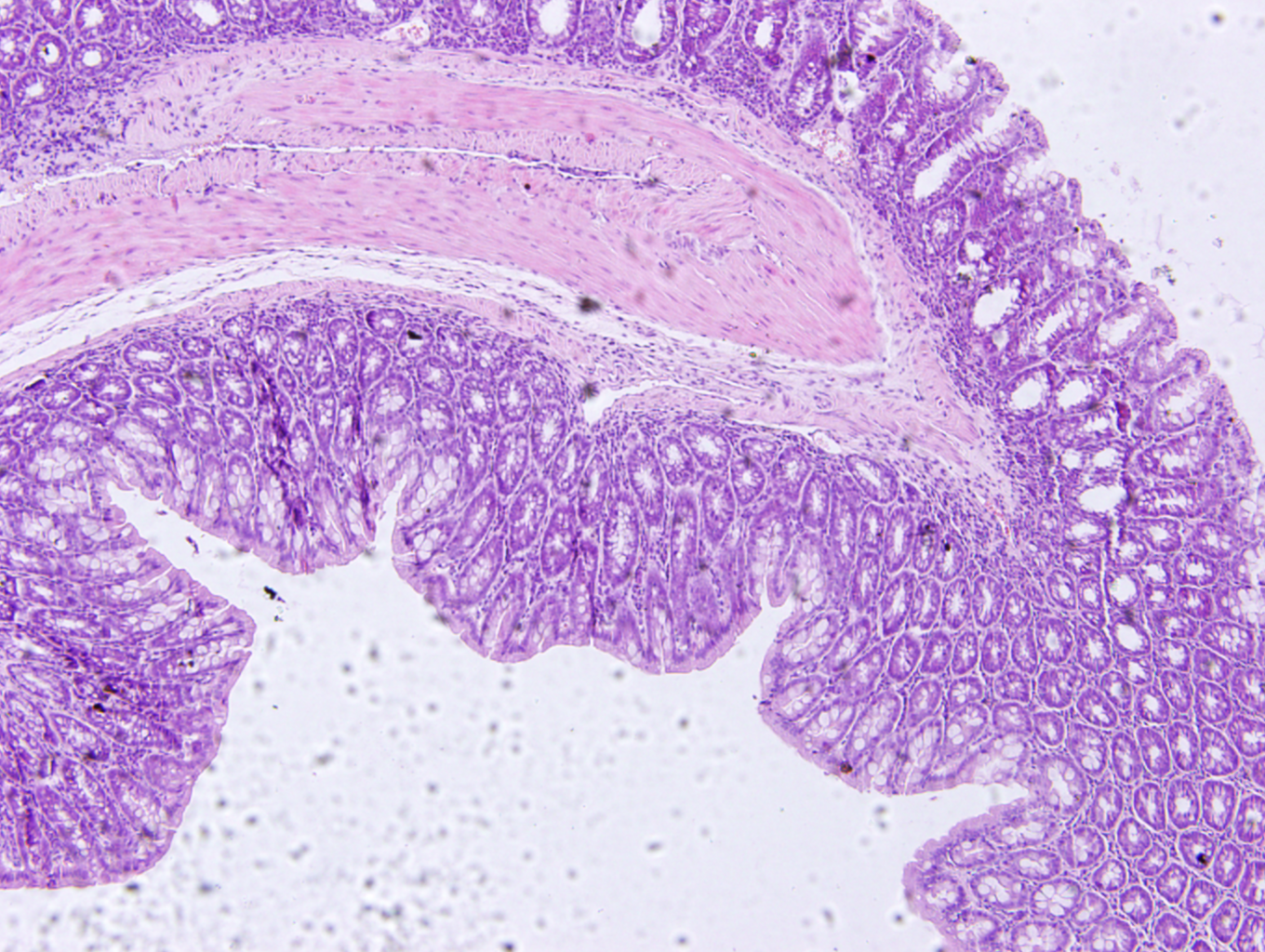

Supplement: Supplementary file 3 [file Data_Sheet_3.ZIP › fig 6 raw data-2/ko Image_1569.tif]

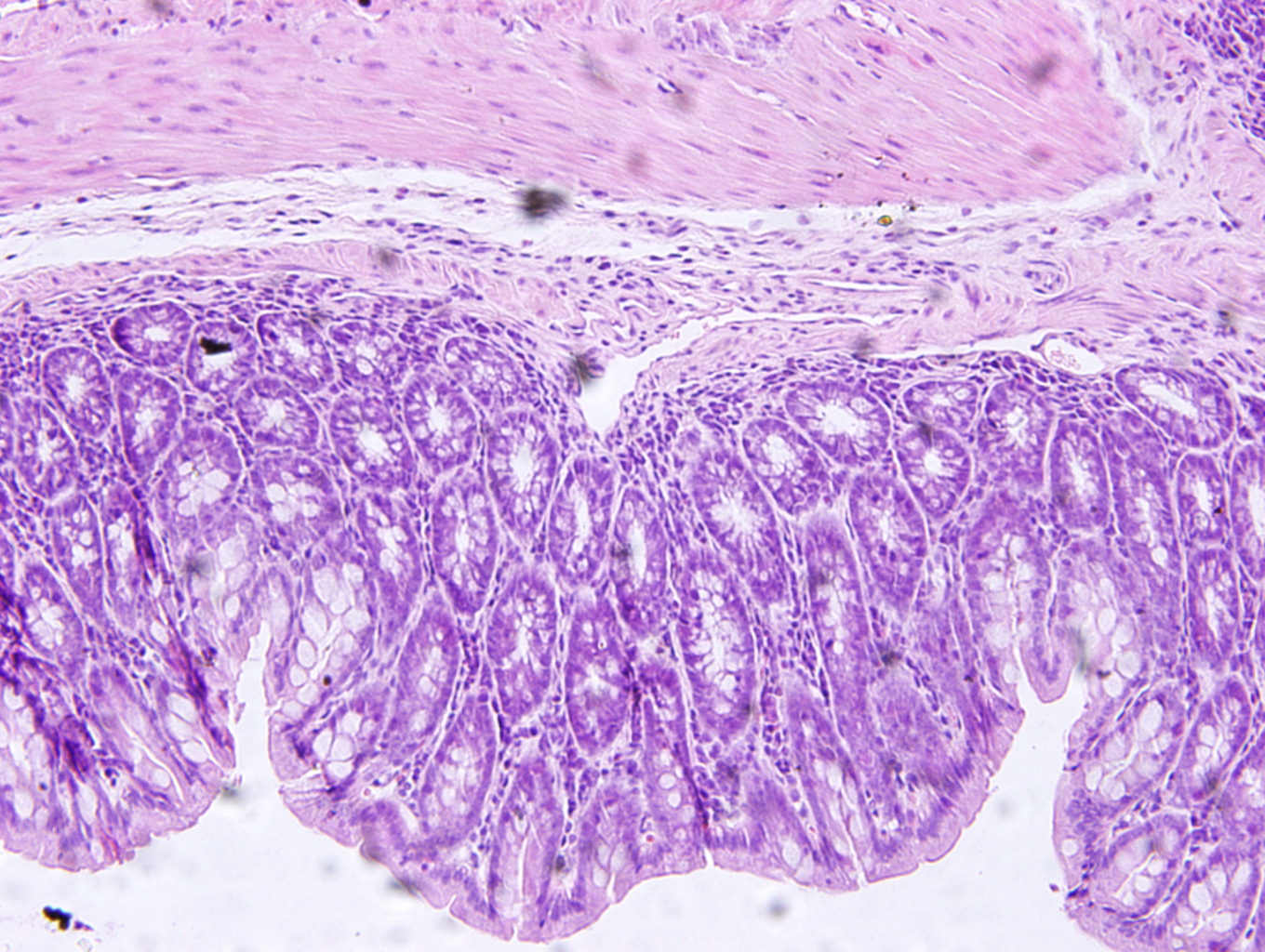

Supplement: Supplementary file 3 [file Data_Sheet_3.ZIP › fig 6 raw data-2/ko Image_1570.tif]

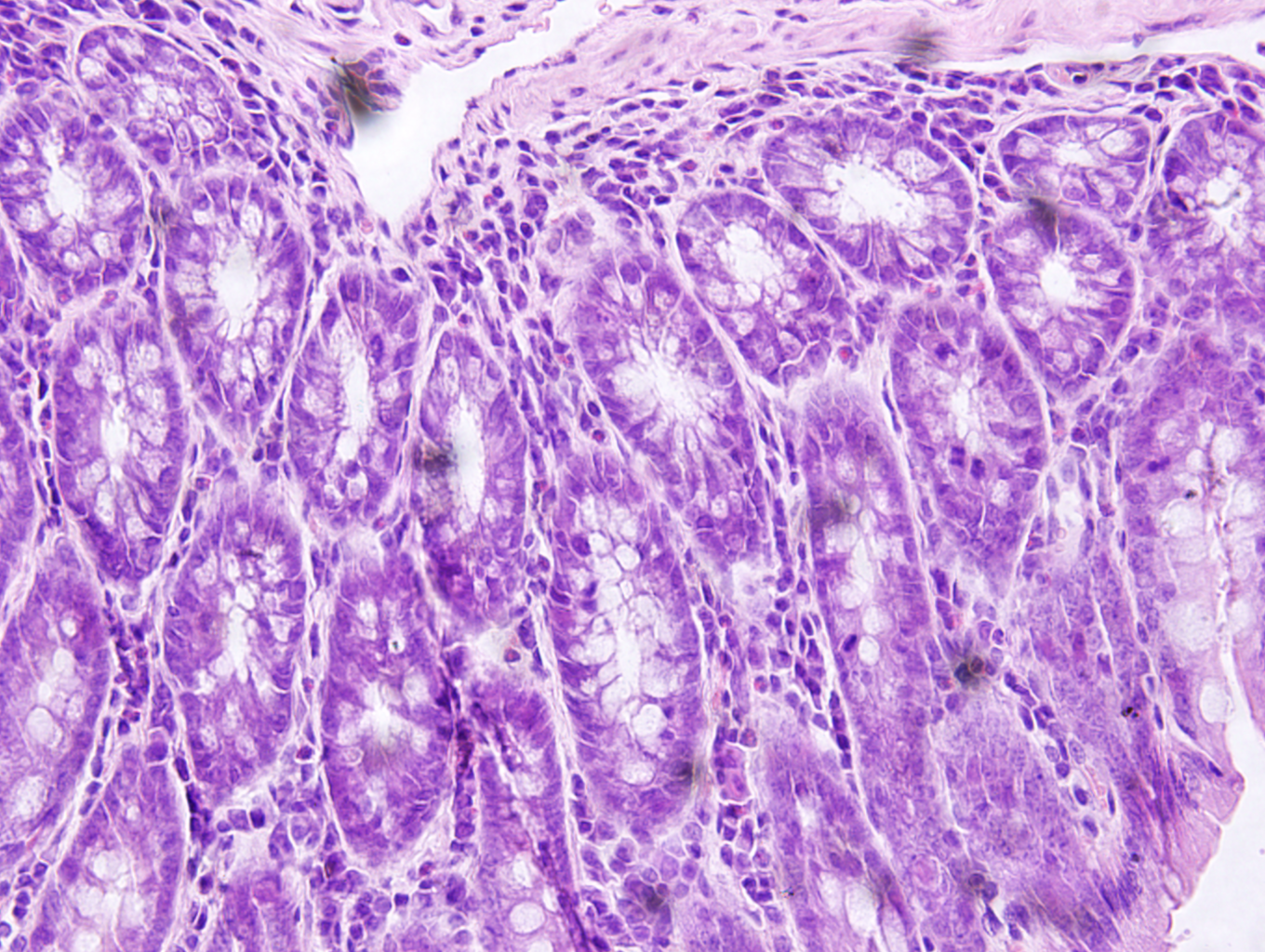

Supplement: Supplementary file 3 [file Data_Sheet_3.ZIP › fig 6 raw data-2/ko Image_1571.tif]

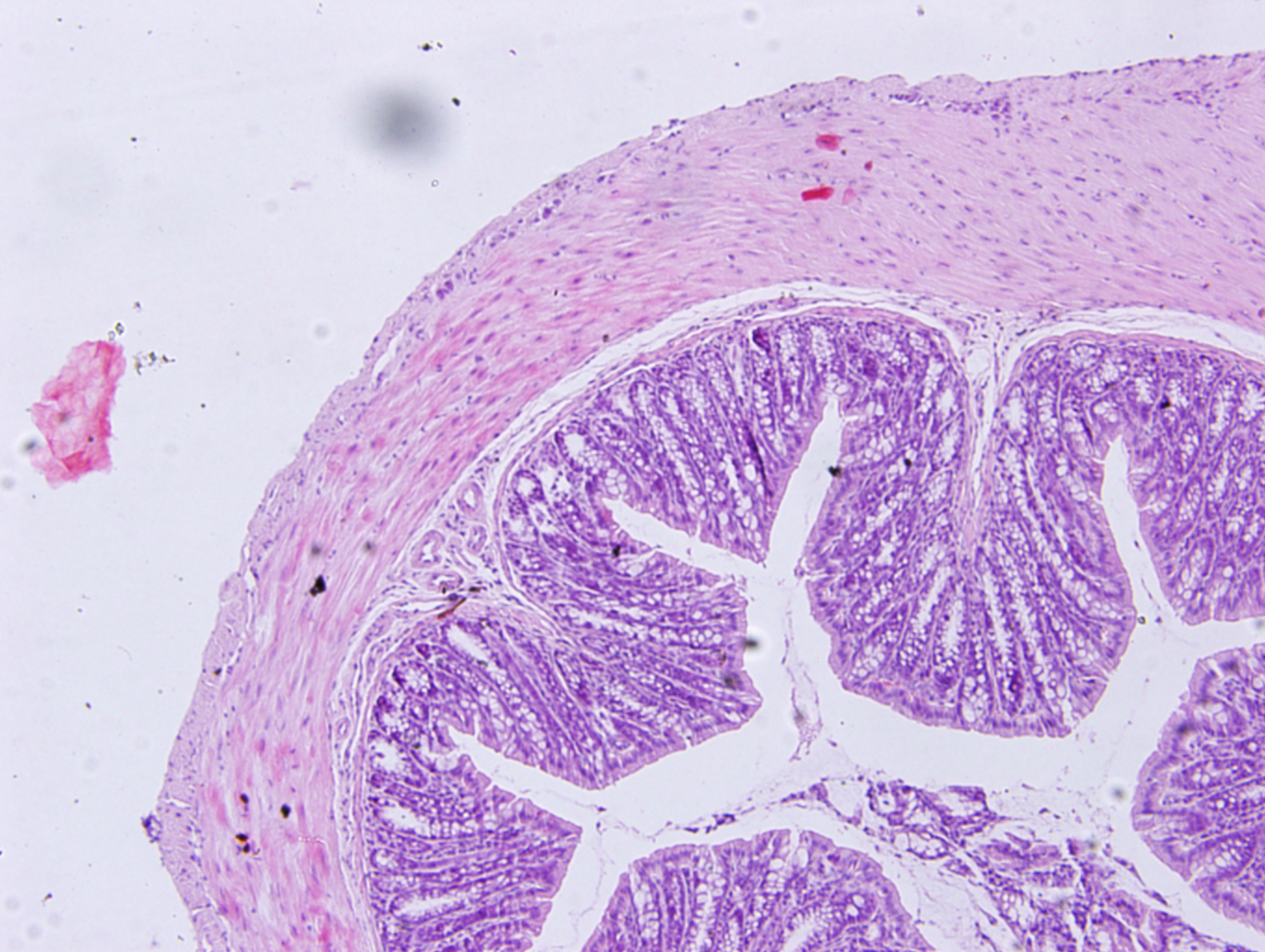

Supplement: Supplementary file 3 [file Data_Sheet_3.ZIP › fig 6 raw data-2/wt Image_1566.tif]

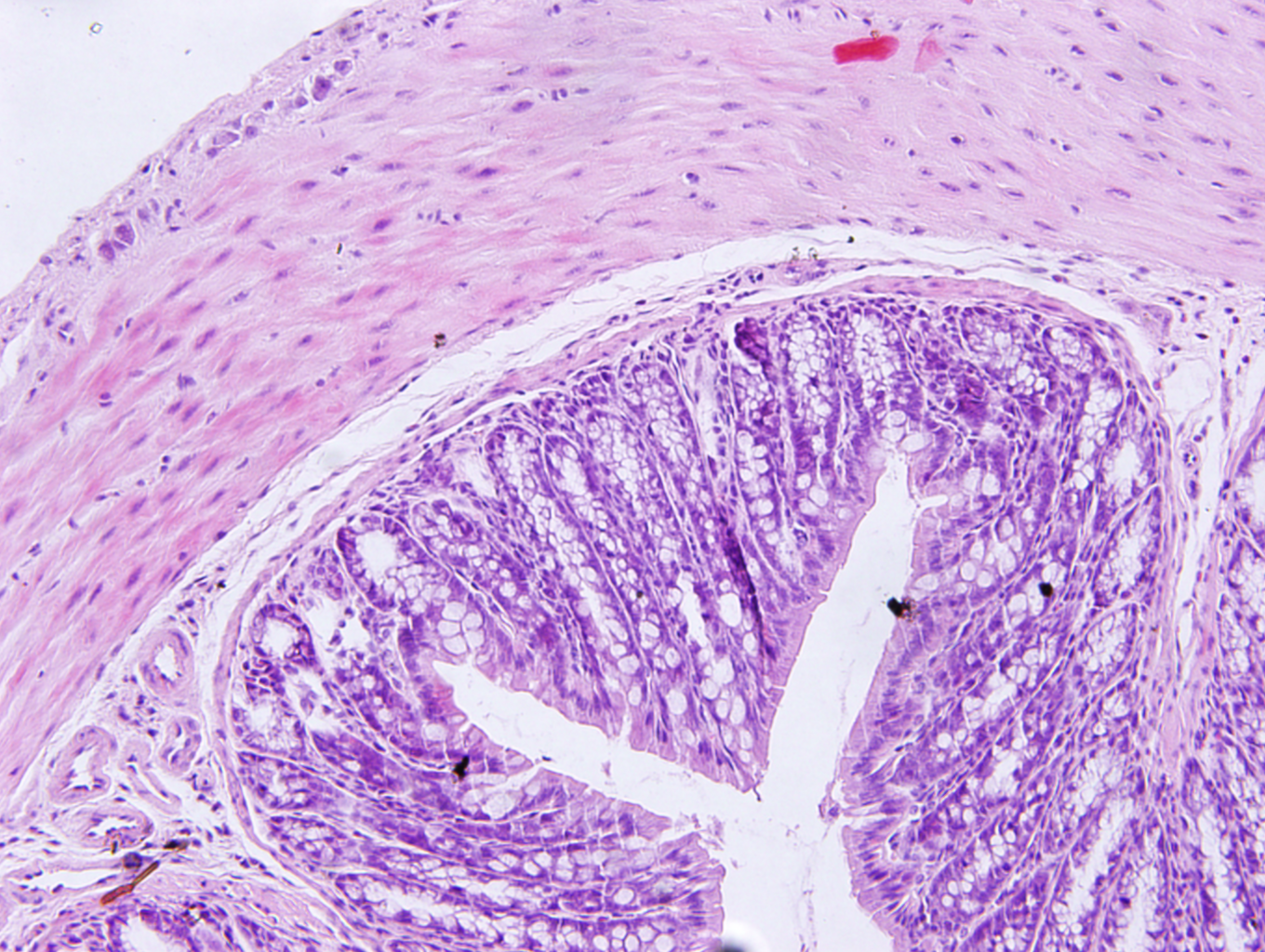

Supplement: Supplementary file 3 [file Data_Sheet_3.ZIP › fig 6 raw data-2/wt Image_1567.tif]

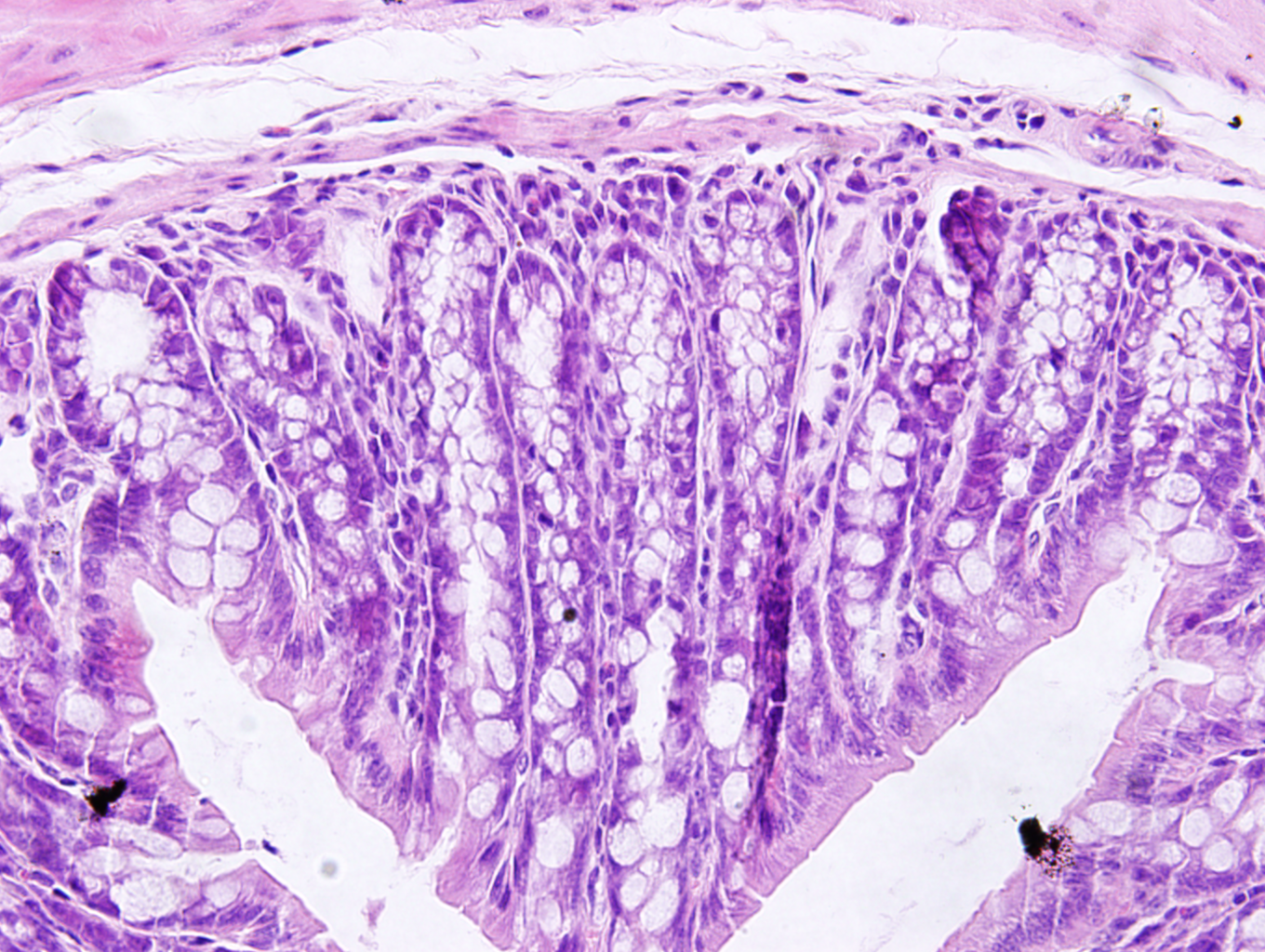

Supplement: Supplementary file 3 [file Data_Sheet_3.ZIP › fig 6 raw data-2/wt Image_1568.tif]

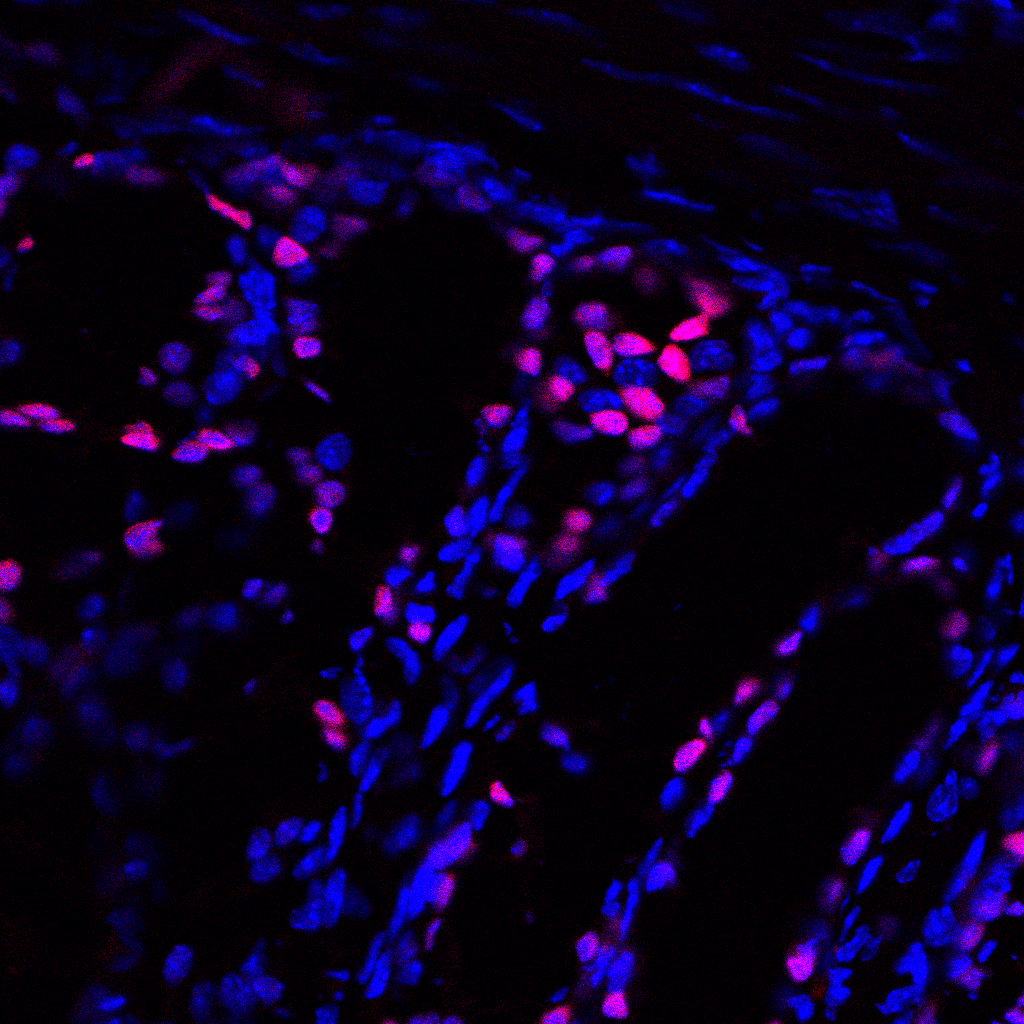

Supplement: Supplementary Figure 2 — (A) Masson’s trichrome staining showed the collagen deposition in each group, no significant difference between BMP4 recombinant protein and BMP4 antibody treatment. (B) Masson’s trichrome staining showed no significant difference of collagen deposition between Id3–/– aged mice and wild type aged mice. Scale bar = 50 μm. [file Data_Sheet_4.ZIP › fig2/0 ki67-1.tif]

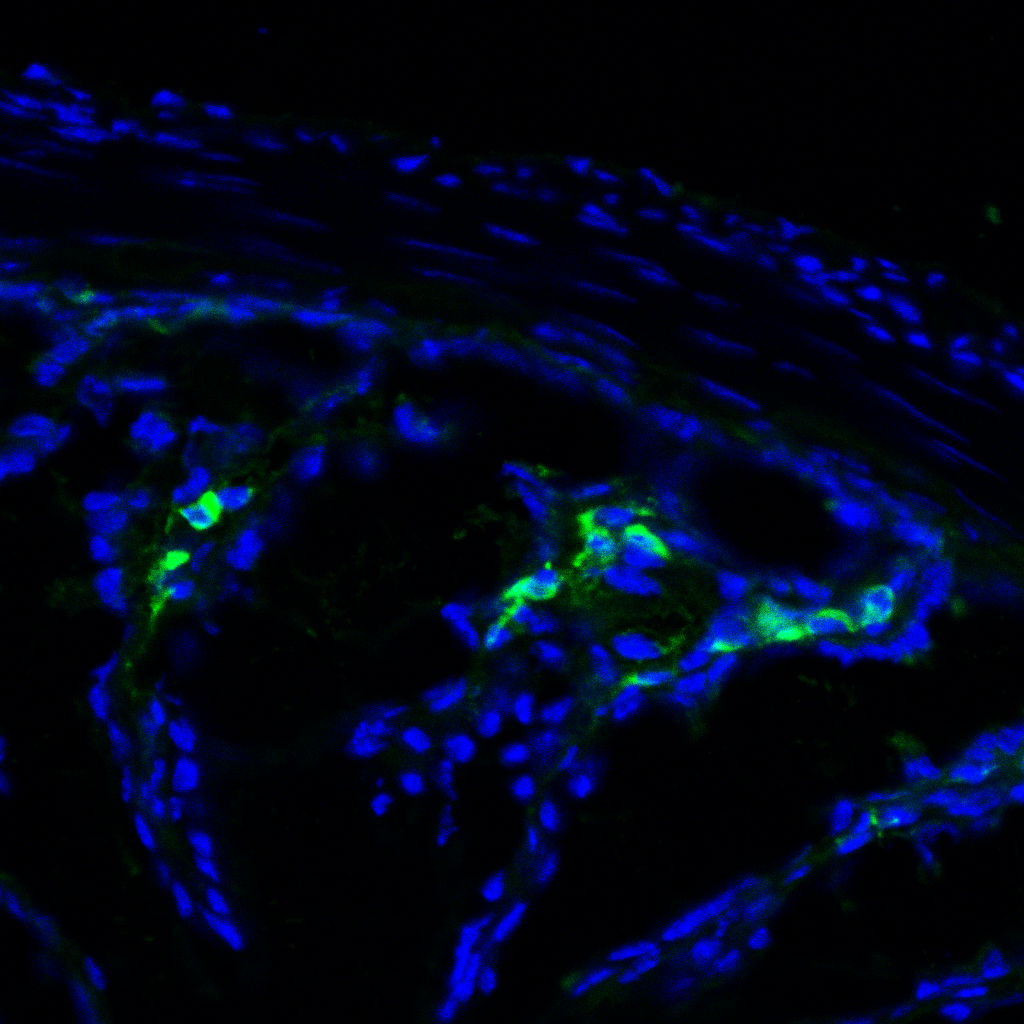

Supplement: Supplementary Figure 2 — (A) Masson’s trichrome staining showed the collagen deposition in each group, no significant difference between BMP4 recombinant protein and BMP4 antibody treatment. (B) Masson’s trichrome staining showed no significant difference of collagen deposition between Id3–/– aged mice and wild type aged mice. Scale bar = 50 μm. [file Data_Sheet_4.ZIP › fig2/0day lgr5.tif]

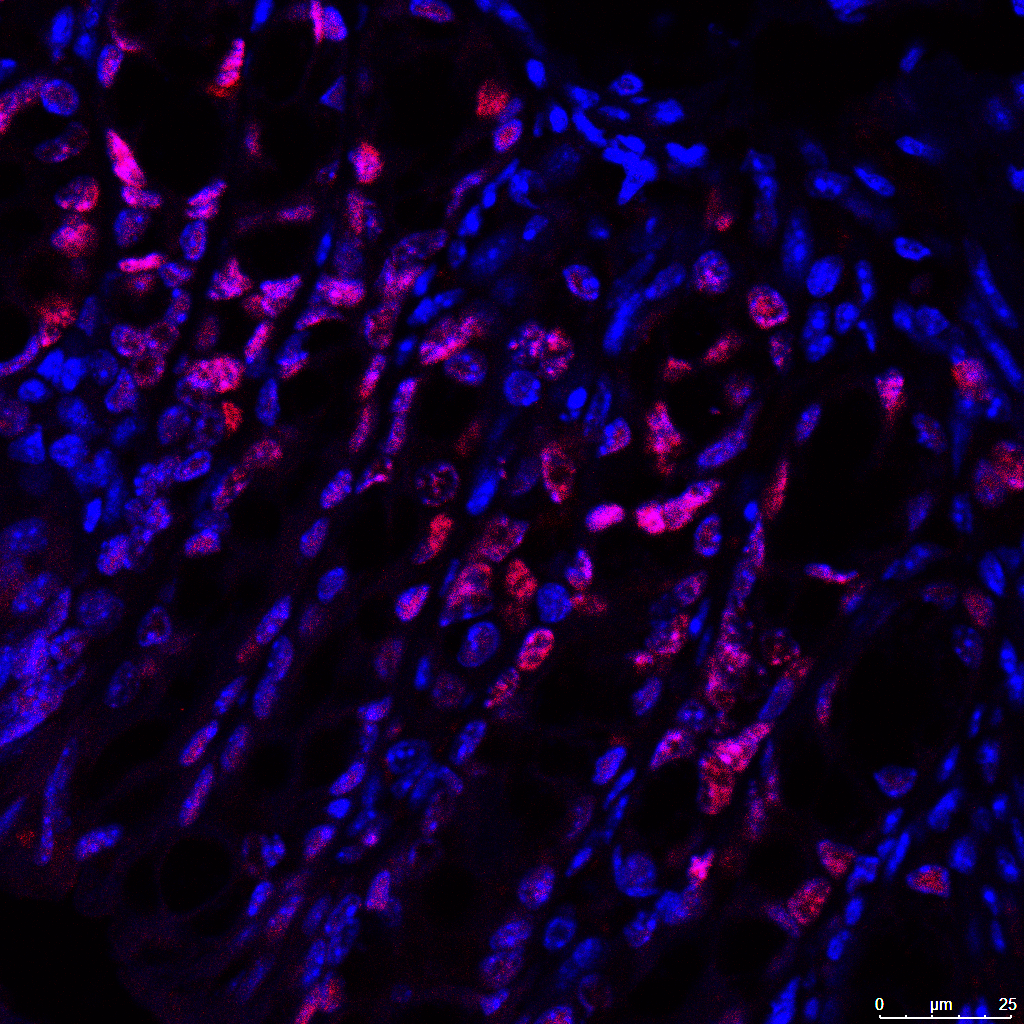

Supplement: Supplementary Figure 2 — (A) Masson’s trichrome staining showed the collagen deposition in each group, no significant difference between BMP4 recombinant protein and BMP4 antibody treatment. (B) Masson’s trichrome staining showed no significant difference of collagen deposition between Id3–/– aged mice and wild type aged mice. Scale bar = 50 μm. [file Data_Sheet_4.ZIP › fig2/3 day.tif]

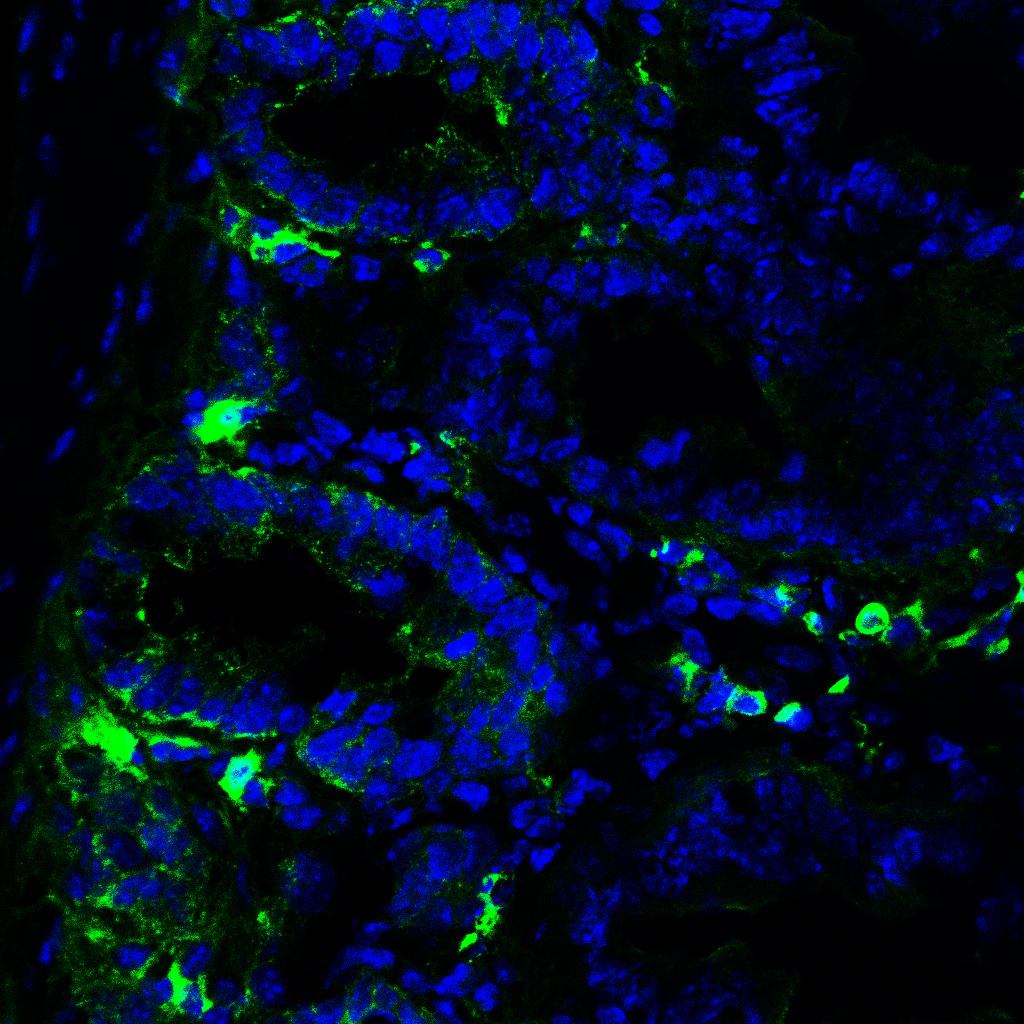

Supplement: Supplementary Figure 2 — (A) Masson’s trichrome staining showed the collagen deposition in each group, no significant difference between BMP4 recombinant protein and BMP4 antibody treatment. (B) Masson’s trichrome staining showed no significant difference of collagen deposition between Id3–/– aged mice and wild type aged mice. Scale bar = 50 μm. [file Data_Sheet_4.ZIP › fig2/3day lgr5.tif]

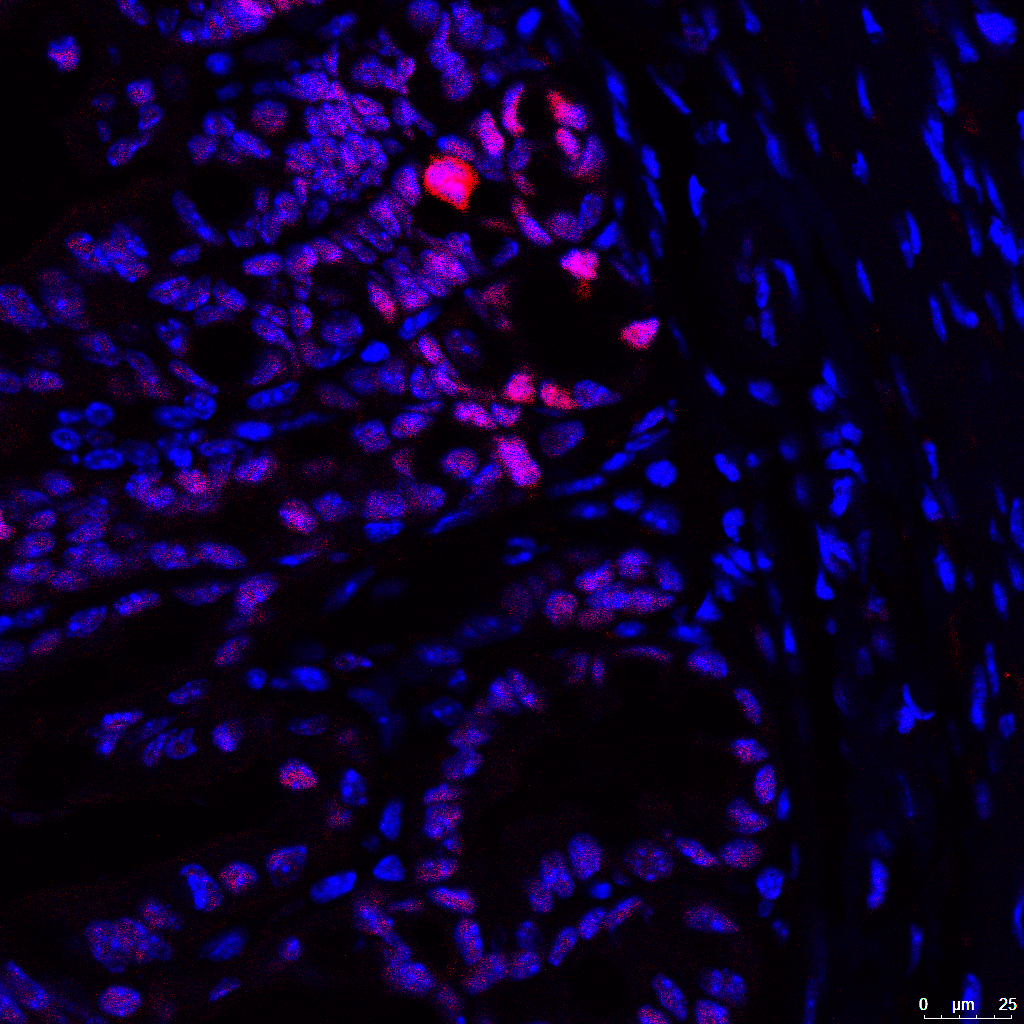

Supplement: Supplementary Figure 2 — (A) Masson’s trichrome staining showed the collagen deposition in each group, no significant difference between BMP4 recombinant protein and BMP4 antibody treatment. (B) Masson’s trichrome staining showed no significant difference of collagen deposition between Id3–/– aged mice and wild type aged mice. Scale bar = 50 μm. [file Data_Sheet_4.ZIP › fig2/5_day.tif]

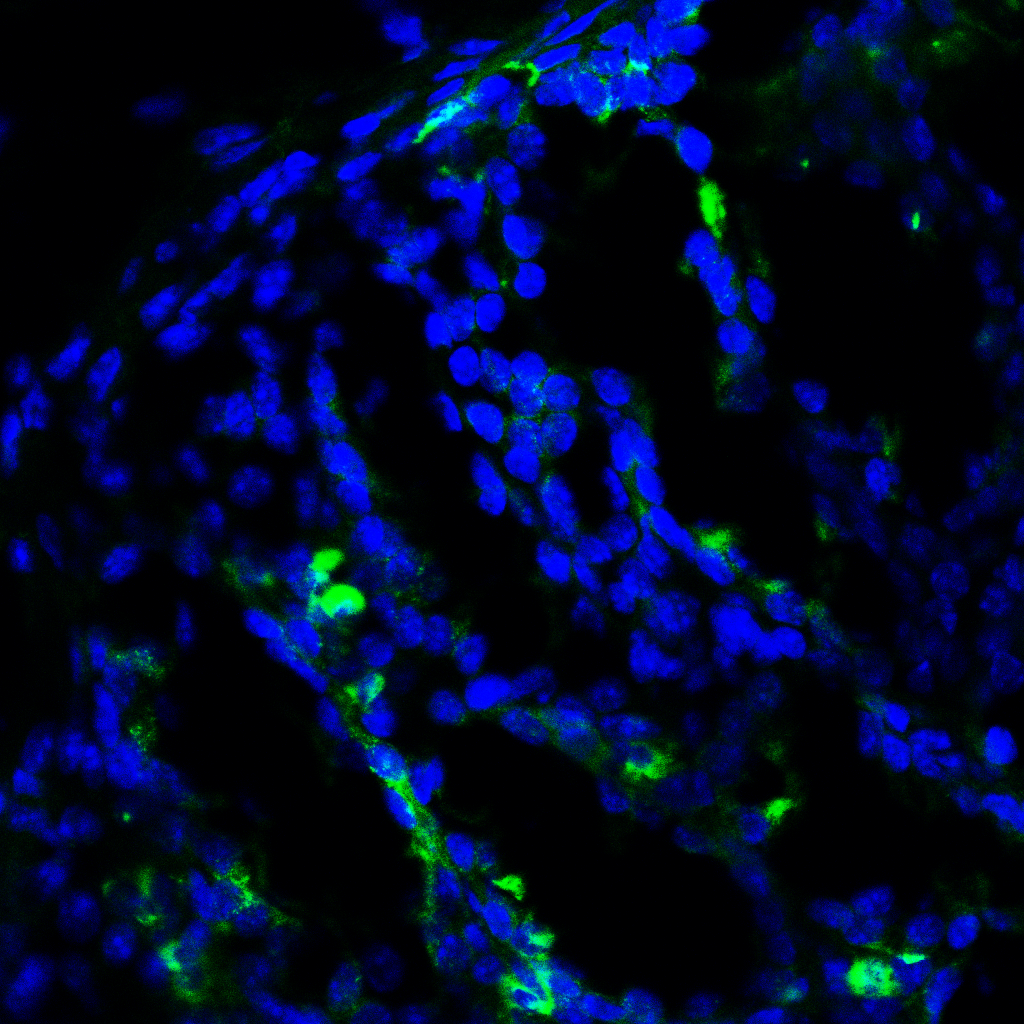

Supplement: Supplementary Figure 2 — (A) Masson’s trichrome staining showed the collagen deposition in each group, no significant difference between BMP4 recombinant protein and BMP4 antibody treatment. (B) Masson’s trichrome staining showed no significant difference of collagen deposition between Id3–/– aged mice and wild type aged mice. Scale bar = 50 μm. [file Data_Sheet_4.ZIP › fig2/5day lgr5.tif]

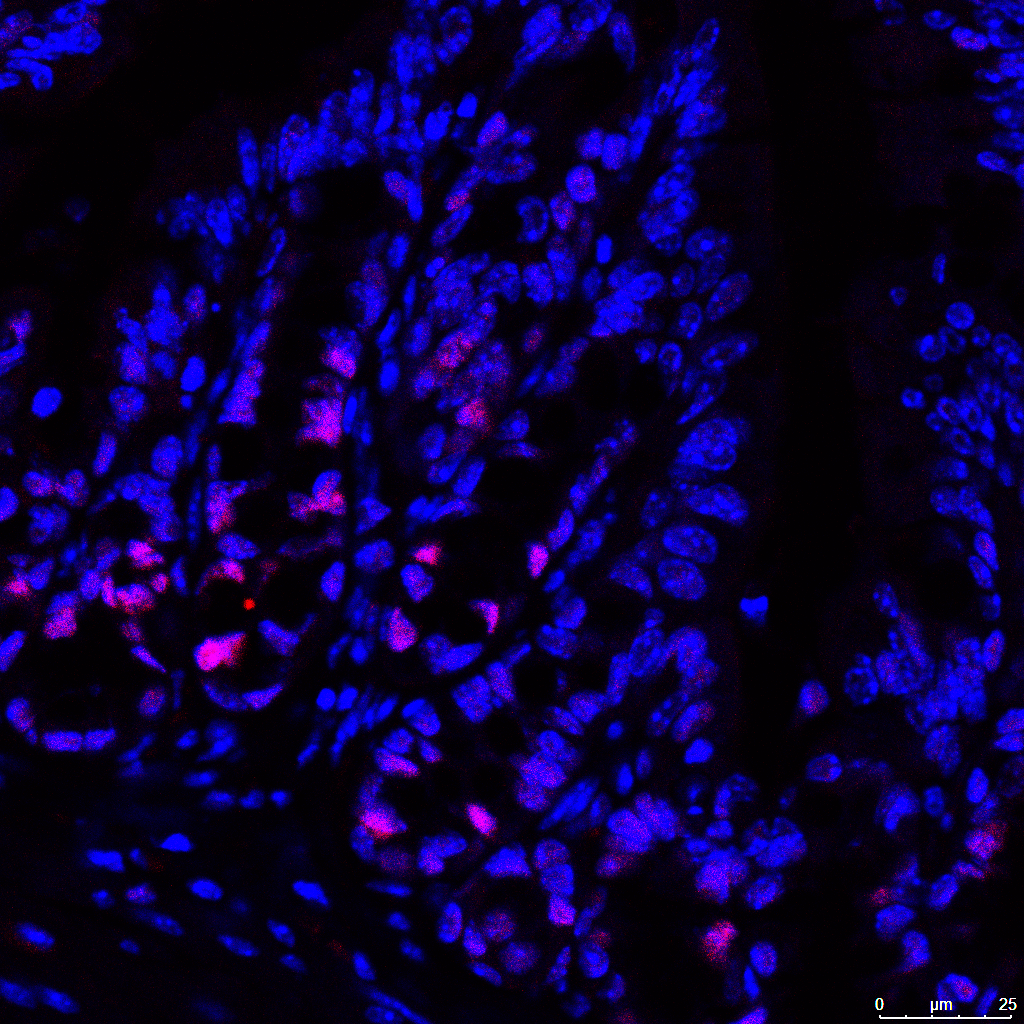

Supplement: Supplementary Figure 2 — (A) Masson’s trichrome staining showed the collagen deposition in each group, no significant difference between BMP4 recombinant protein and BMP4 antibody treatment. (B) Masson’s trichrome staining showed no significant difference of collagen deposition between Id3–/– aged mice and wild type aged mice. Scale bar = 50 μm. [file Data_Sheet_4.ZIP › fig2/7 day.tif]

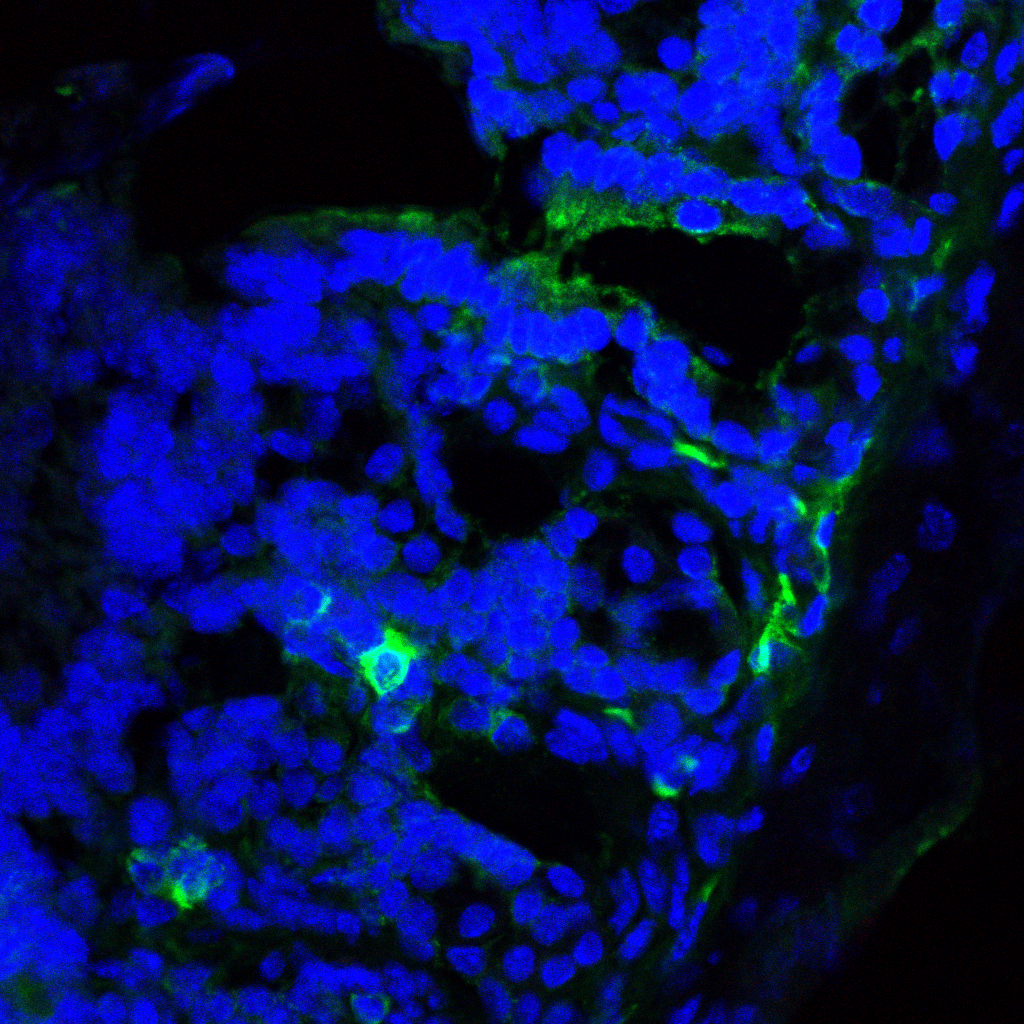

Supplement: Supplementary Figure 2 — (A) Masson’s trichrome staining showed the collagen deposition in each group, no significant difference between BMP4 recombinant protein and BMP4 antibody treatment. (B) Masson’s trichrome staining showed no significant difference of collagen deposition between Id3–/– aged mice and wild type aged mice. Scale bar = 50 μm. [file Data_Sheet_4.ZIP › fig2/7day lgr5.tif]

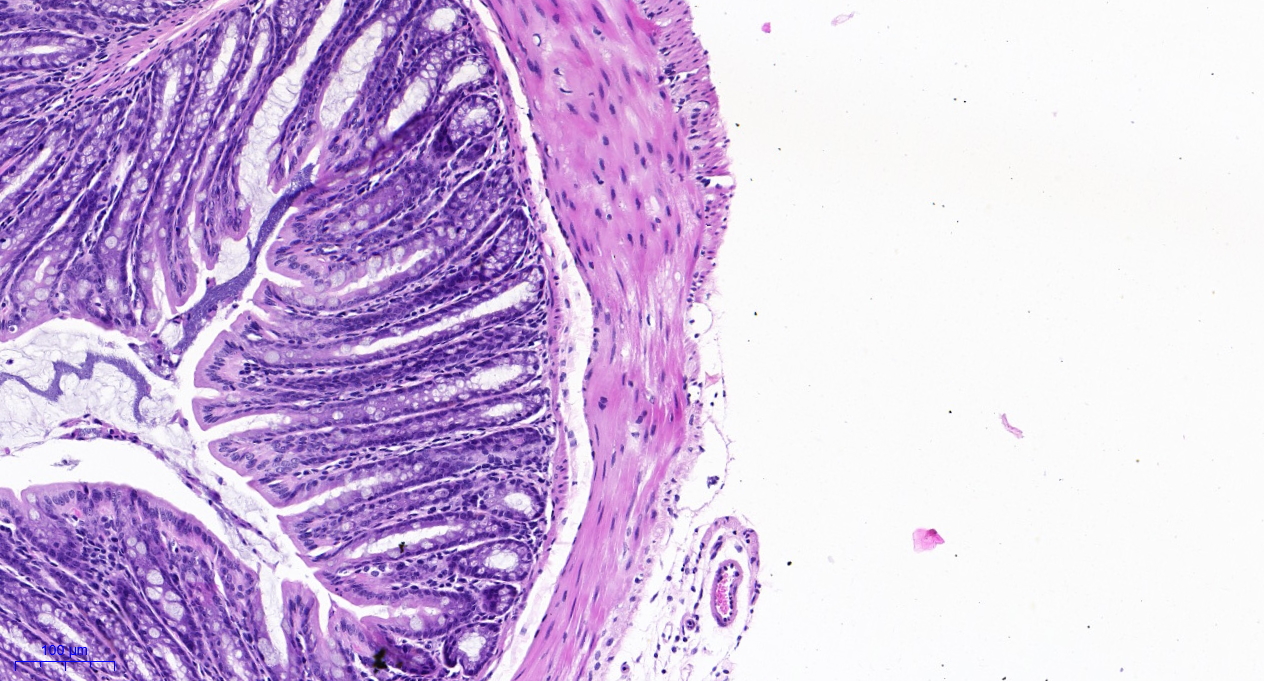

Supplement: Supplementary file 5 [file Data_Sheet_5.ZIP › fig4/1M01_10.0x.jpg]

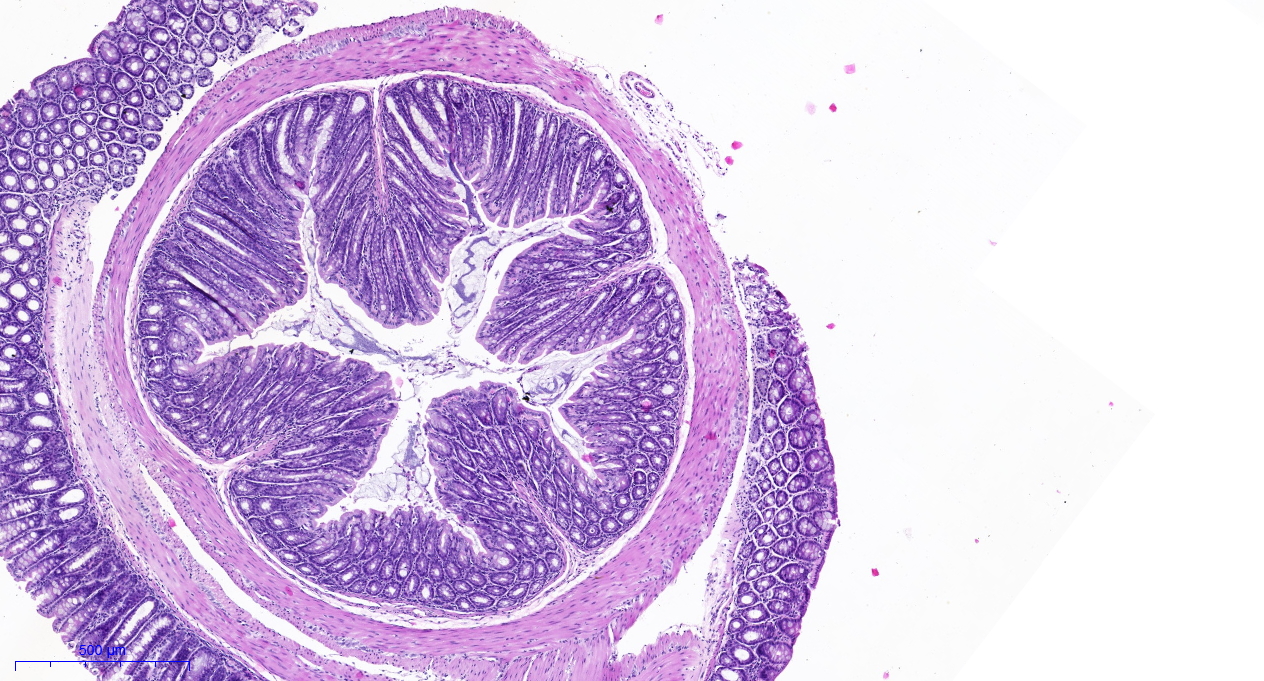

Supplement: Supplementary file 5 [file Data_Sheet_5.ZIP › fig4/1M01_3.5x.jpg]

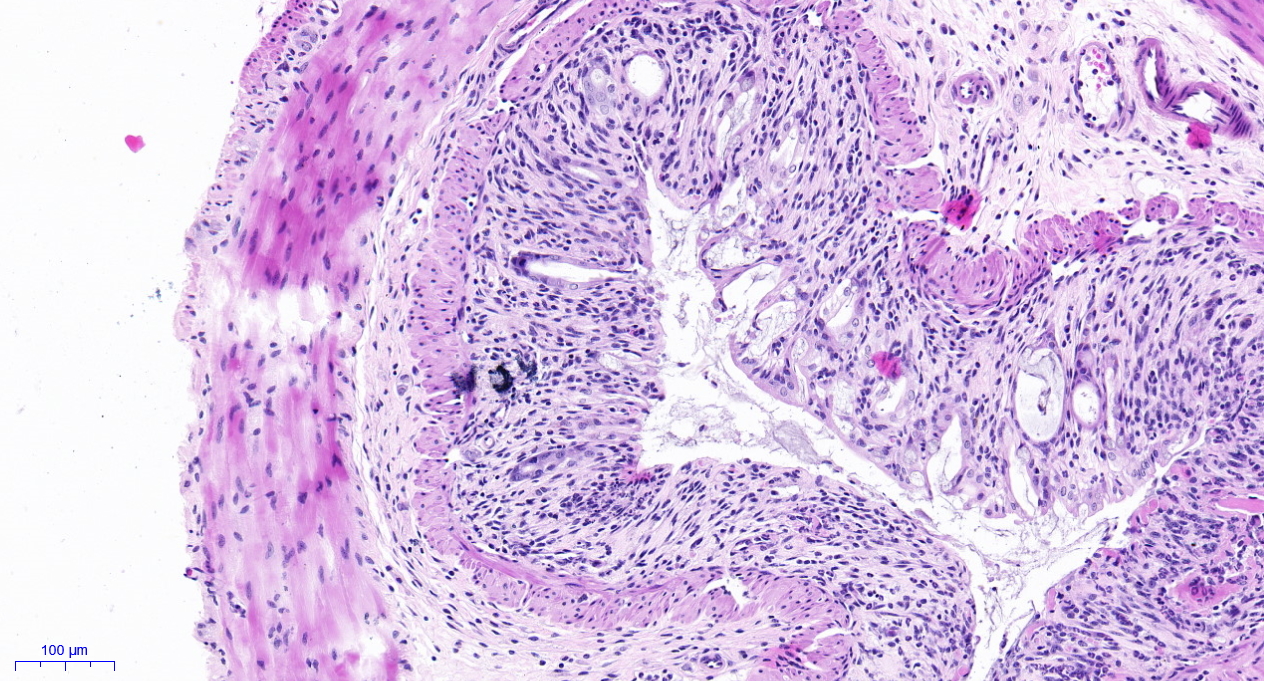

Supplement: Supplementary file 5 [file Data_Sheet_5.ZIP › fig4/7day 10.0x-1.jpg]

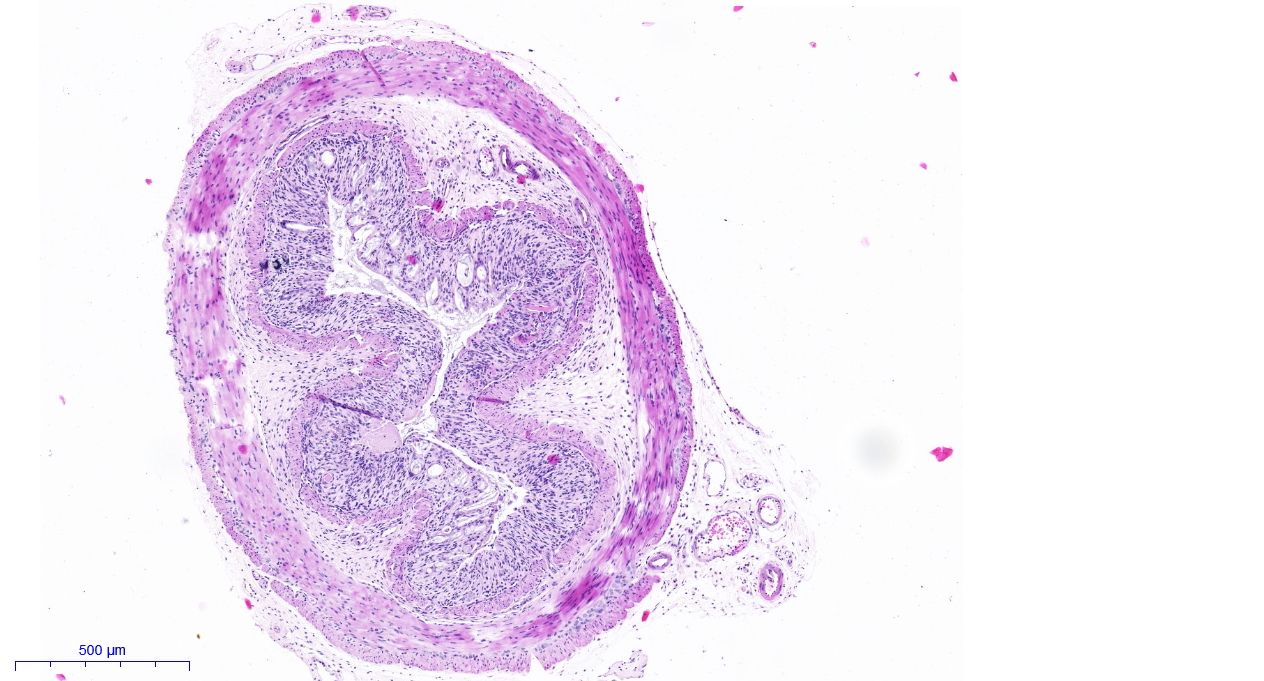

Supplement: Supplementary file 5 [file Data_Sheet_5.ZIP › fig4/7day_3.5x-1.jpg]

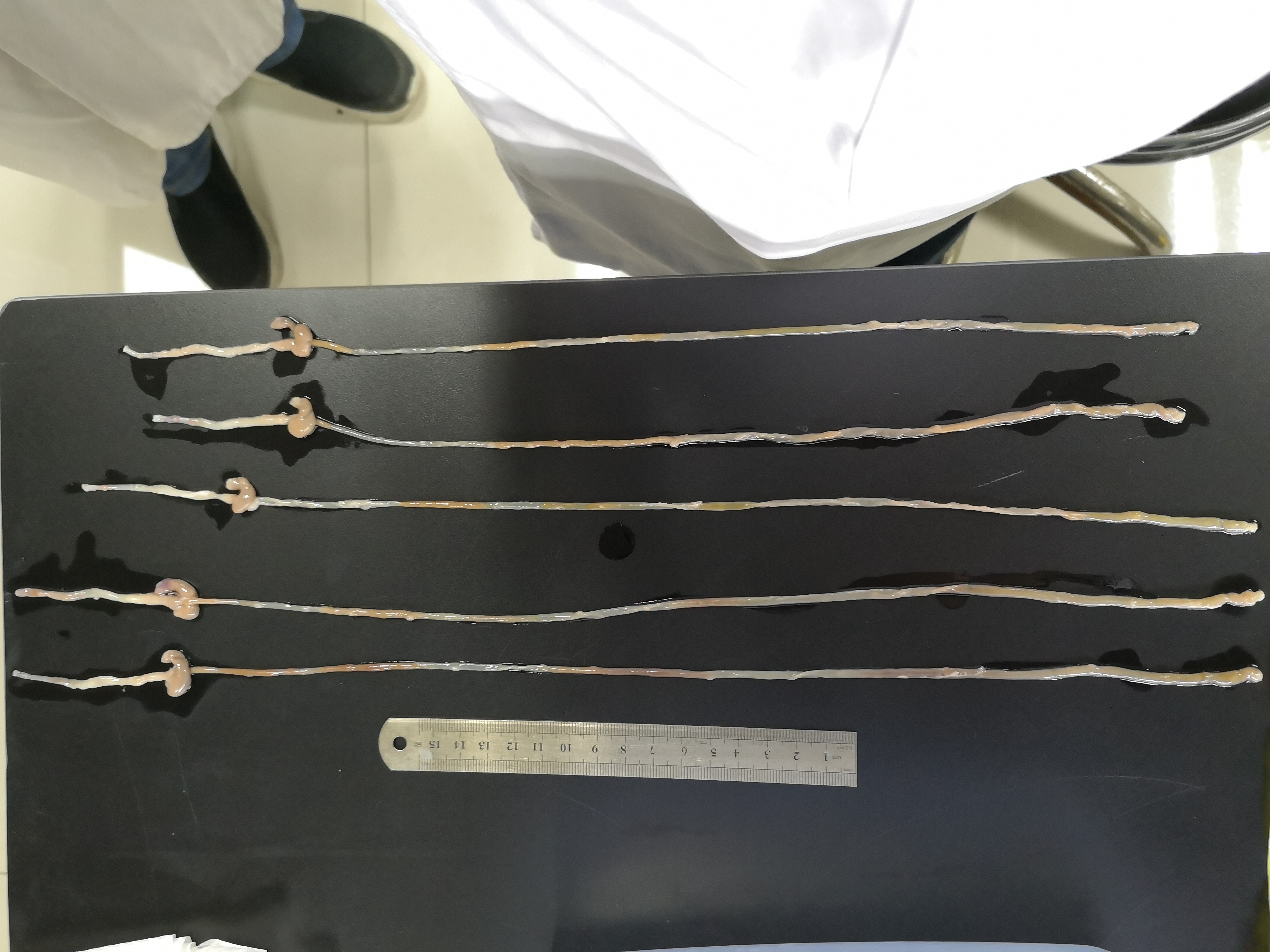

Supplement: Supplementary file 5 [file Data_Sheet_5.ZIP › fig4/blank.jpg]

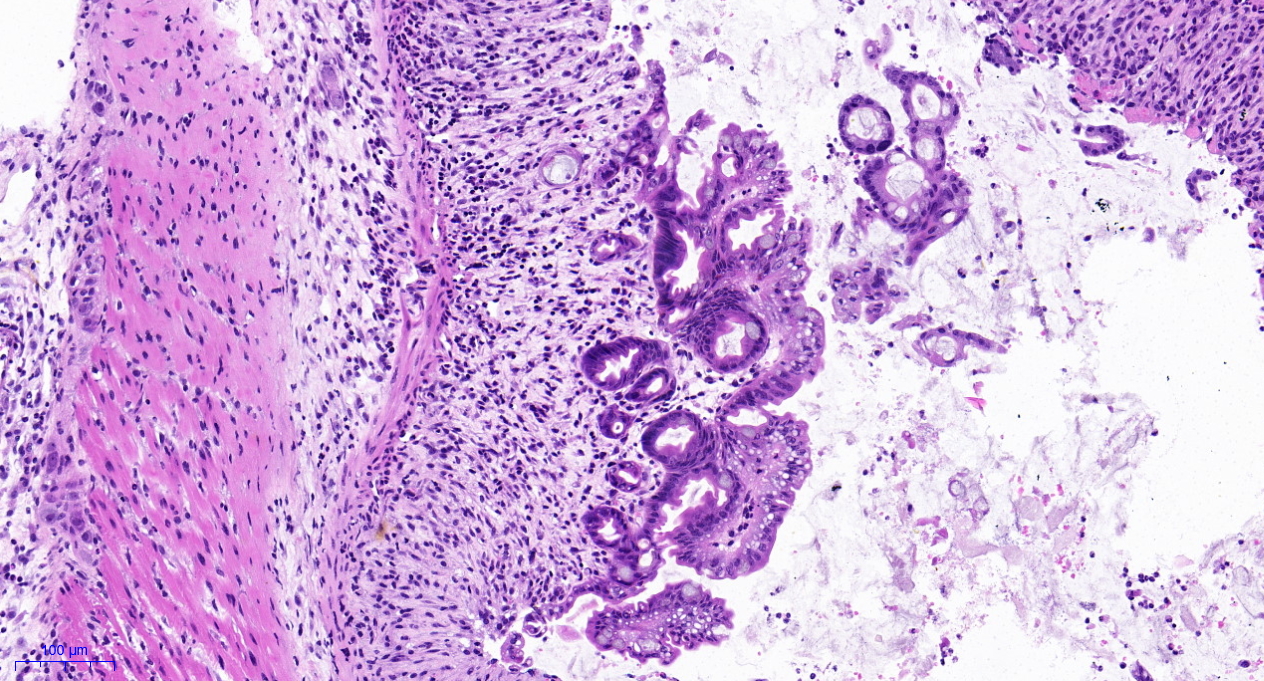

Supplement: Supplementary file 5 [file Data_Sheet_5.ZIP › fig4/bmp4 inhibit_10.0x.jpg]

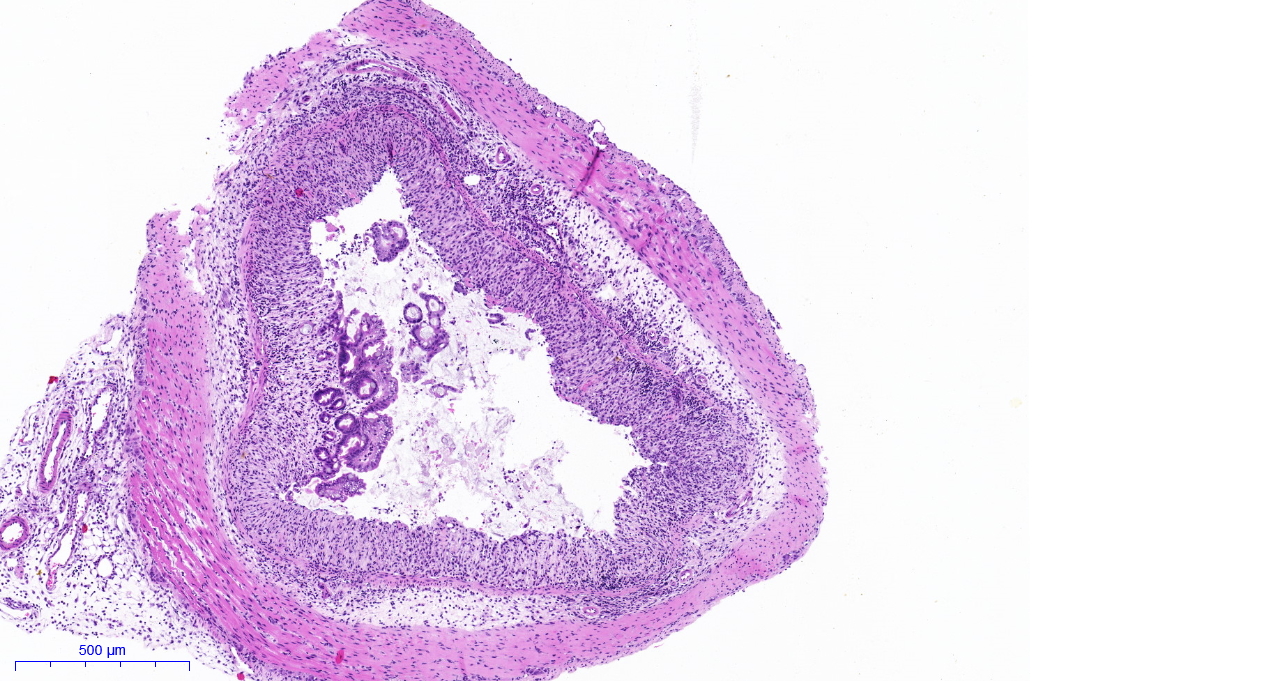

Supplement: Supplementary file 5 [file Data_Sheet_5.ZIP › fig4/bmp4 inhibitor_3.5x.jpg]

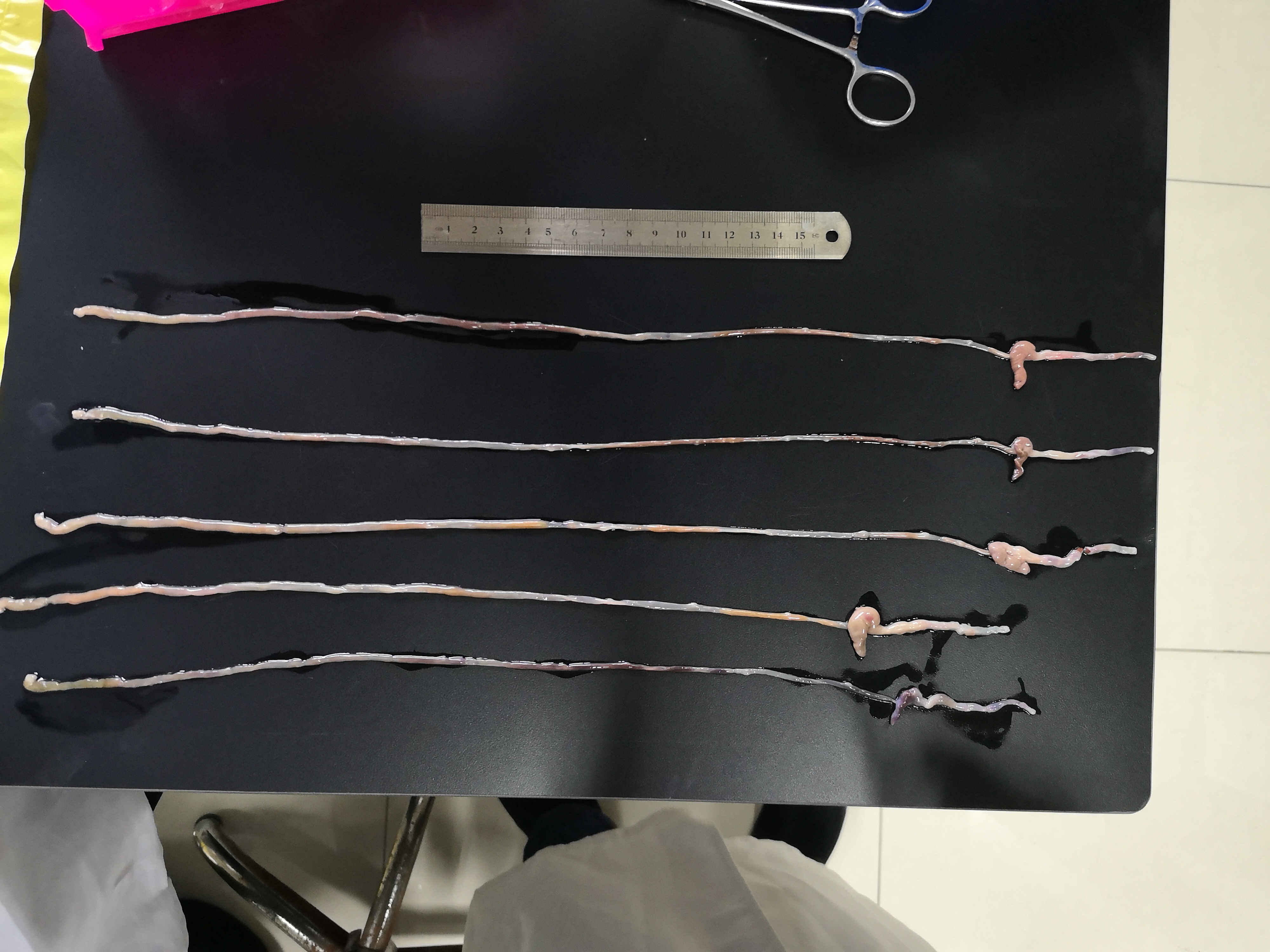

Supplement: Supplementary file 5 [file Data_Sheet_5.ZIP › fig4/bmp4.jpg]

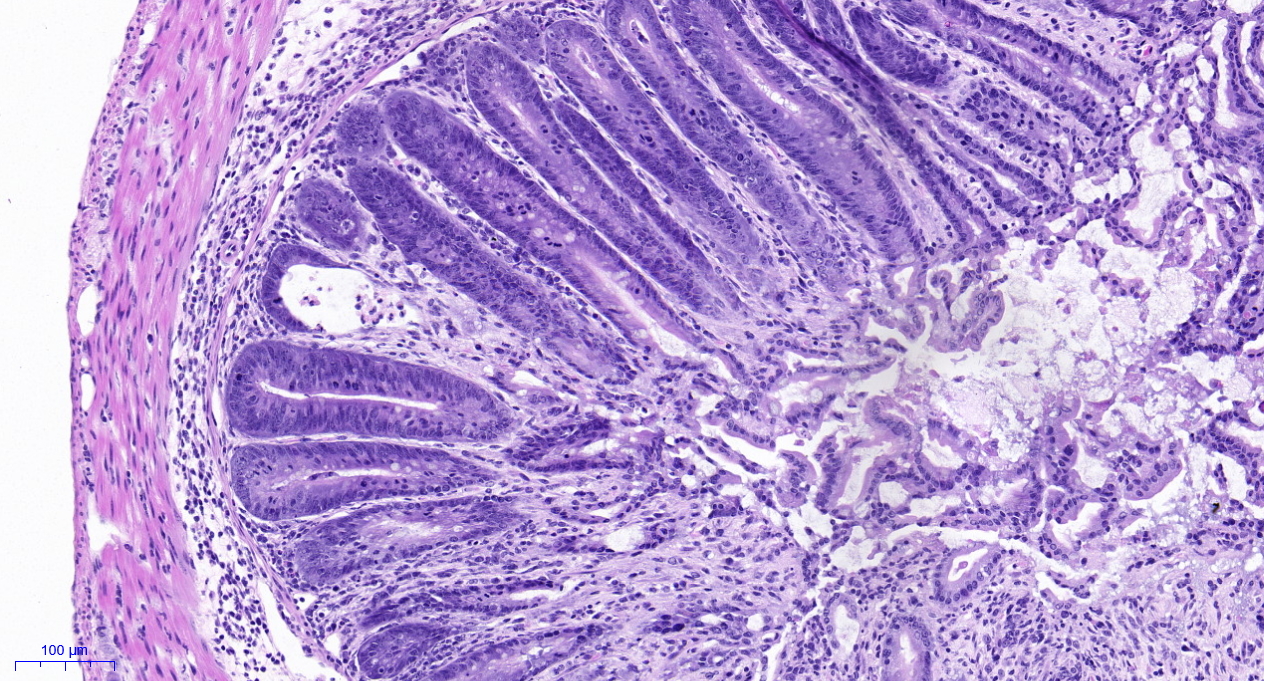

Supplement: Supplementary file 5 [file Data_Sheet_5.ZIP › fig4/bmp4_10.0x.jpg]

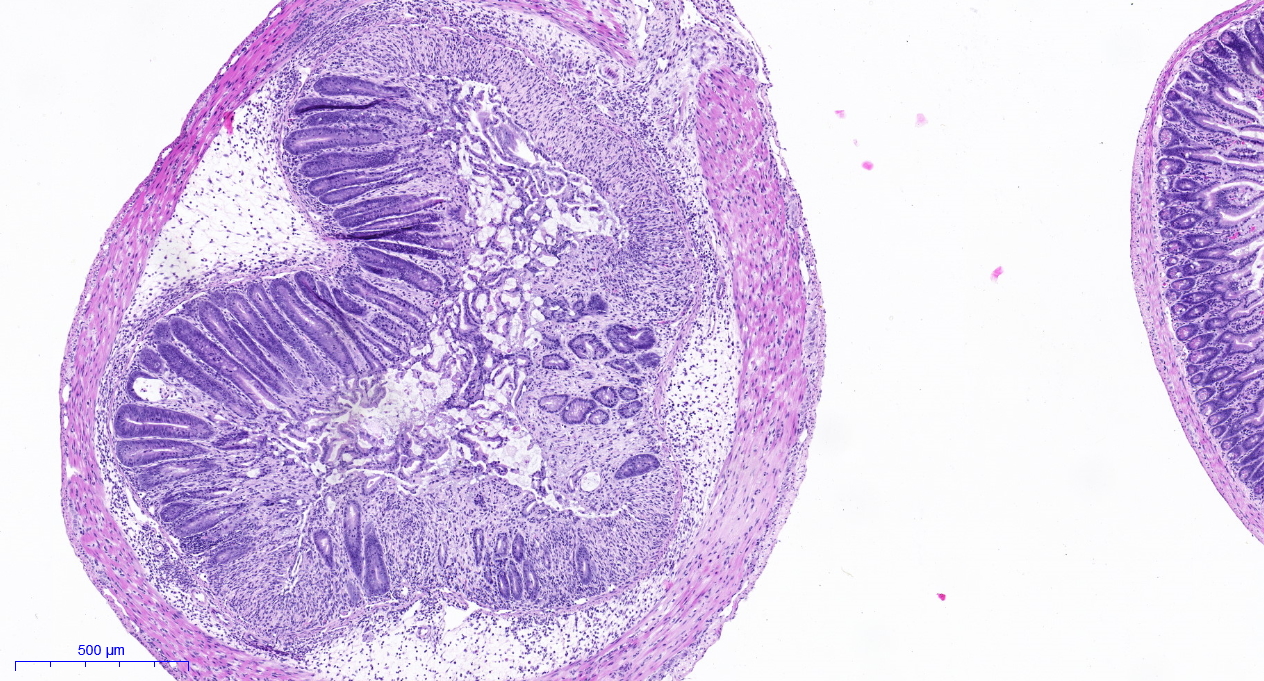

Supplement: Supplementary file 5 [file Data_Sheet_5.ZIP › fig4/bmp4_3.5x.jpg]

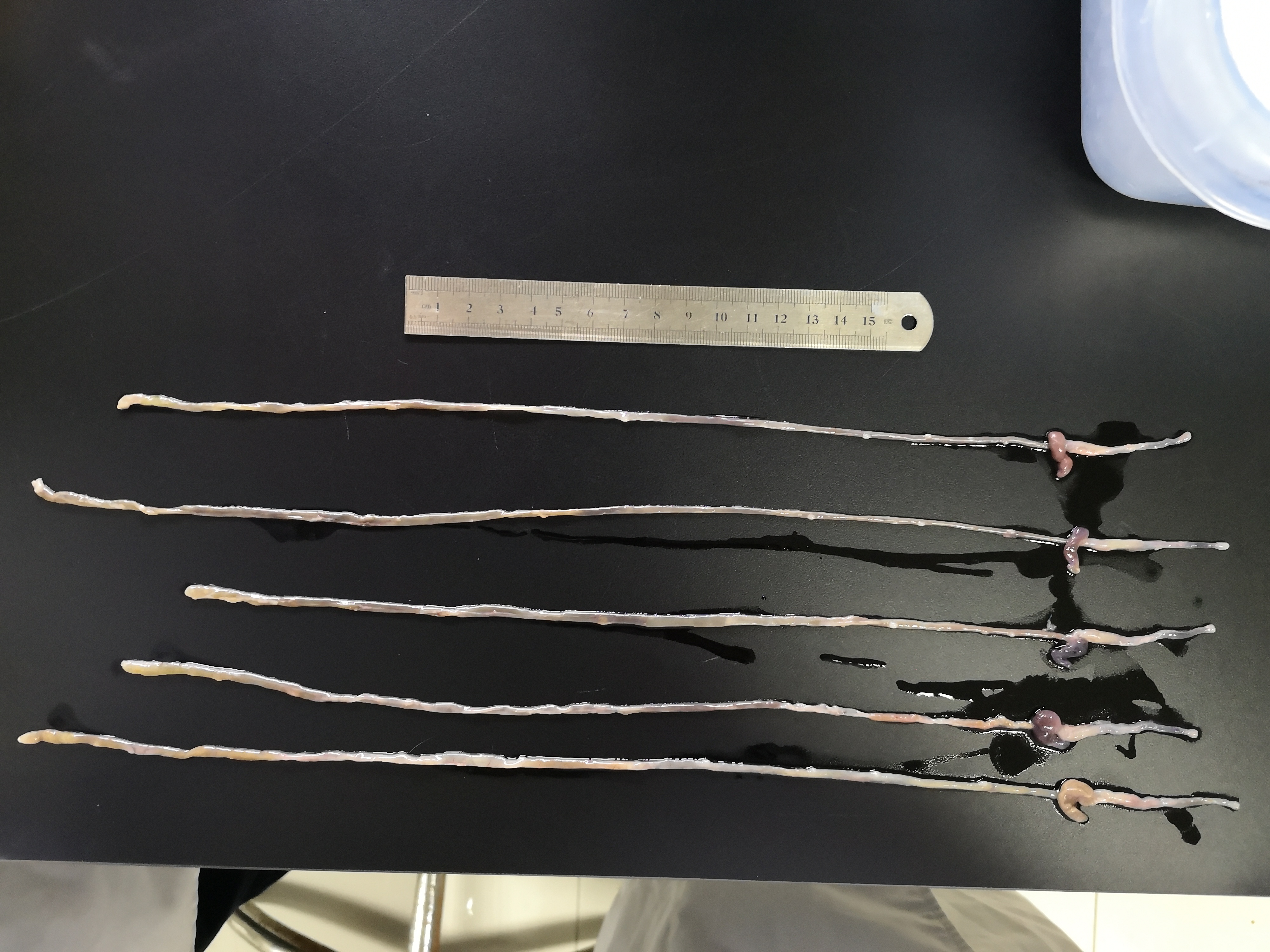

Supplement: Supplementary file 5 [file Data_Sheet_5.ZIP › fig4/bmpinhi.jpg]

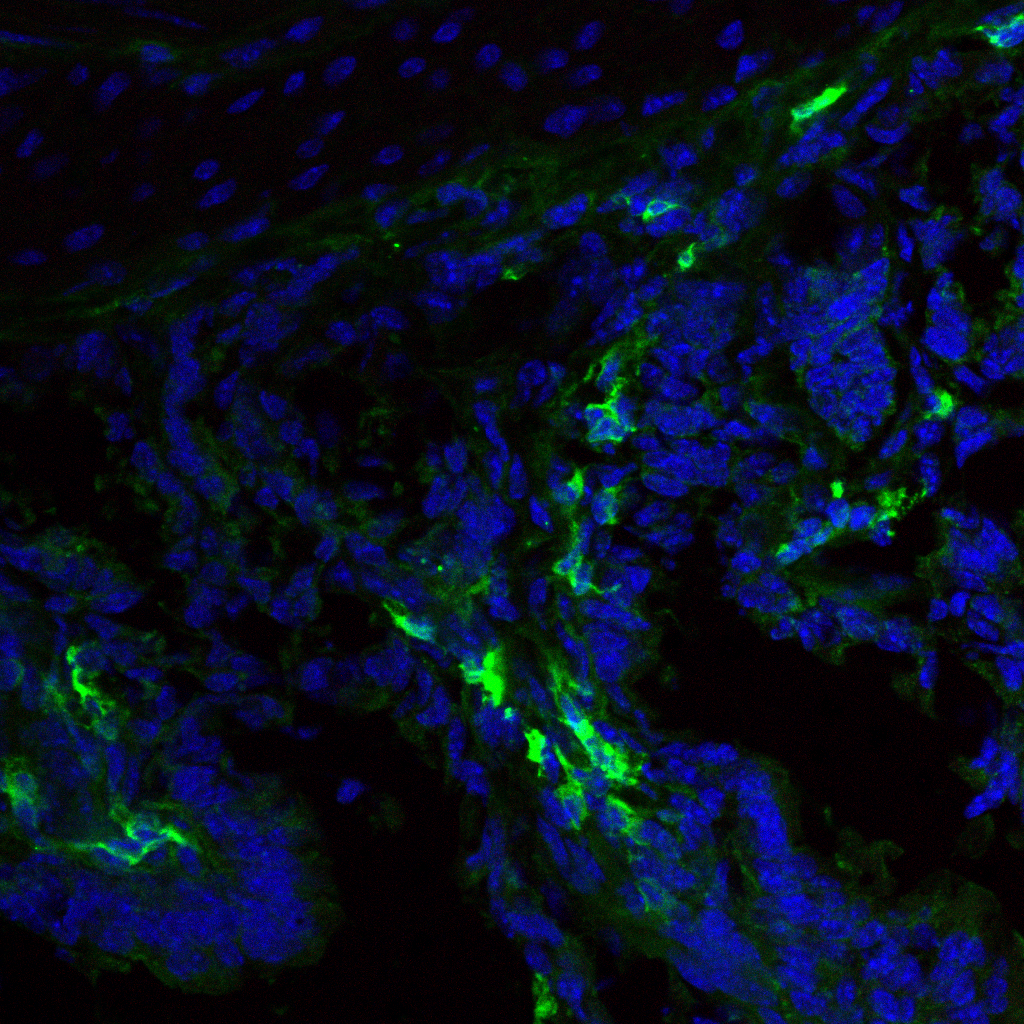

Supplement: Supplementary file 6 [file Data_Sheet_6.ZIP › fig5/0day Image005.tif]

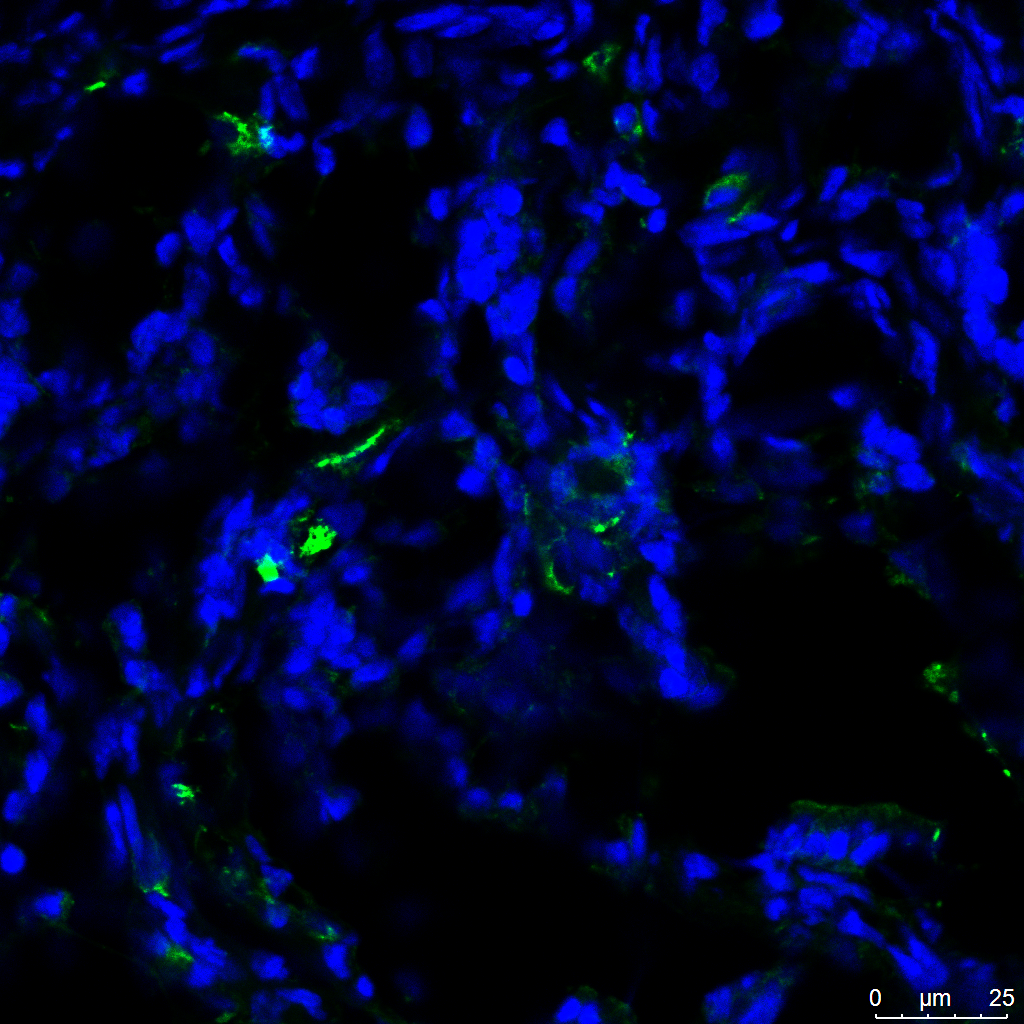

Supplement: Supplementary file 6 [file Data_Sheet_6.ZIP › fig5/DSS LGR5.tif]

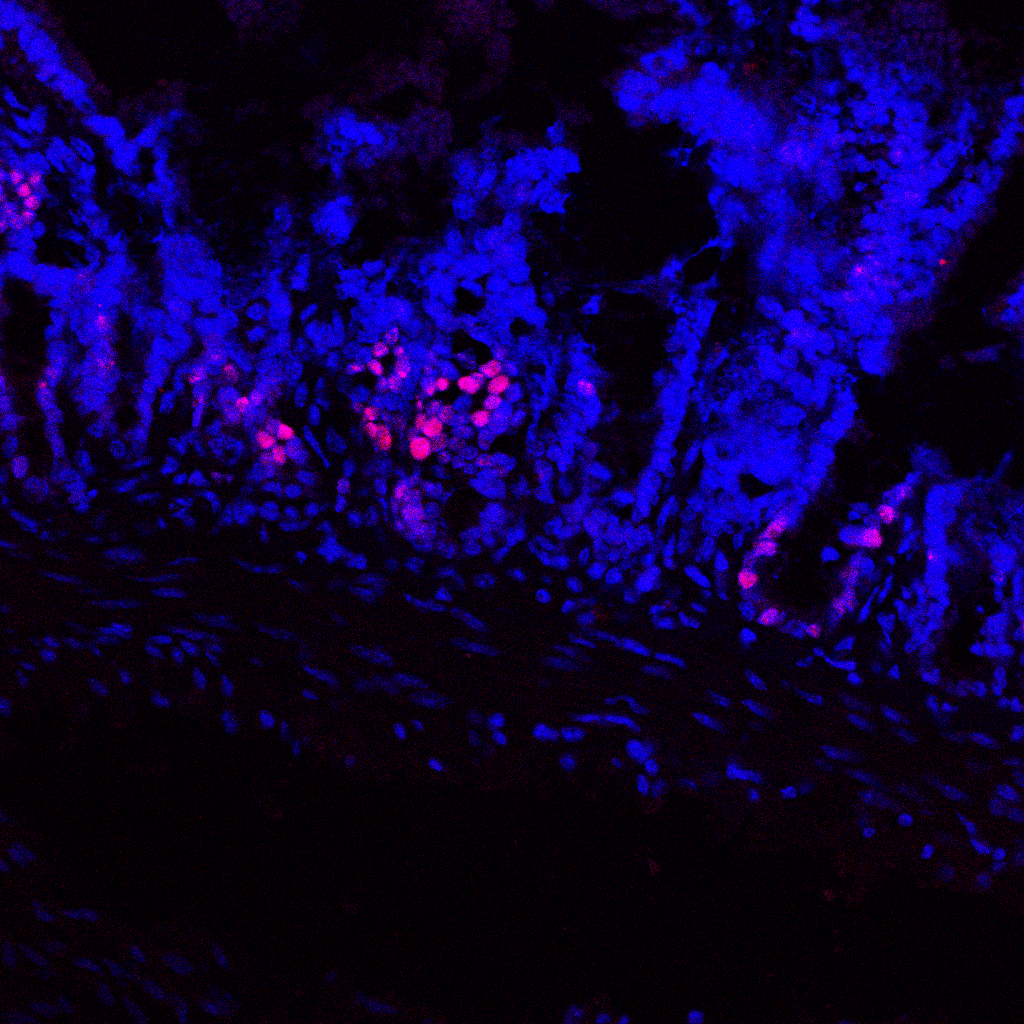

Supplement: Supplementary file 6 [file Data_Sheet_6.ZIP › fig5/DSS ki67.tif]

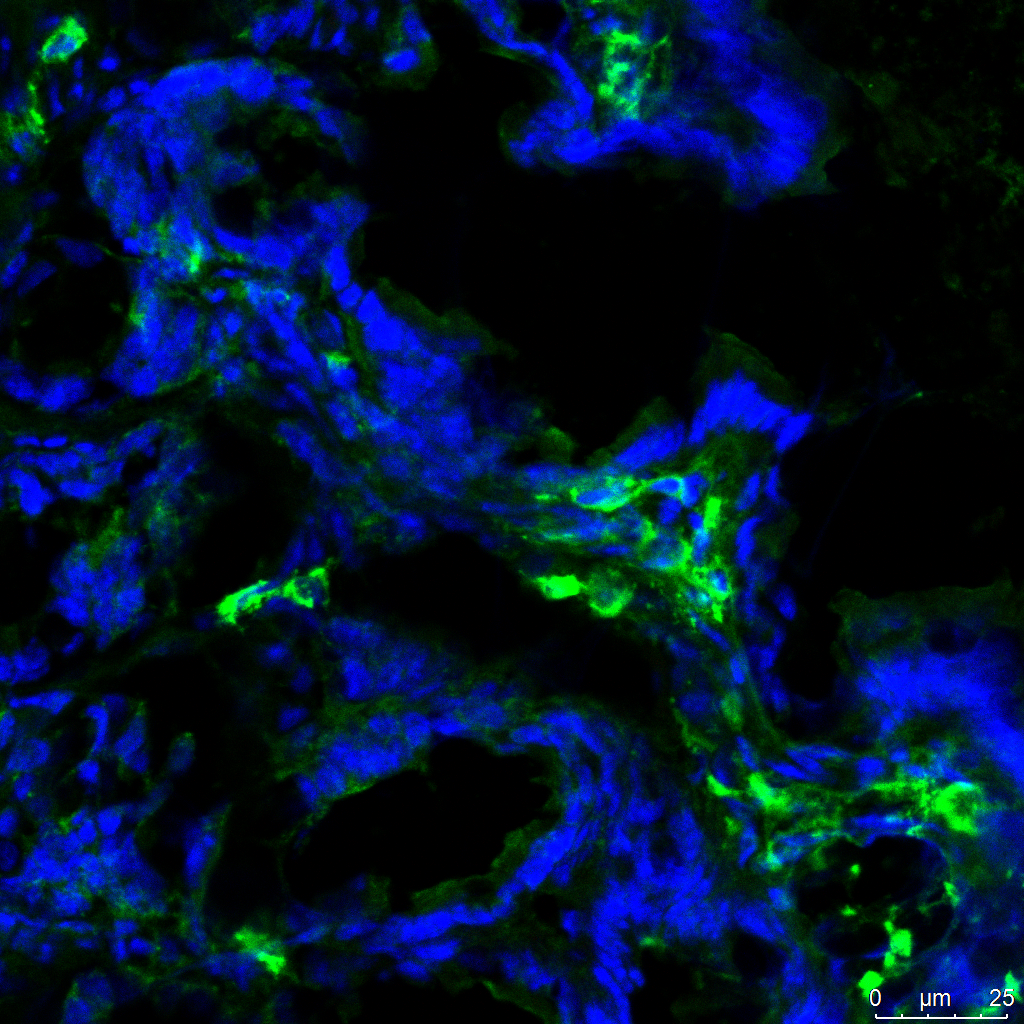

Supplement: Supplementary file 6 [file Data_Sheet_6.ZIP › fig5/LGR5 bmp4.tif]

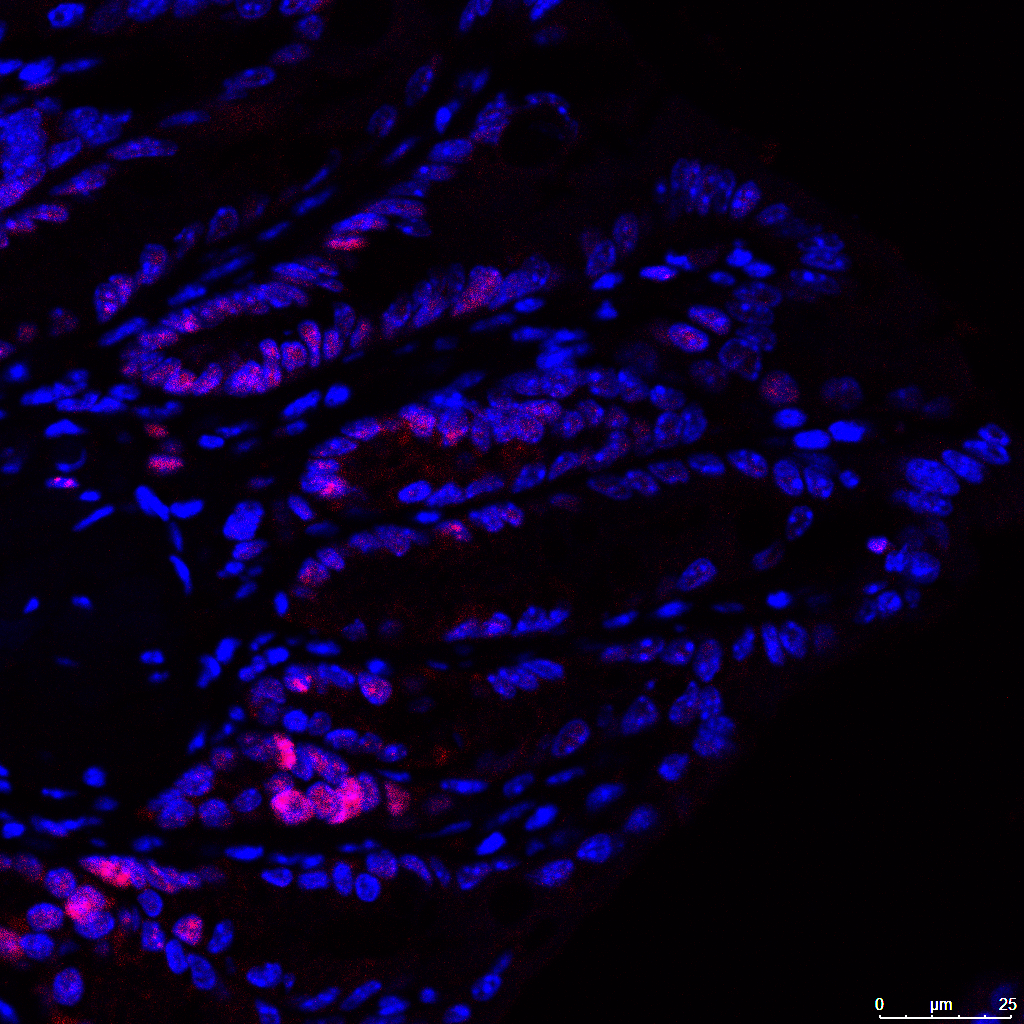

Supplement: Supplementary file 6 [file Data_Sheet_6.ZIP › fig5/blank ki67.tif]

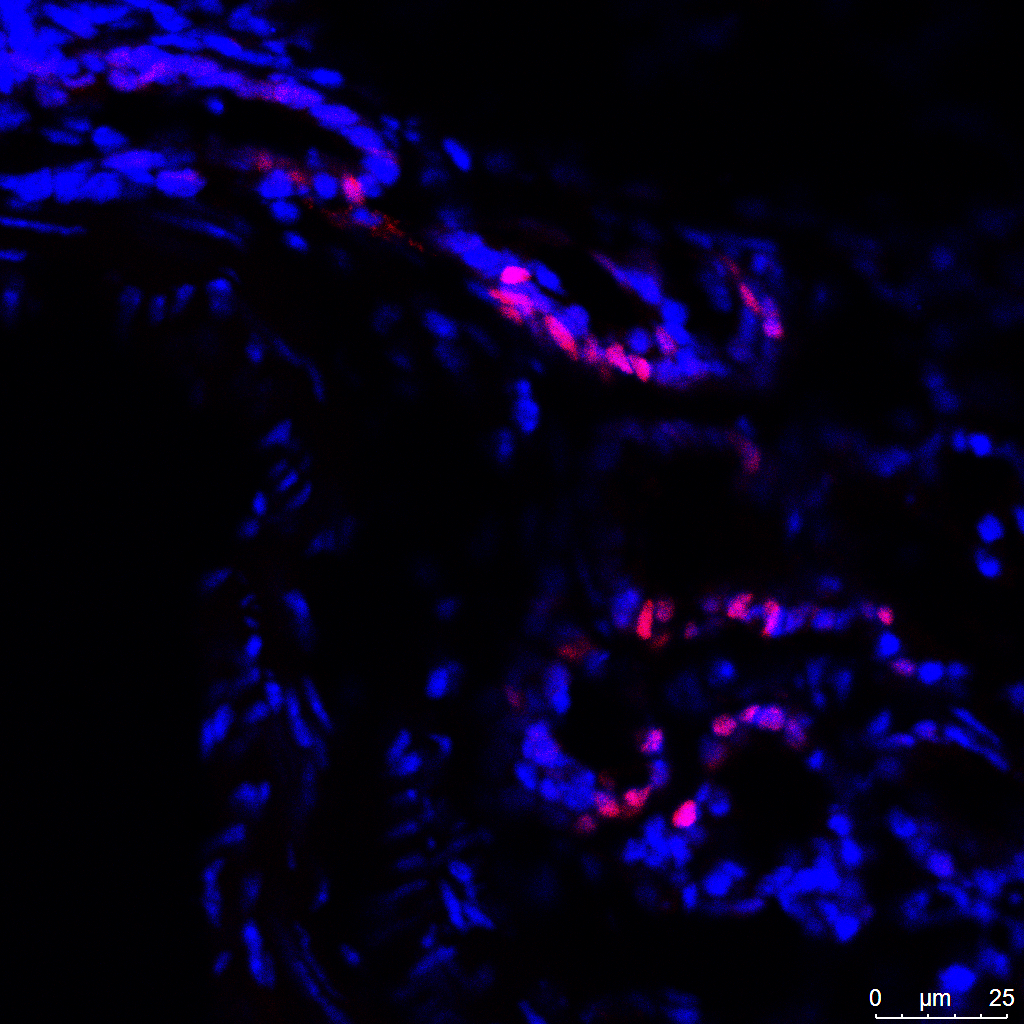

Supplement: Supplementary file 6 [file Data_Sheet_6.ZIP › fig5/bmp4 in ki67.tif]

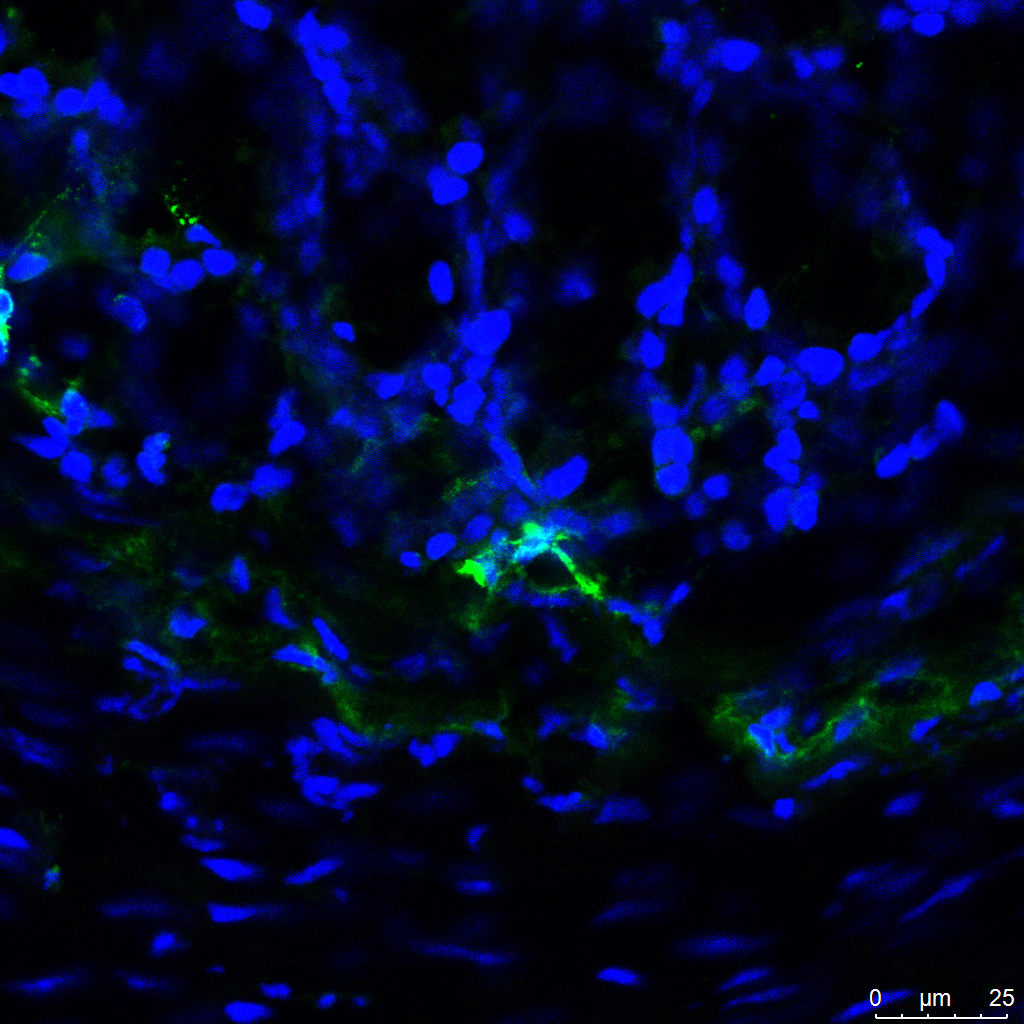

Supplement: Supplementary file 6 [file Data_Sheet_6.ZIP › fig5/bmp4 inhi LGR5.tif]

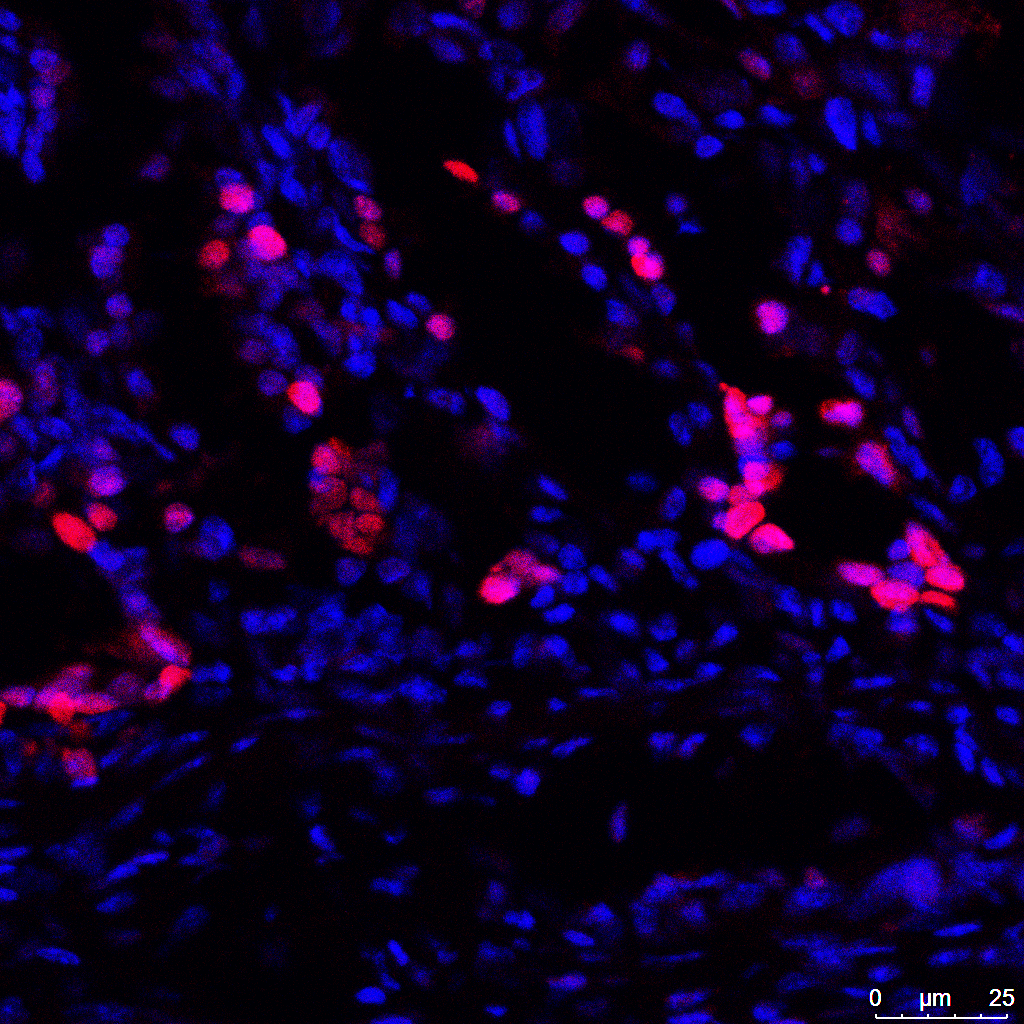

Supplement: Supplementary file 6 [file Data_Sheet_6.ZIP › fig5/bmp4 ki67.tif]

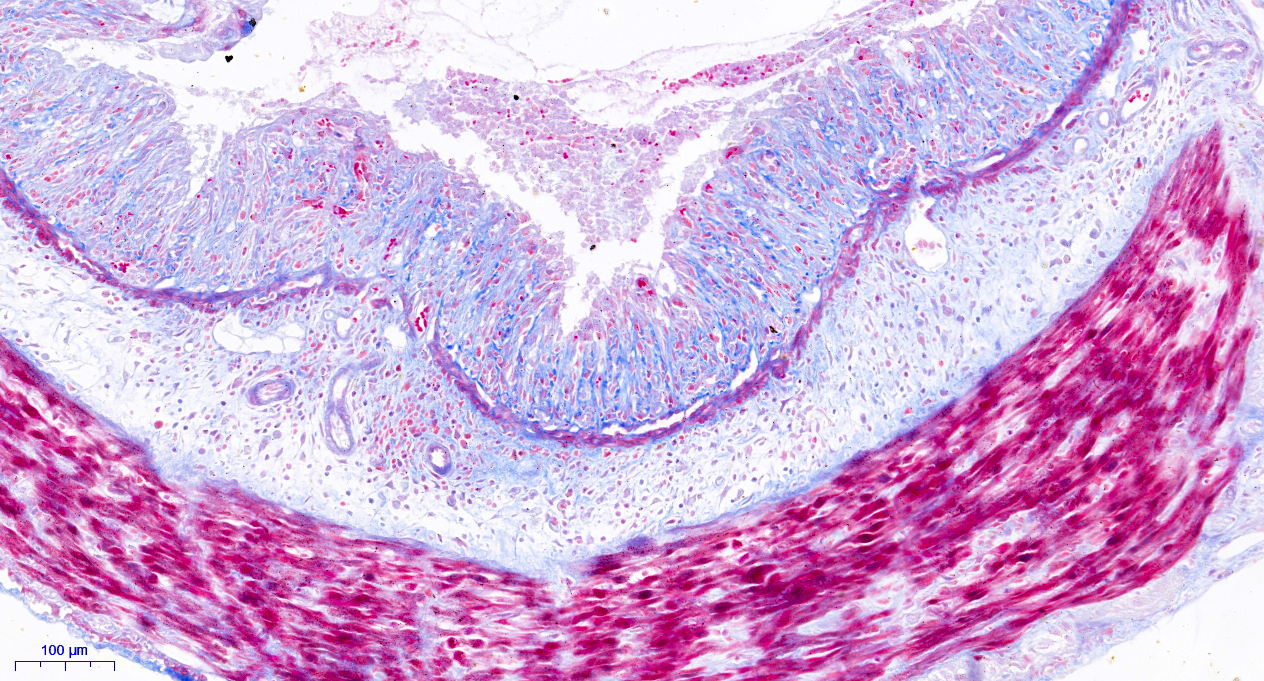

Supplement: Supplementary file 7 [file Data_Sheet_7.ZIP › s-fig/BMP4 in-2_mason 10.0x.jpg]

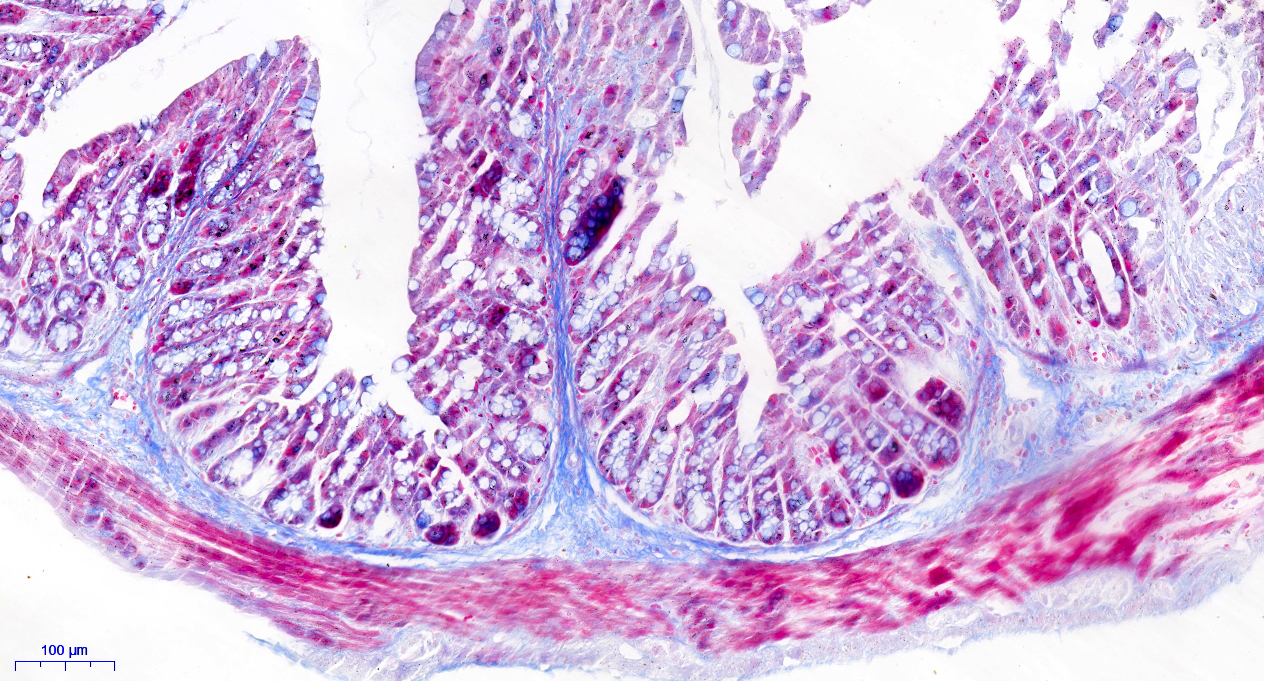

Supplement: Supplementary file 7 [file Data_Sheet_7.ZIP › s-fig/BMP4_mason 10.0x.jpg]

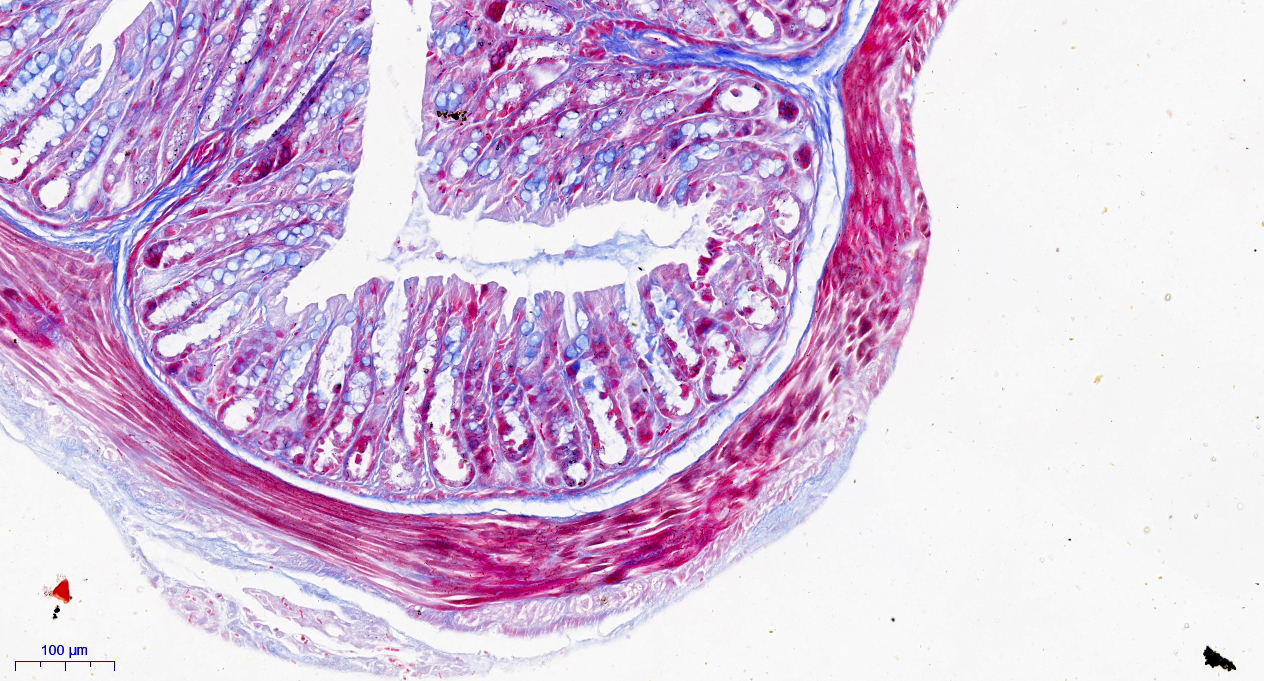

Supplement: Supplementary file 7 [file Data_Sheet_7.ZIP › s-fig/DSS-0day_mason 10.0x.jpg]

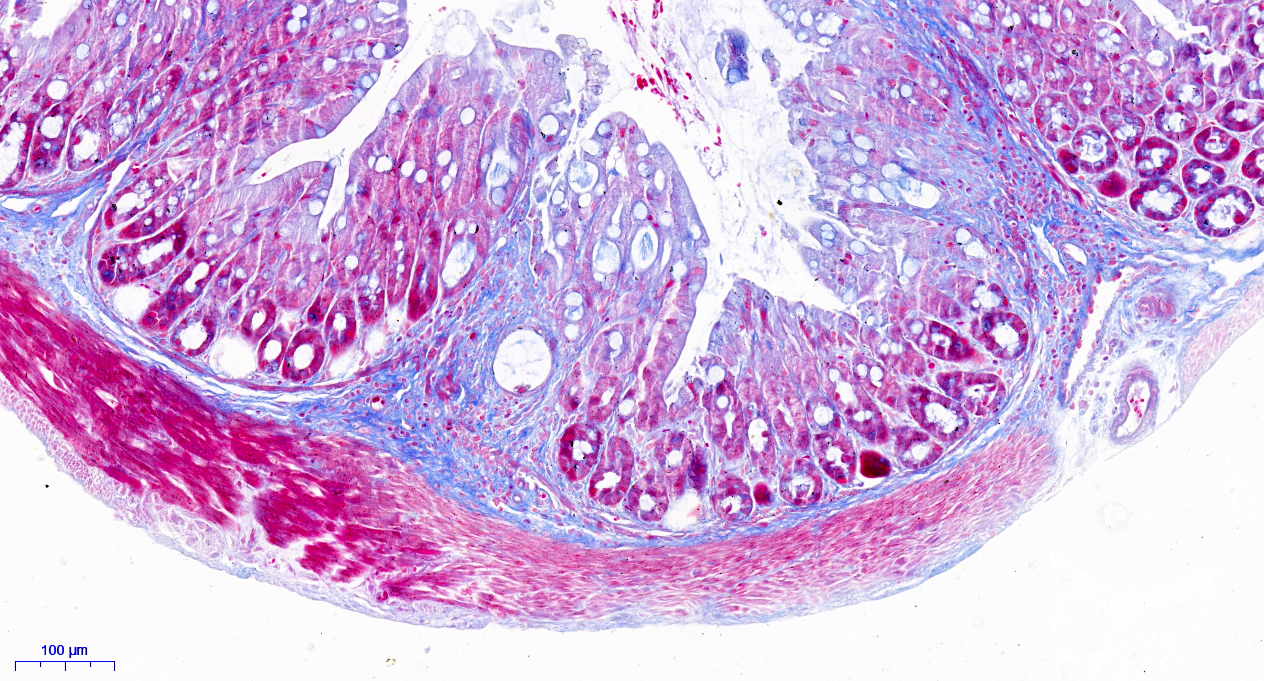

Supplement: Supplementary file 7 [file Data_Sheet_7.ZIP › s-fig/DSS-7day_mason 10.0x.jpg]

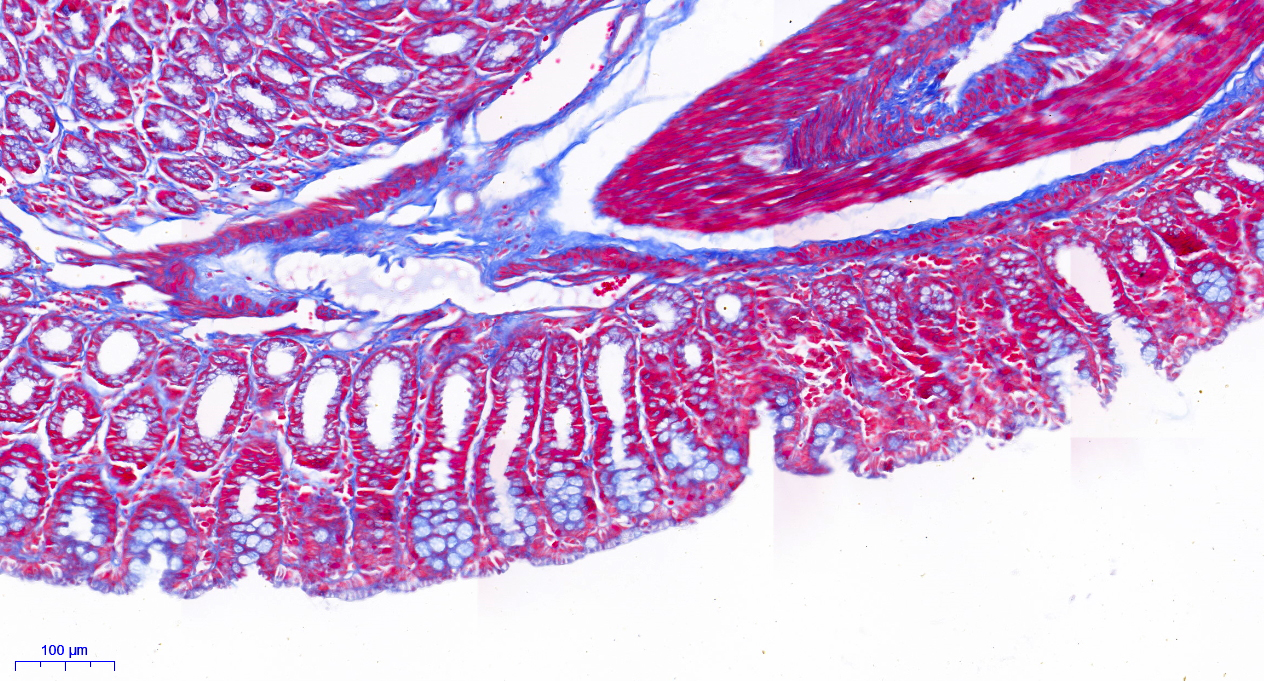

Supplement: Supplementary file 7 [file Data_Sheet_7.ZIP › s-fig/ko mason_10.0x.jpg]

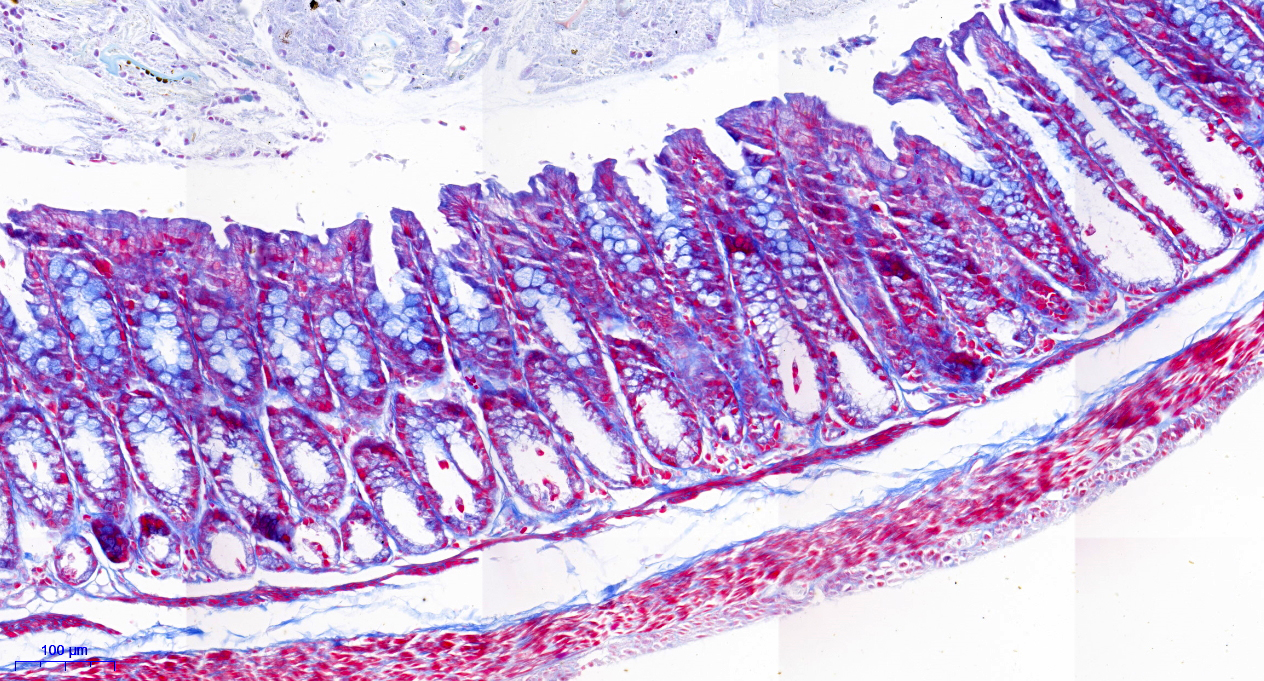

Supplement: Supplementary file 7 [file Data_Sheet_7.ZIP › s-fig/wt masson_10.0x.jpg]

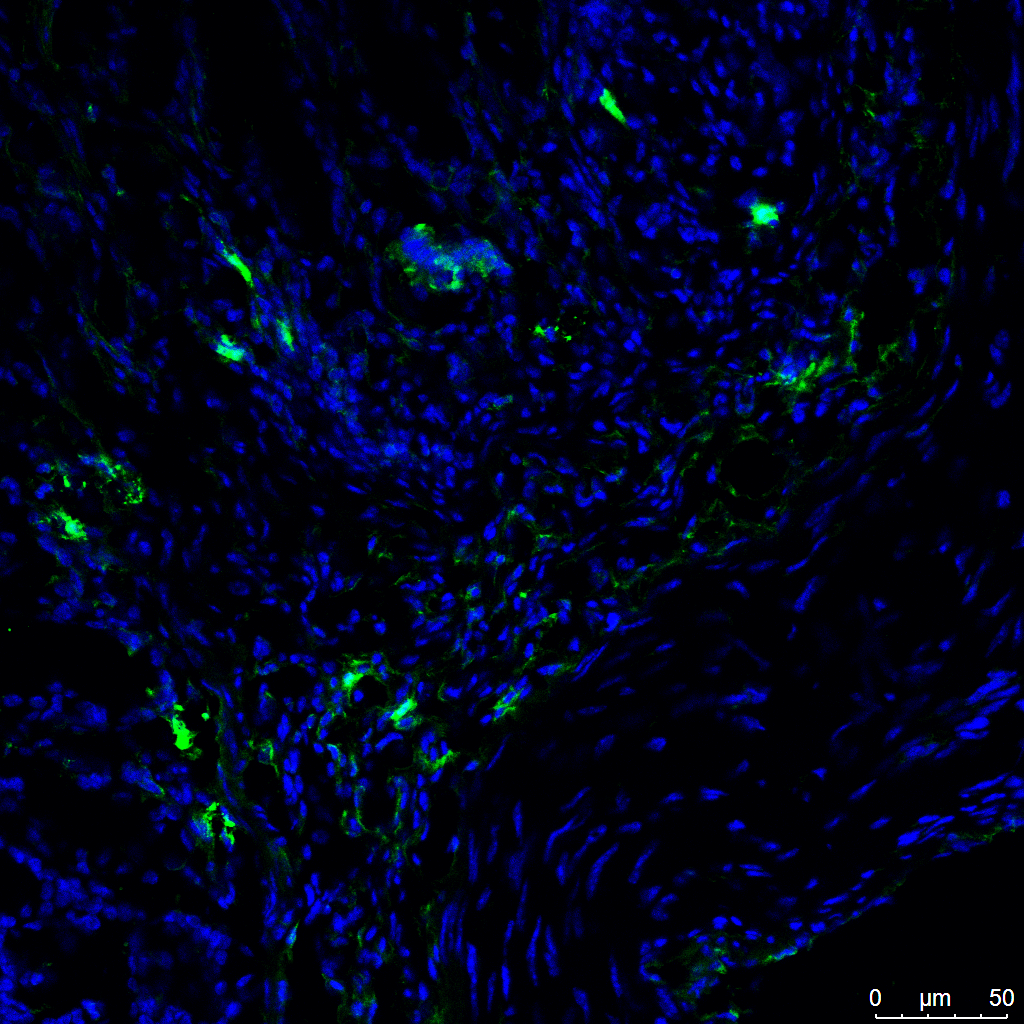

Supplement: Supplementary file 8 [file Data_Sheet_8.ZIP › fig6 raw data-1/BMP4 id3_ch02.tif]

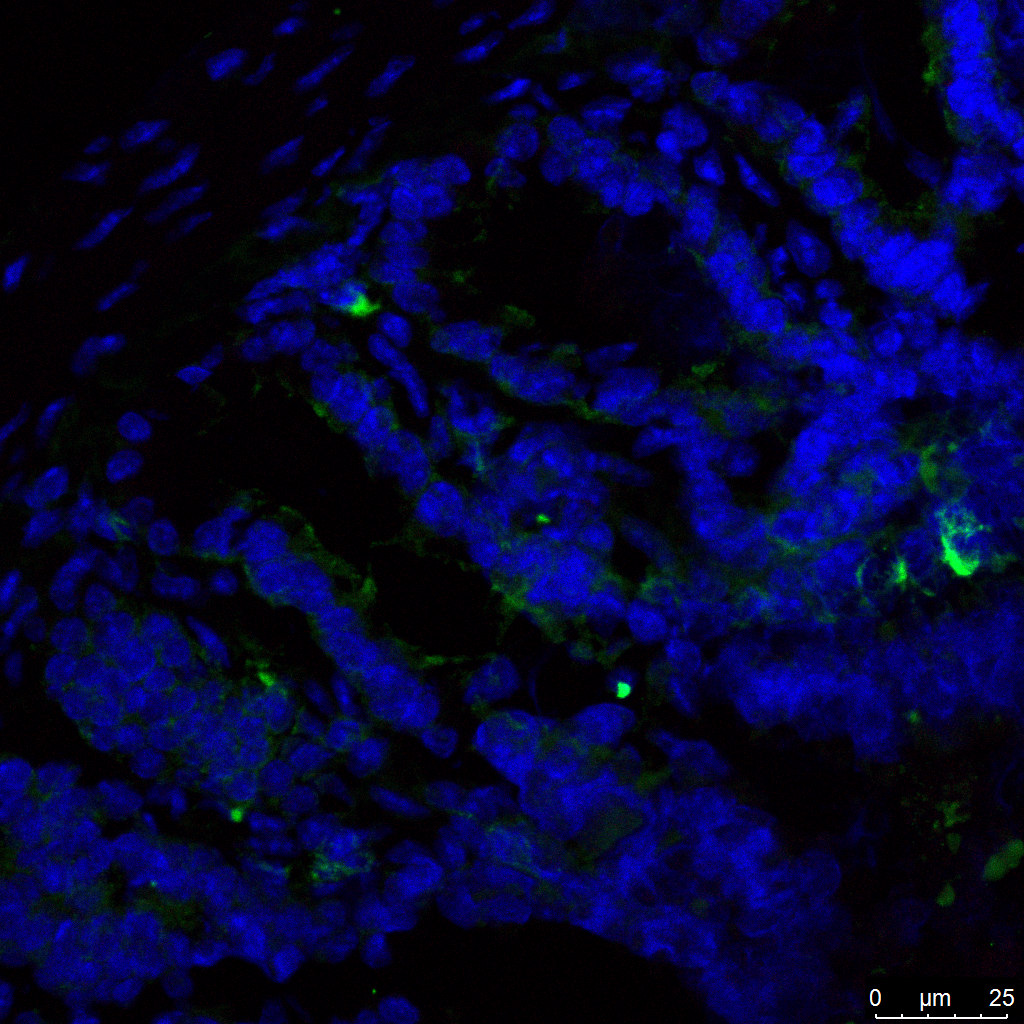

Supplement: Supplementary file 8 [file Data_Sheet_8.ZIP › fig6 raw data-1/DSS id3_Image005.tif]

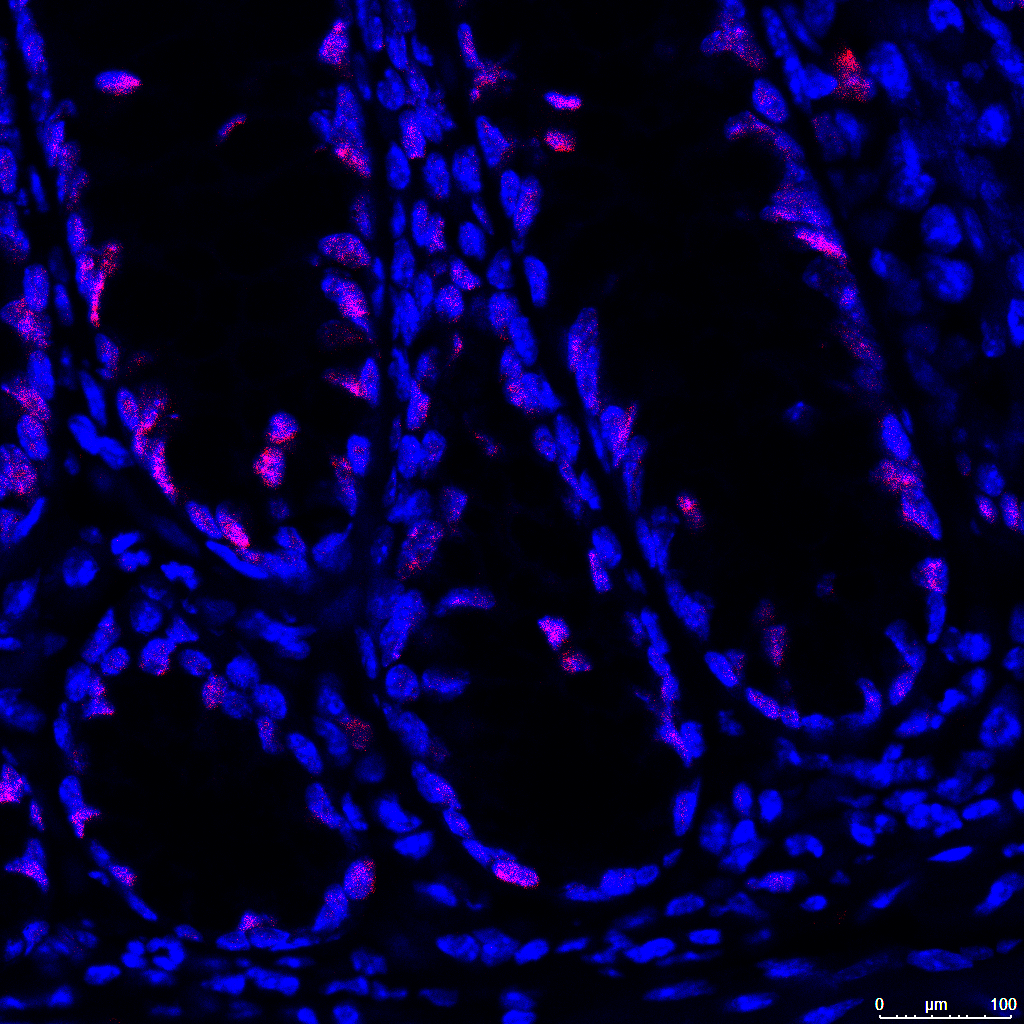

Supplement: Supplementary file 8 [file Data_Sheet_8.ZIP › fig6 raw data-1/Image ko ki67.tif]

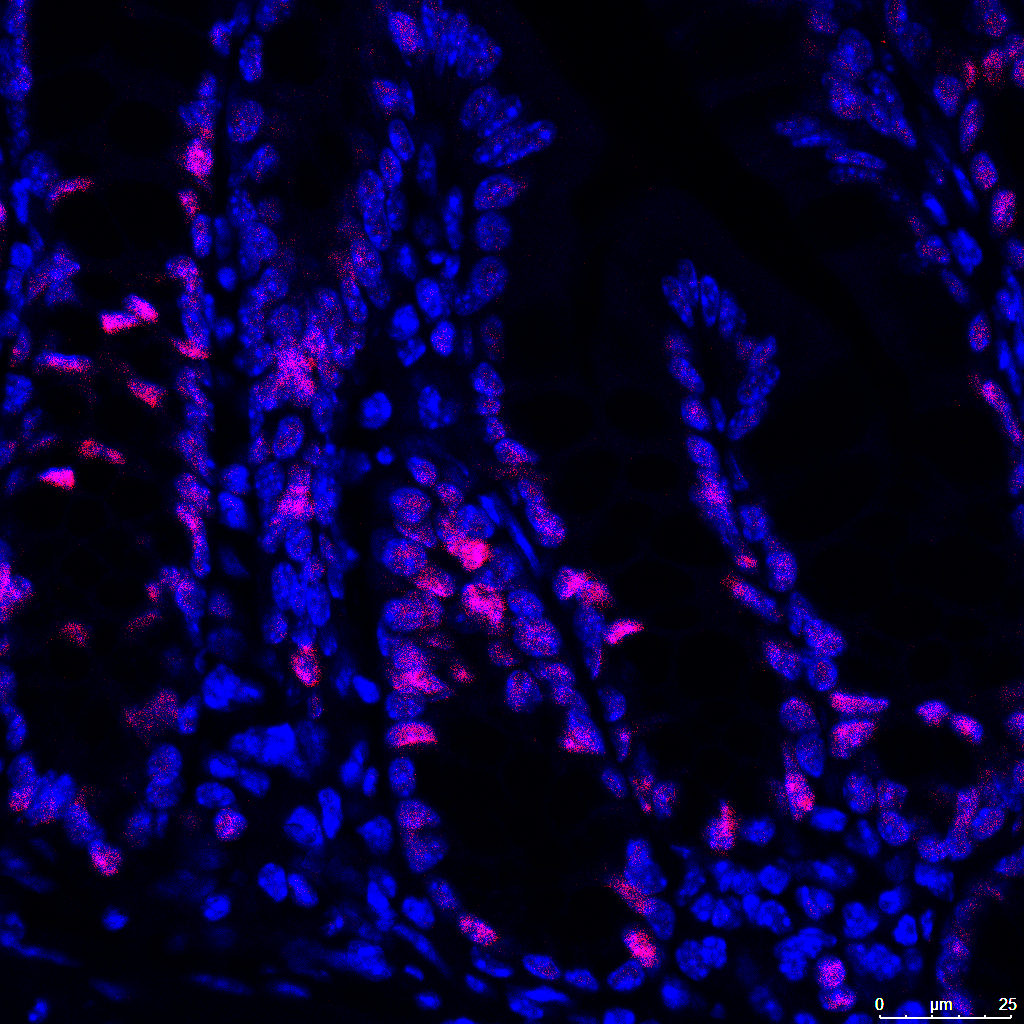

Supplement: Supplementary file 8 [file Data_Sheet_8.ZIP › fig6 raw data-1/Image wt ki67.tif]

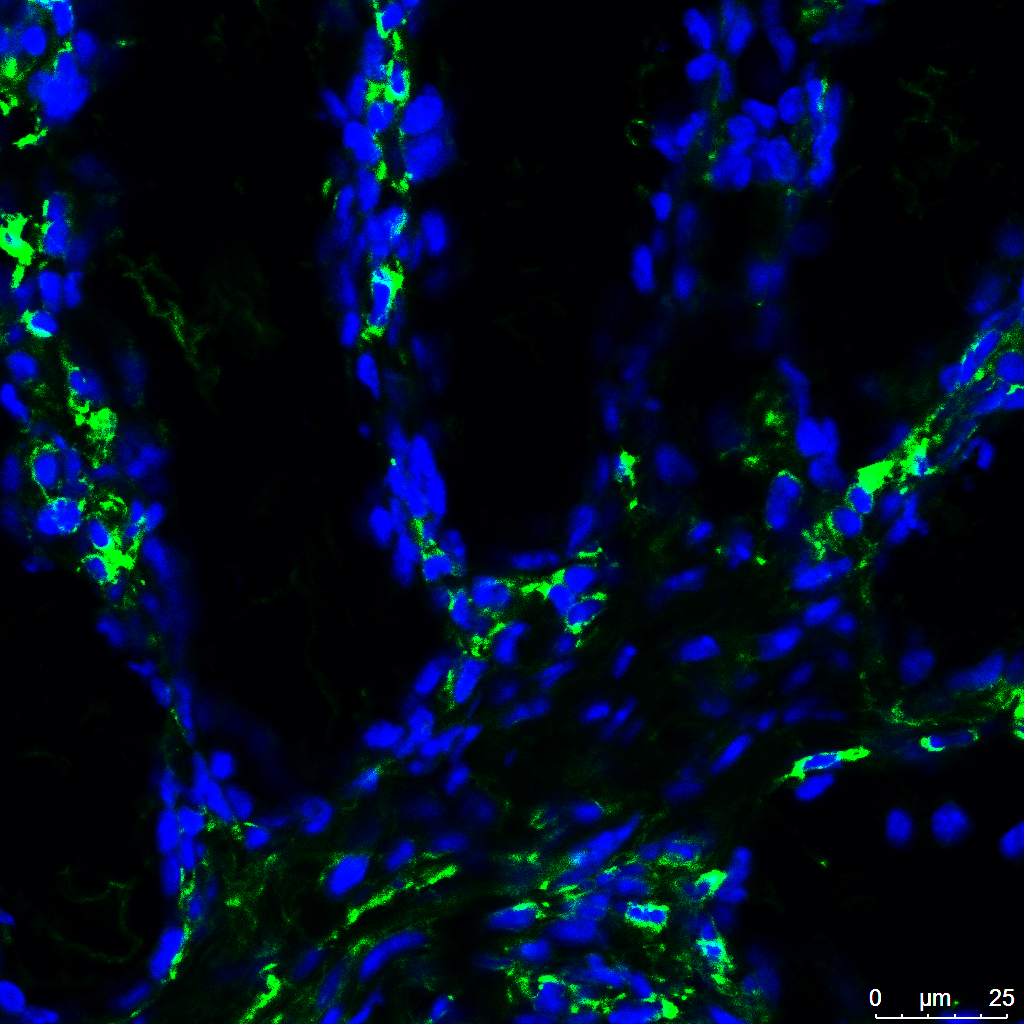

Supplement: Supplementary file 8 [file Data_Sheet_8.ZIP › fig6 raw data-1/WT lgr5.tif]

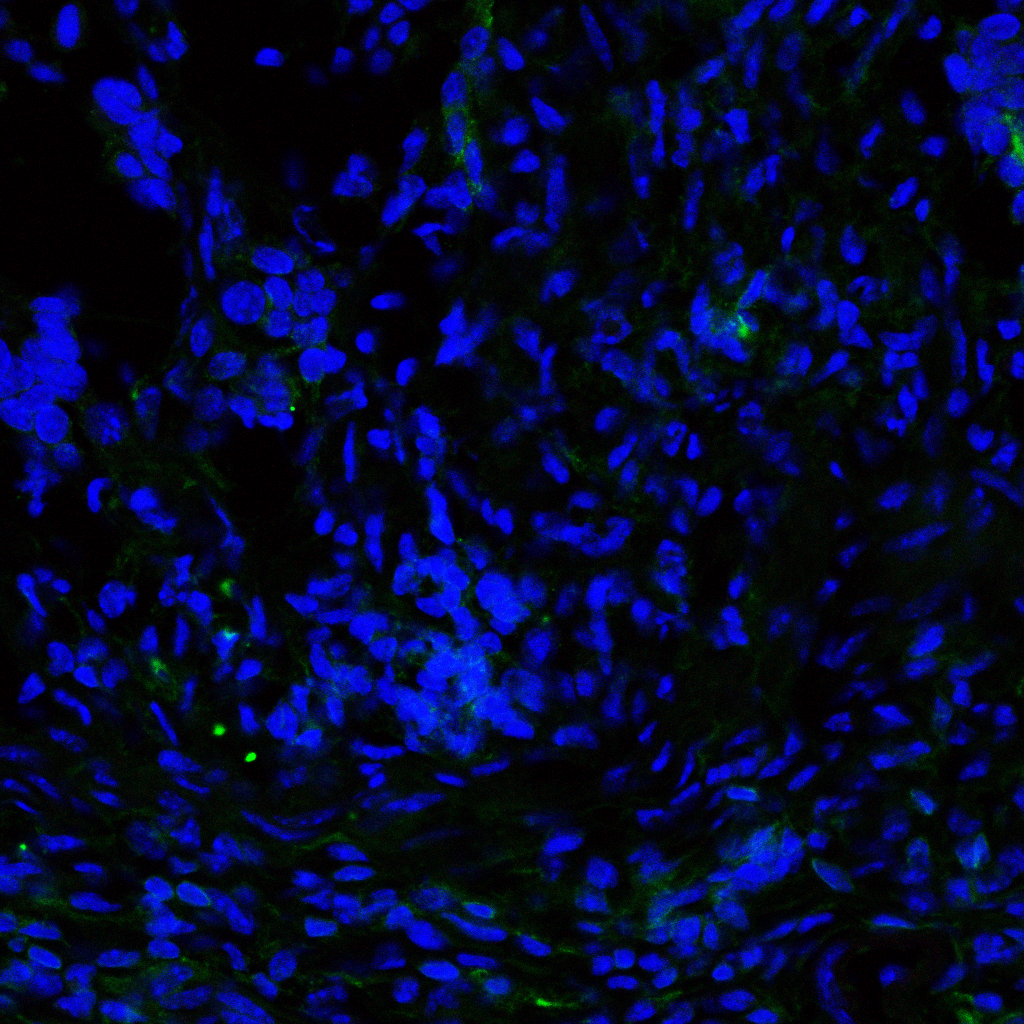

Supplement: Supplementary file 8 [file Data_Sheet_8.ZIP › fig6 raw data-1/bmp4inid3_Image002.tif]

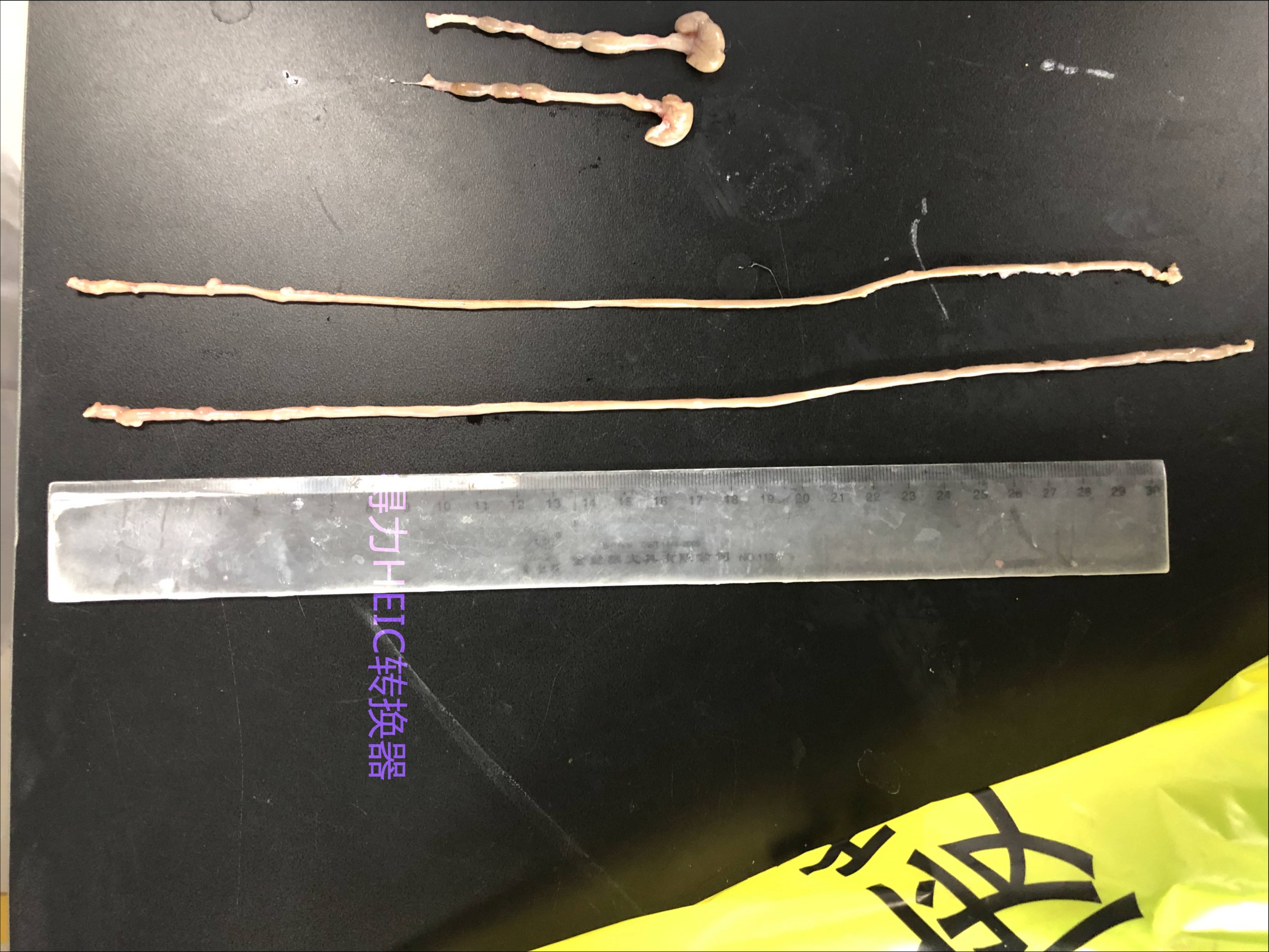

Supplement: Supplementary file 8 [file Data_Sheet_8.ZIP › fig6 raw data-1/ko IMG_4589_1604888933.jpg]

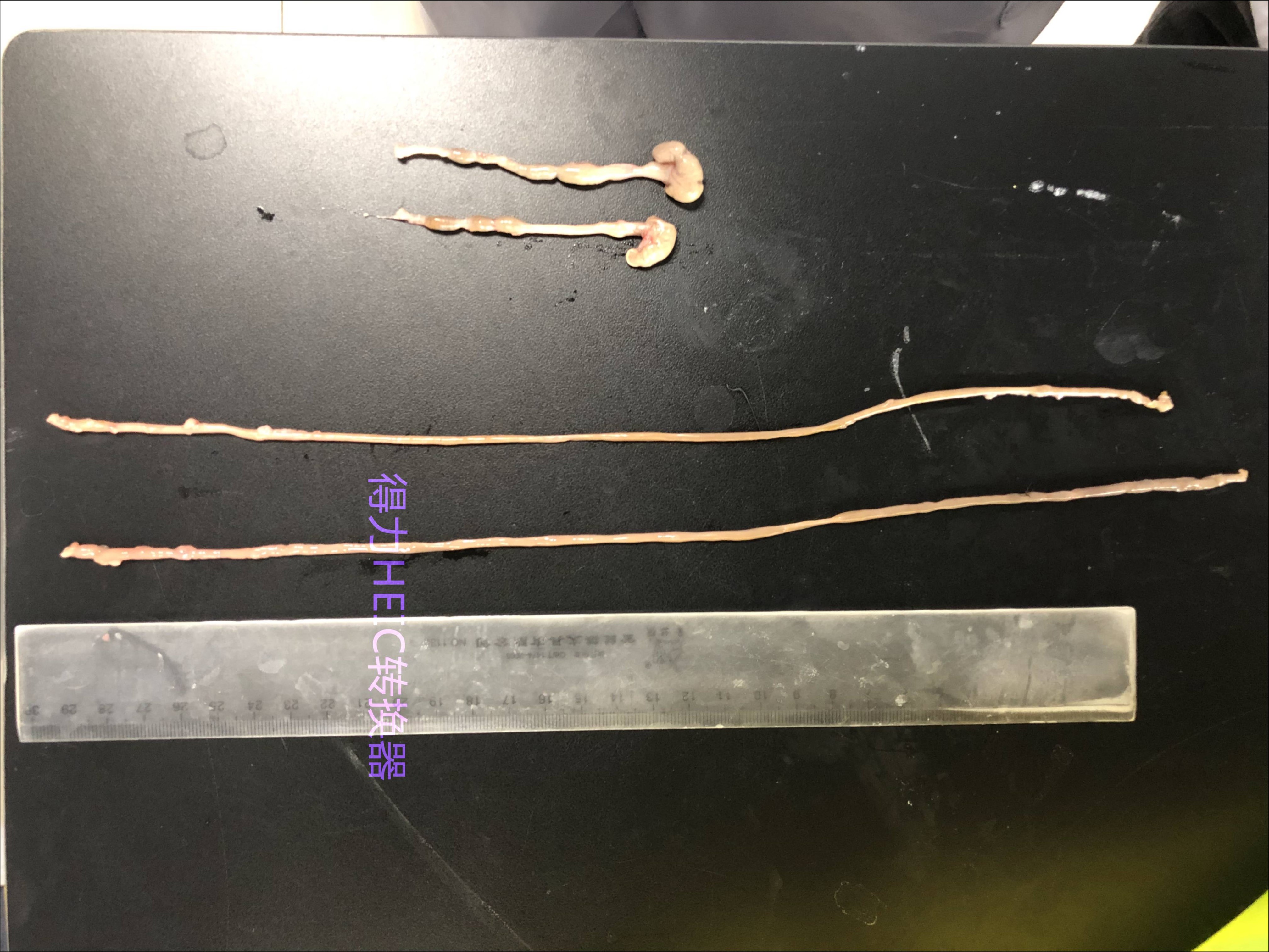

Supplement: Supplementary file 8 [file Data_Sheet_8.ZIP › fig6 raw data-1/wt IMG_4587_1604888933.jpg]

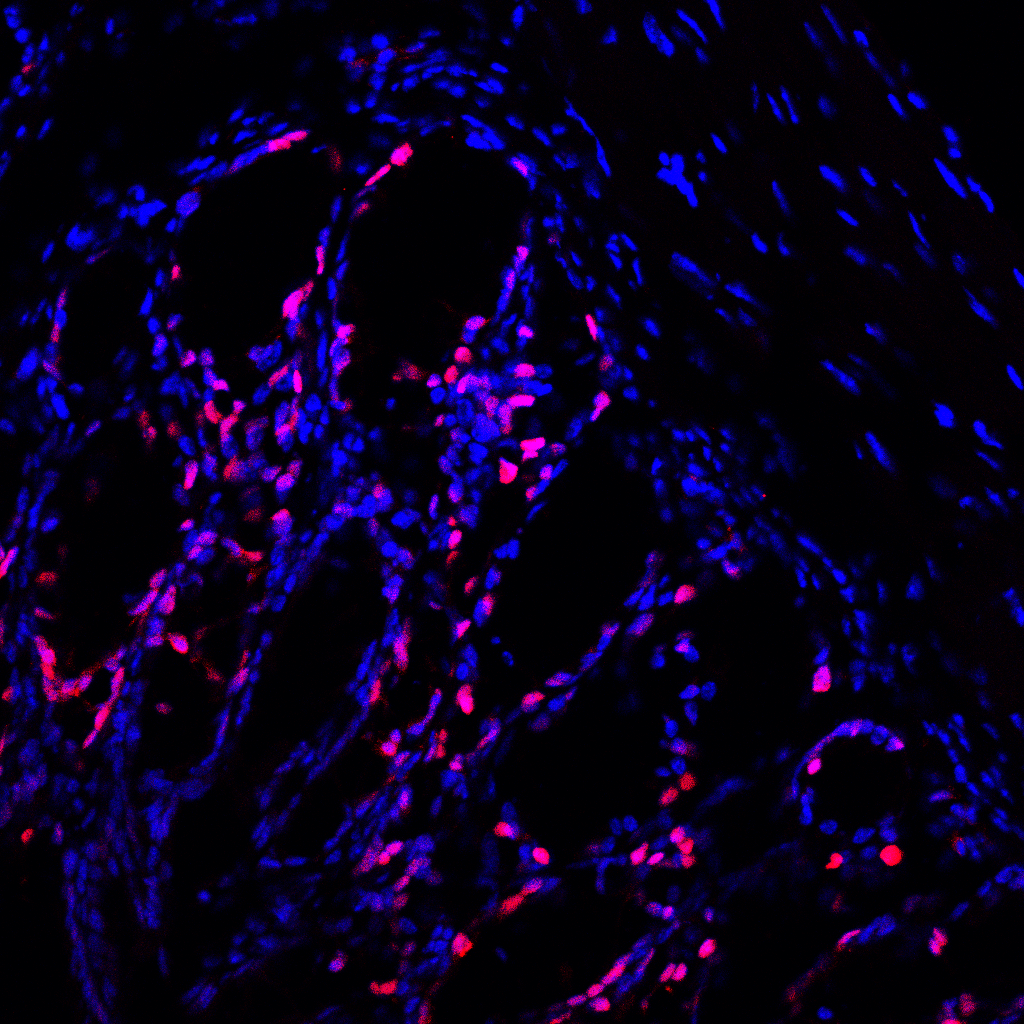

Supplement: Supplementary file 8 [file Data_Sheet_8.ZIP › fig6 raw data-1/wt-ki67_Image002_ch02.tif]

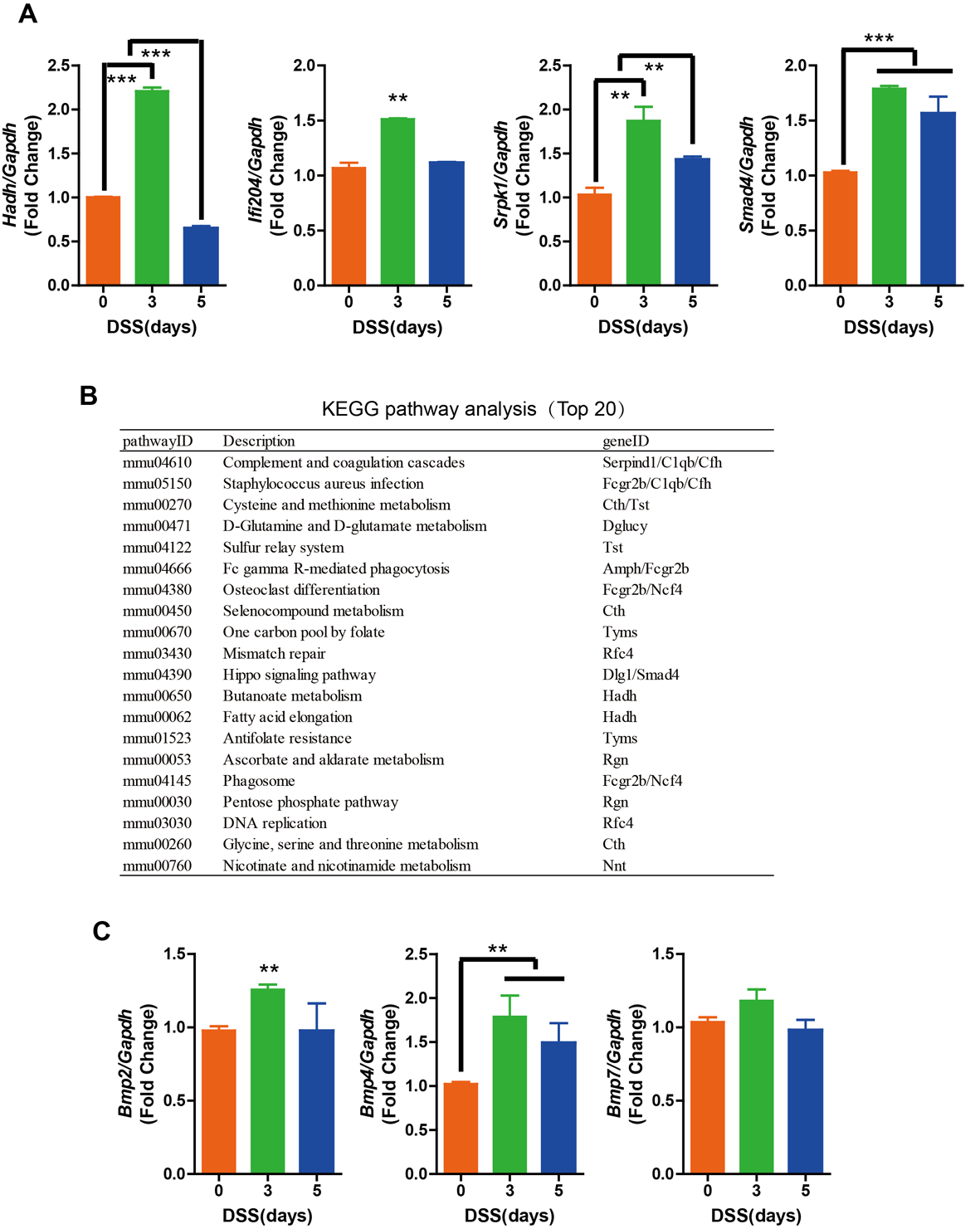

Supplement: Supplementary file 11 [file Image_1.TIF]

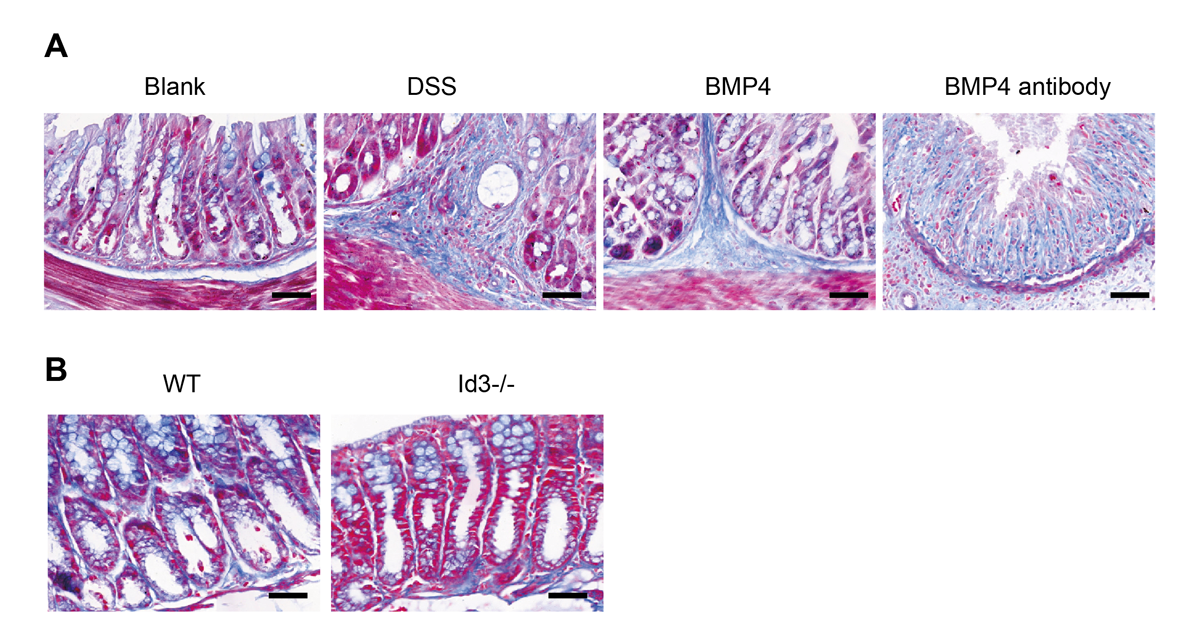

Supplement: Supplementary file 12 [file Image_2.TIF]
